# Light effect on Click reaction: Role of photonic quantum dot catalyst

**DOI:** 10.1038/srep33025

**Published:** 2016-09-13

**Authors:** Debkumar Nandi, Abu Taher, Rafique Ul Islam, Meenakshi Choudhary, Samarjeet Siwal, Kaushik Mallick

**Affiliations:** 1 Department of Chemistry, University of Johannesburg, P.O. Box: 524, Auckland Park 2006, South Africa

## Abstract

Due to the light excitation, the valence band electron of the copper (I) sulfide quantum dot transfer to the conduction band and act as a scavenger of the terminal proton of the alkyne in the presence of organic azide with the formation of 1,4-disubstituted 1,2,3-triazoles, where the copper(I) species of Cu_2_S act as a catalyst for the reaction. The above cycloaddition reaction between alkyne and azide is commonly known as the Click reaction. In this study, experiments were carried out under the exposure of ultra-violate and daylight and also dark environment. According to the original recommendation for the Click reaction, the role of the base was also considered for this experiment. We found that the effect of conduction band electron is more efficient than the recommended conventional base mediated reaction procedure.

Light induced catalytic organic transformation reaction is a new direction of research where the electronic property of the catalyst can play an important role with or without altering the mechanistic pathway and by eliminating one or more components (preferable hazardous) required for the reactions under normal condition.

Efforts have been made for the light induced metal oxide catalyzed organic transformation reactions where the conduction band electron has contribution for the reaction. The photocatalytic activity of these metal oxides is usually associated with non-selective free radical reaction processes. The photocatalytic oxidation of benzyl alcohol and its derivatives to the corresponding aldehydes proceeded at high conversion and selectivity on a TiO_2_ photocatalyst under light irradiation in presence of oxygen atmosphere^1^. The photocatalytic aerobic oxidation of benzyl amines to corresponding imines has also been reported on TiO_2_ under UV-irradiation^2^. Semiconductor Nb_2_O_5_ can also serve as a platform for the oxidation of alcohols through the light-induced formation of an excited electron in the conduction band and the positive hole in the valence band^3^.

The surface plasmon resonance effect of nanostructured materials also has substantial contribution on the light induced catalytic activity for various reactions. Unique optoelectronic and chemical properties of metal nanoparticles have drawn enormous attention of chemists, physicists and biologists for the fabrication of new generation devices. Some interesting reports on the Suzuki coupling reaction have been published based on the plasmonic effect of the gold nanoparticles at room temperature under visible light irradiation, where the role of gold is to increase the electron density of the catalytically active palladium to activate the aryl halides^4^,^5^,^6^. Similarly, photo induced charge transfer from graphitic carbon nitride to palladium is also reported for room temperature Suzuki coupling reaction^7^. The Suzuki reaction under ambient condition with equivalent amount of yield, as above, is also well documented in various reports^8^,^9^,^10^.

Motivated by the above reports, we have carried out the experiment to explore the photonic effect on the copper catalyzed azide-alkyne cycloaddition reactions^11^,^12^ for the preparation of 1,4-disubstituted regioisomeric 1,2,3-triazole compounds, also known as a Click reaction, which has a wide range of applications in organic synthesis, bio- and medicinal chemistry and materials science^13^. The Click reaction can be performed under an extremely wide variety of copper species^14^,^15^,^16^,^17^,^18^,^19^ in the presence of basic condition. In connection with our on-going research on the development of effective catalysts for organic transformations^20^,^21^,^22^,^23^, we have reported for both catalyst synthesis and cycloaddition reaction in a one-pot method^24^ and Cu (I)-polyaminobenzoic acid catalyzed azide-alkyne cycloaddition reaction^25^. Again, solvent-less microwave irradiation technique was also been reported for a 1,3-dipolar cycloaddition reaction between terminal alkynes and azides to synthesize 1,2,3-triazoles using polymer supported copper(I) composite, fabricated using a one-step chemical synthesis route under ambient condition^26^. Protocols have also been reported for the photoinduced formation of Cu(I) using graphitic carbon nitride^27^, fullerene^28^ and bimetallic complex comprising a photosensitizer ruthenium unit^29^ as a photocatalyst, from the Cu (II) precursor in the presence of amine as a base molecule for azide-alkyne cycloaddition reaction.

In this present communication, we report a convenient two step method for the synthesis of copper sulfide (Cu_2_S) quantum dots where the first step involved the copper sulphate mediated synthesis of polyaniline with the formation of Cu(I)-polyaniline composite followed by the synthesis of Cu_2_S nanoparticles by addition of thiourea, a source of sulfer, used for the synthesis of various metal sulfides^30^. Copper(I) sulfide (Chalcocite) is a *p*-type semiconductor with a direct band gap of 1.8 eV and an indirect bandgap of 1.2 eV^31^,^32^,^33^ and has wide applications in solid-state electronics^34^,^35^,^36^. In the literature, reports has been published for the synthesis of copper (I) sulfide by using different techniques^37^,^38^,^39^,^40^,^41^,^42^.

The polyaniline stabilized Cu_2_S nanoparticles, showed a high catalytic activity towards azide-alkyne 1, 3-dipolar cycloaddition reactions under ultra-violet (UV) irradiation condition. The following comparative studies were also performed under different photonic environment, such as, under daylight (DL) and daylight in presence of base (DLB) also under dark condition in absence of base (D) and dark condition in presence of base (DB), at room temperature.

## Result

### Mechanism of Cu_2_S formation and characterization

Copper sulphate mediated synthesis of polyaniline is an example of ‘*in-situ* polymerization and composite formation’ (IPCF) technique, where the organic component (aniline) and the metal salt (CuSO_4_, 5H_2_O) was chosen to facilitate such kind of reactions. The mechanism of the IPCF type of polymerization involve the release of electrons during the reaction between monomer and metal salt (oxidizing agent). In general, the released electrons reduce the metal ions, such as, gold, silver and palladium, with the formation of their corresponding nanoparticles^43^,^44^,^45^. However, in the present experiment we found the reaction between aniline and copper sulphate evidences the formation of Cu(I) and polyaniline^24^,^26^. Aniline have several amine as well as imine moieties which can act as a macro ligand^46^, that coordinate with the Cu(I) species^24^. The partial reduction of Cu(II) to Cu(I) species which is similar to the transformation from Cu(II) to Cu(I) species by terminal alkynes in the presence of tetramethylethylenediamine^47^. The Cu(I)-polyaniline subsequently forms polyaniline stabilized Cu_2_S quantum dots by the addition of thiourea.

**Figure 1:**
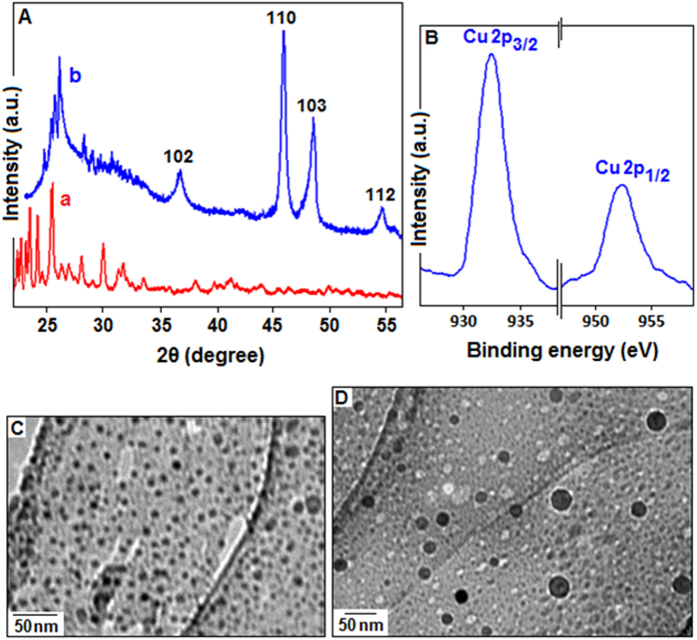
(**A**) The X-ray diffraction pattern of Cu(I)-polyaniline composite (a) and the polyaniline stabilized Cu_2_S nanoparticles (b), where four broad peaks at 36.90°, 46.20°, 48.50° and 54.80° correspond to (102), (110), (103) and (112) lattice plane. (**B**) X-ray photoelectron spectroscopy for Cu2p shows the binding energies of the photoelectron peaks of Cu 2p_3*/*2_ and 2p_1*/*2_ are 932.5 eV and 952.5 eV, respectively, for the polymer stabilized Cu_2_S. (**C,D**) TEM images, with two different magnification, of the polyaniline stabilized copper sulfide quantum dots.

The X-ray diffraction (XRD) patterns show (Fig. 1) the difference between Cu(I)-polyaniline and polyaniline stabilized Cu_2_S. Figure 1(a) shows the X-ray diffraction of the Cu (I)-polyaniline composite and the pattern of the polyaniline generally depend on the length of the polymeric chains, oxidation states, synthetic routes, solvent used and oxidizing agent used for the synthesis. Figure 1(b) is the XRD for the polyaniline stabilized Cu_2_S quantum dots where the pattern shows four broad peaks at 36.90°, 46.20°, 48.50° and 54.80°originating from (102), (110), (103) and (112) lattice plane, which are close to those reported for chalcocite Cu_2_S^42^. X-ray photoelectron spectroscopy (XPS) analysis for the sample containing Cu_2_S shows (Fig. 1B) the binding energies of the photoelectron peaks of Cu 2*p*_*3/2*_ and 2*p*_*1/2*_ are 932.5 eV and 952.5 eV, respectively, and is well consistent with the standard reference XPS spectrum of Cu 2p in Cu(I)^48^. The transmission electron microscopy image showed the evidence for the formation of copper sulfide quantum dots (dark spots) with size distributions ranging from 10 to 40 nm (Fig. 1C,D). The synthesized material (polymer stabilized Cu_2_S) was used as a catalyst for azide-alkyne cycloaddition reaction under different optical environment and the optimization study of the reaction was carried out as follows.

### Optimization study for Click reaction

**Figure 2:**
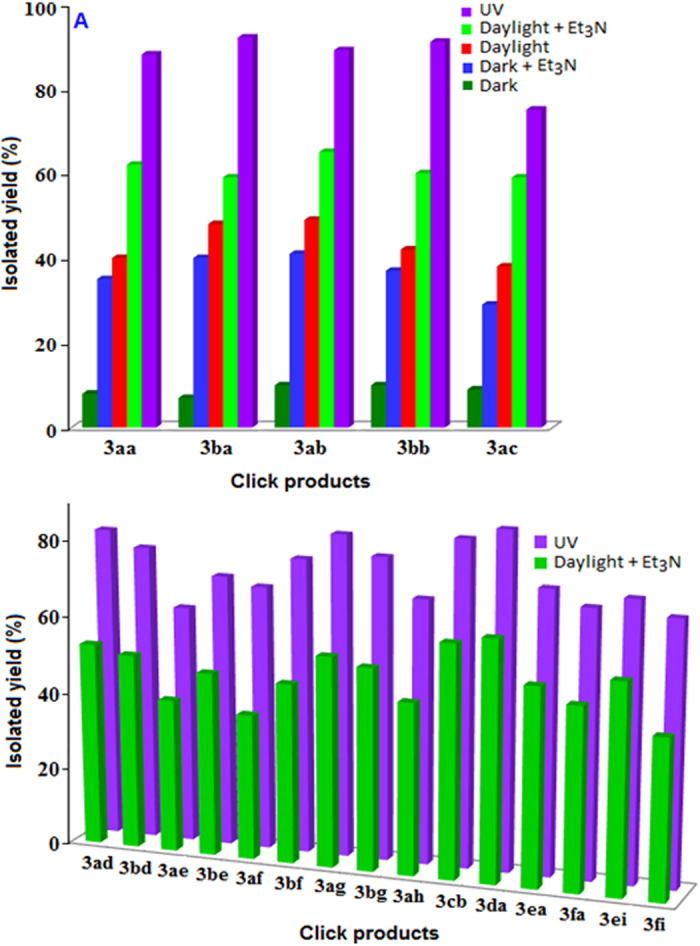
(**A**,**B**) Graphical representations show the azide-alkyne cycloaddition product (yield %) under different optical and basic condition.

**Figure 3:**
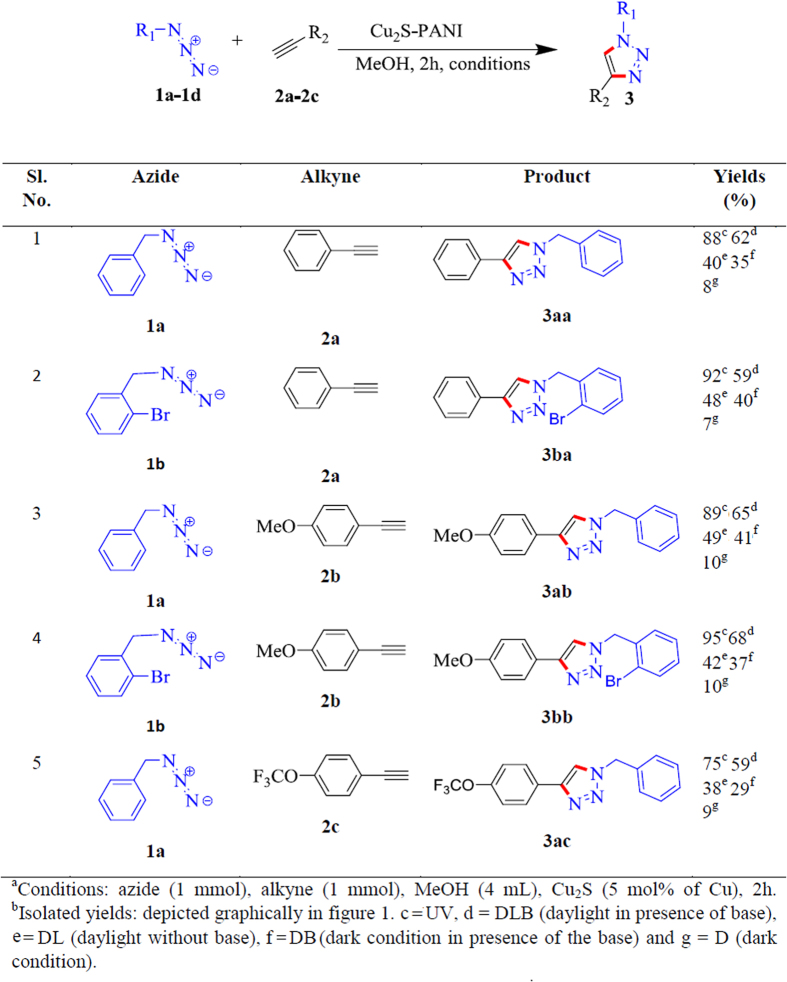
Optimization of Photonic effect and effect of base on click products^a,b^.

Optimization study has been shown in Figs 2A and 3. The reaction between azidomethyl benzene (**1a**) and ethynylbenzene (**2a**) yielding 1-benzyl-4-phenyl-1H-1,2,3-triazole (**3aa**) in presence of daylight was chosen as model reaction for the optimization study (Fig. 3, entry 1). Among the various solvents (CHCl_3_, MeOH, MeOH:H_2_O in different ratio, H_2_O and THF) used for the reaction we found methanol appeared to be the best solvent yielding 40% of desired product at 120 min. In the next step, we have examined the effect of base on the performance of the reaction and tri-ethylamine (Et_3_N) was recognized as optimized base for the above reaction as compared with other bases (K_2_CO_3_, Cs_2_CO_3_, KOH, N_2_H_4_.H_2_O, di-ethyl ammine, di-isopropyl ammine) which resulted a yield of 62% of the product for the same period of time. Furthermore, we are interested to check the photonic effect on the reaction. We have performed the reaction under dark environment both in the absence and presence of base and obtained the product with the yield of 8 and 35%, respectively. Interestingly, when reaction was performed under UV-irradiation a yield of 88% product was obtained in the absence of base. A similar trend has also been observed for the **3ba**, **3ab**, **3bb**, **3ac** as represented graphically in Fig. 2A and in Fig. 3 (entries: 2–5) and discussed in the following section. It is pertinent to mention that a decrease of yield has been observed by addition of base under UV-irradiation for this reaction. In the absence of catalyst no coupled product was noticed.

### Click reaction under different optical conditions

The cycloaddition reaction using polyaniline stabilized Cu_2_S as catalyst in the presence and absence of Et_3_N has been described below under different photonic conditions for other azide and alkyne molecules for the period of 120 min (Fig. 3: entries 2–5, Fig. 2A). In the presence of UV, azidomethyl benzene (**1a**) reacted with 1-ethynyl-4-methoxybenzene (**2b**) and 1-ethynyl-4-(trifluoromethoxy) benzene (**2c**) produced the cycloaddition products of 1-benzyl-4-(4-methoxyphenyl)-1H-1,2,3-triazole (**3ab**) and 1-benzyl-4-(4-(trifluoromethoxy) phenyl)-1H-1,2,3-triazole (**3ac**) with the yield of 89 and 75%, respectively. Under the DL condition, when the above reactions were performed in the presence and absence of base the product **3ab** with 65 and 49% of yields, respectively, and the product **3ac** with 59 and 38% of yields, respectively, were obtained. Again, when the above reactions were carried out under the dark condition both in the presence and absence of base the product **3ab** with 41 and 10% of yields, respectively, and 3ac with 29 and 9% of yields, respectively, has been obtained. A similar effect has also been documented when 1-(azidomethyl)-2-bromobenzene (**1b**) reacted with ethynylbenzene (**2a**) and 1-ethynyl-4-methoxybenzene (**2b**), the formation of cycloaddition products 1-(2-bromobenzyl)-4-phenyl-1H-1,2,3-triazole (**3ba**) and 1-(2-bromobenzyl)-4-(4-methoxyphenyl)-1H-1,2,3-triazole (**3bb**) with the yield 92 and 95%, under UV exposure, followed by 59 and 68% of yields under DLB, respectively, were obtained. It is also important to mention that under DL 48 and 42% of yield of **3ba** and **3bb**, respectively, have been produced. Under the darkroom environment, 40 and 7% of **3ba** and 29 and 9% of **3ac** has been formed in the absence and presence of base, respectively. Based on the above studies, we found the maximum amount of products were formed under UV-irradiation followed by under DLB condition.

### Versatility of the title reaction

**Figure 4:**
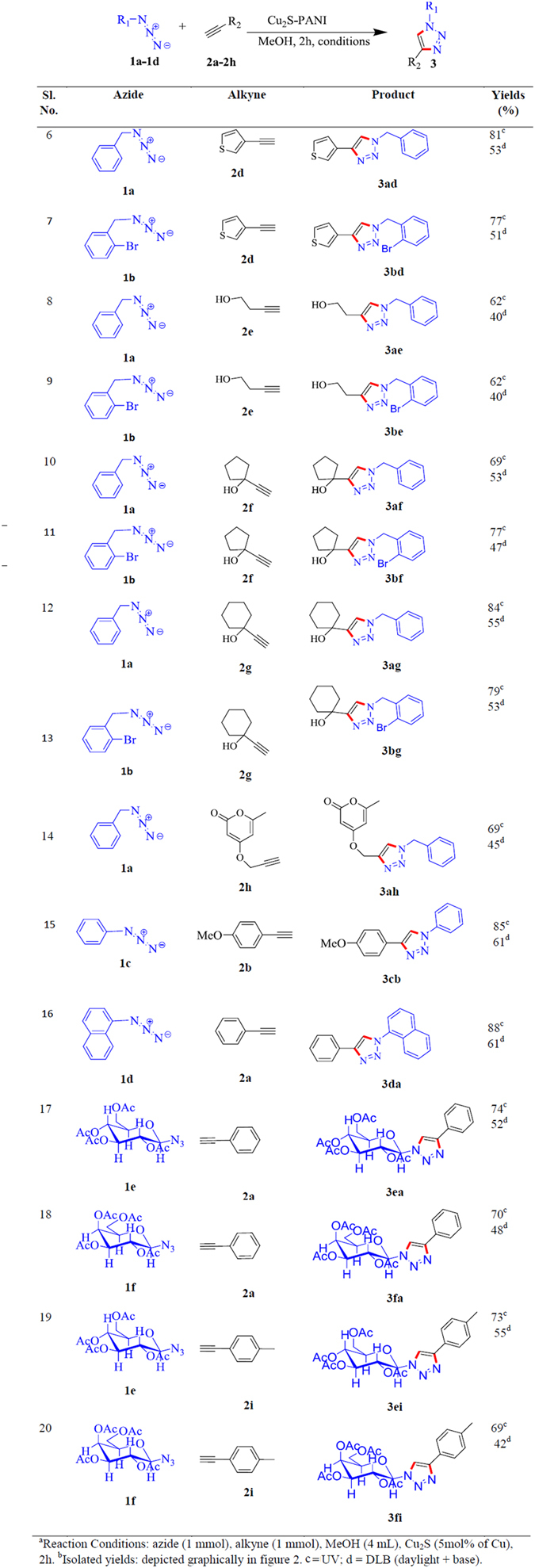
Substrate scope and comparative studies of yields of the click products in day light with tri-ethyl ammine and under base free UV irradiation^a,b^.

We also have explored the versatility of the reaction for the structurally diverse azides and alkynes under two different conditions where the maximum yields were obtained and the results are summarized in Fig. 4 and also graphically (Fig. 2B). More than 75% of yield of thiophene substituted triazole (**3ad** and **3bd**) product has been achieved due to the coupling between 3-ethynylthiophene and azide under UV irradiation whereas in the presence of base under daylight condition the maximum 53% of yield has been obtained for the same reaction (Fig. 4, entries: 6 and 7). For the reactions between azides with terminal alkyne group containing aliphatic alcohols (entries: 8, **3ae** and 9, **3be**), five (entries: 10, **3af** and 11, **3bf**) and six (entries: 12, **3ag** and 13, **3bg**) membered cyclic alcohols where UV-irradiation plays the superior role as compared with the base accompanied daylight condition for the formation of substituted triazole products. In the presence of UV, terminal alkyne derivatized unsaturated lactone (**2h**) with azide molecule (**1a**) also forms cycloaddition product, 4-((1-benzyl-1H-1,2,3-triazol-4-yl)methoxy)-6-methyl-2H-pyran-2-one (**3ah**), with the yield of 69% whereas the daylight mediated reaction under basic condition generate 45% of product (entry 14). Substituted ethynylbenzene with azidobenzene and ethynylbenzene with azidonaphthalene forms cycloaddition products of 4-(4-methoxyphenyl)-1-phenyl-1H-1,2,3-triazole (**3cb**) and 1-(naphthalen-1-yl)-4-phenyl-1H-1,2,3-triazole (**3da**), respectively, entries 15 and 16, where we have again observed the supremacy of the ultra-violate effect regarding the amount of product formation.

### Examples with the sugar molecules

Azide-alkyne 1,3-dipolar cycloaddition reaction, serves as the most powerful tool for the creation of complex architecture at the molecular level. The unique properties of the carbohydrate moiety and the advantages of chemo- and regio-selective nature of the Click chemistry produce biologically relevant glycoconjugate type of molecules for various biological and pharmaceutical applications. In the current study we have synthesized glucose and galactose substituted azide molecule and we have found that the substituted azide molecule coupled with alkyne molecules to form β-D-glucopyranoside and β-D-galactopyranoside substituted 1,2,3-triazole moieties, respectively, under the exposure of the ultra-violate light in the presence of Cu_2_S catalyst. A comparative study shows (Fig. 4, entries 17–20 and Fig. 2B, **3ea**, **3fa**, **3ei** and **3fi**) that the reaction under the UV exposure again showed the superior result than the reaction under daylight condition in the presence of triethyl amine. When 1-azido-2,3,4,6-tetra-*O*-acetyl-β-D-glucopyranose (**1e**) and 1-azido-2,3,4,6-tetra-*O*-acetyl-β-D-galactopyranose (**1f**) reacted with the alkyne molecule, ethynylbenzene (**2a**), the coupled product 4-Phenyl-1-(2,3,4,6-tetra-*O*-acetyl-β-D-glucopyranosyl)-1*H*-1,2,3-triazole (**3ea**), entry: 17, and 4-Phenyl-1-(2,3,4,6-tetra-*O*-acetyl-β-D-galactopyranosyl)-1*H*-1,2,3-triazole (**3fa**), entry: 18, obtained with the yield 90% and 70%, under UV and DLB conditions, respectively. A similar trend has also been found when 1-ethynyl-4-methylbenzene was used as alkyne molecule (entry: 19, **3ei**, and 20, **3fi**).

### Click reaction for single-step multicomponent system

**Figure 5:**
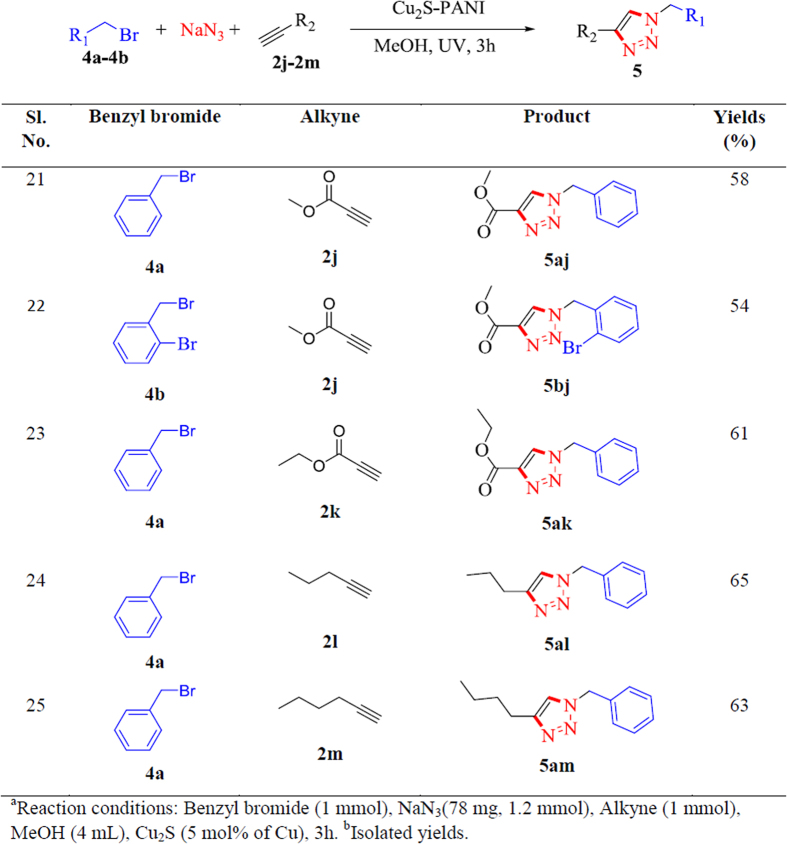
Substrate scope for multicomponent click products under UV irradiation^a,b^.

In general, copper catalysed azide-alkyne cycloaddition reaction is a two component reaction system where the organic azides need to be synthesized in advance. Single-step reactions have many advantages in comparison with multi-step reactions, primarily due to environmental and economic considerations, and therefore the design of a single-step multicomponent reaction system has attracted lot of attention from the research groups in various areas of organic synthesis. For organic reactions the steps are not as efficient enough as enzymatic reactions. So, the strategy should be adopted carefully for the preparation of complex substances in which controlled formation of bonds occurs in a single step from the readily available precursors. For the current study, we also directed our attention to develop an efficient one-pot methodology that uses alkyl halides and sodium azide for direct cycloaddition with alkynes in the presence of Cu_2_S catalyst under the UV-irradiation. The Cu_2_S catalyst again performed well as a catalyst in methanol under the UV-irradiation to give the desired products (Fig. 5, entries 21–25, **5aj**, **5bj**, **5ak**, **5al** and **5am**, respectively) with the isolated yields ranging from 54 to 65% within 3 h. In presence of base under daylight condition, a longer time (5 h) required to achieve the equivalent amount of yield for the corresponding products.

### Mechanistic discussion for the reaction

**Figure 6:**
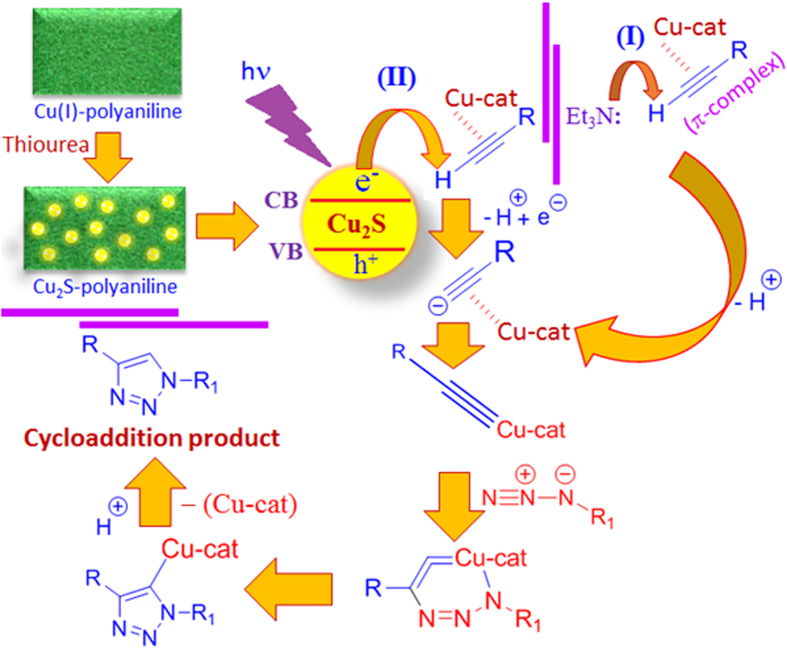
Schematic mechanism for the Cu_2_S catalysed azide-alkyne cycloaddition reaction in presence of base, Et_3_N, (I) and under UV or daylight irradiation (II) condition.

In general, Cu (I) catalyzed cycloaddition reaction, between azide and alkyne, happened under basic condition and illustrated in Fig. 6. Initially, a π-complex has formed between the Cu (I) and the alkyne molecule, results in the lowering of the pKa value of the terminal acetylene, [11, 12] which facilitate the proton abstraction mechanism from the C–H bond in the presence of a base, triethyl amine used as a base in this current study, Fig. 6(I), with the formation of copper-acetylidine complex followed by the addition of the azide molecule to form an intermediate complex. The intermediate complex subsequently forms the 1,2,3-triazole after the protonation and elimination of the catalyst.

In this study Cu_2_S was used as the active catalyst and when the reaction was carried out under the exposure of UV-irradiation in absence of base the highest amount of cycloaddition was obtained. The photon energy from the ultraviolet source, deep UV (UV-C), is within the range of 6.53–4.43 eV (considering the wavelength between 190–280 nm) and which is the sufficient amount of energy to transfer an electrons from the valence band to the conduction band of Cu_2_S quantum dot. The conduction band electron act as a scavenger of the terminal proton of the alkyne with the formation of copper-acetylidine complex, Fig. 6(II), and follows the similar mechanistic route as mentioned above. We also found that the daylight has a prominent effect on the reaction as the photon energy value of daylight is in between 3.26–1.59 eV (considering all the visible wavelength range from 380–780 nm). This photon energy is sufficient to facilitate the electron migration from the valence band to the conduction band and follow the Fig. 6(II). The difference in yield for the cycloaddition reaction between the ultra-violate and daylight condition can be justified as follows. As the photon energy of the UV-light is higher than the daylight so the higher population of electron would be available in the conduction band of Cu_*2*_S for scavenging the terminal proton of the alkyne that consequently produce higher amount of cycloaddition product. The effect of daylight in presence of base shows the enhancement of the cycloaddition product than the daylight alone which is due to the combined effect of conduction band electron and lone pair electron of the base, both can participate the proton abstraction mechanism.

The Click reaction was also performed under darkroom environment in the presence and absence of base to find out the photonic effect on the reaction performance. Comparative study showed the daylight condition in absence of base performs slightly better than the presence of base under the dark condition, which again indicates the conduction band electron plays the dominant role for Cu_2_S catalyzed cycloaddition reaction. In the absence of a base (under darkroom environment) the formation of little cycloaddition product (~10%) has also been noticed possible due to the presence of uncoordinated emeraldine form of polyaniline (emeraldine base) which act as the role of base to facilitate the reaction.

It is pertinent to mention that a decrease of yield has been observed by addition of base under UV-irradiation for this reaction and the mechanism of this event is included elsewhere in the manuscript. A decrease of product yield has been observed when the reaction was carried out under UV irradiation in presence of the base (triethyl amine) where the base can act as a hole-scavenger and also coordinate with the Cu(I) to form a complex^49^. The formation of the new complex may limit the transfer mechanism of electron from valence band to conduction band which consequently has an adverse effect on the yield.

## Conclusion

In this report, we have presented a ‘two-step method’ for the synthesis of polyaniline stabilized copper sulfide nanoparticles where the first step involved the formation of Cu (I)-polyaniline composite followed by the formation of polymer stabilized Cu_2_S photonic quantum dots. In the presence of ultra-violate radiation (high photon energy) the conduction band electron of the copper sulfide involves in the azide-alkyne cycloaddition reaction and plays the crucial role by participating in the ‘proton abstraction mechanism’ by scavenging the terminal proton of the alkyne molecule. The similar kind of photonic effect is also possible due to the presence of daylight (low photon energy) as the copper sulfide is a low band gap semiconductor. Click reaction under UV-illumination condition has the potential advantage on the environmental point of view, where the use of hazardous base molecule can be avoided. Another significant aspect for the UV-light mediated reaction is the rapid product formation. Photo-generated conduction band electron mediated reaction is faster than the conventional base molecule mediated reaction. It is also important to mention that many of the organic functional groups are base sensitive thus the use of base molecule in Click reaction may be an obstacle for achieving the targeted product. Present method has given an alternative route to those substrates for the targeted Click products and be beneficial for the reactions. The combined physical (photonic) and chemical (catalytic) properties of the Cu_2_S has changed the original reaction mechanism of the Click reaction by creating an alternatives route in the presence of light energy.

## Methods

### Preparation of Cu_2_S-polyaniline composite

In a typical experiment, 0.093 g of aniline (10^−2 ^mol dm^−3^) was dissolved in 10 mL methanol. To this solution, 0.250 g of CuSO_4_. 5H_2_O in 10 mL of water (10^−2^ mol dm^−3^) was added slowly under continuous stirring conditions. During the addition, the solution took on a green color, while at the end, a greenish precipitation was formed, indicates the formation of Cu (I)-polyaniline composite (24, 26), at the bottom of the conical flask. To this reaction mixture 5 mL of thiourea (10^−1 ^mol dm^−3^) was added and allowed to stir for 1 h. Entire reaction was performed under ambient condition. The dark brown precipitate material was allowed to settle for 30 min after which the colloidal solution was taken from the bottom of the conical flask and pipetted onto lacey, carbon-coated, nickel mesh grids for transmission electron microscopy (TEM) study. The remaining portion of the compound was dried under vacuum at 60 °C for XRD, XPS measurements and also applied as a catalyst for the azide-alkyne cycloaddition reaction.

### General Procedure for the Click reaction

In a 25 mL round bottom flask, azide**1** (1 equivalent), alkyne **2** (1 equivalent), were taken in 4 ml methanol. To this reaction mixture the dried polyaniline stabilized Cu_2_S catalyst (2 mg, 5 mol % of Cu) was added and stirred under the following conditions: (a) under UV light in the absence of base (b) under daylight with or without and Et_3_N (1 equivalent) and (c) under dark environment with or without and Et_3_N (1 equivalent) in 25 mL quartz reactor. The reaction mixture was stirred for 2 hand progress of the reaction was monitored using thin layer chromatography (TLC) technique. After completion, the reaction mixture was filtered and dried under residue pressure. The dried gummy mass was and diluted with 20 mL of distilled water; the solution was extracted with ethyl acetate. The organic layer was separated and dried over anhydrous MgSO_4_. Combined organic layer was concentrated in vacuum to give the corresponding triazole which was further purified by recrystallization or by column chromatography technique.

### Click reaction under luminescence condition

Philips UV-C (TUV T8) (germicidal) lamp has been used as the source of UV-light and a hand-held optical power meter (Newport) showed the optical intensity value was 40 mW/cm^2^ adjacent to the quartz reaction chamber. The optical intensity was also measured during the daylight experiment and was found a value of 3.5 mW/cm^2^ inside the fume hood.

### General Procedure for one pot multicomponent click reaction

In a 25 mL quartz reactor, benzyl bromide **4** (1 equivalent), alkyne **2** (1 equivalent) and NaN_3_ (78 mg, 1.2 equivalent) were taken in 4 ml methanol. To this reaction mixture Cu_2_S catalyst (5 mol% of Cu) was added and stirred under UV light for 3 h. After completion, the reaction mixture was filtered and dried under residue pressure. The dried gummy mass was and diluted with 20 mL of distilled water; the solution was extracted with ethyl acetate. The organic layer was separated and dried over anhydrous MgSO_4_. Combined organic layer was concentrated in vacuum to give the corresponding triazole which was further purified by recrystallization or by column chromatography technique. (NMR spectra for all the synthesized Click products are supplied in the supplementary information).

## Additional Information

**How to cite this article**: Nandi, D. *et al*. Light effect on Click reaction: Role of photonic quantum dot catalyst. *Sci. Rep.* **6**, 33025; doi: 10.1038/srep33025 (2016).

## Supplementary Material

Supplementary Information
