# Supplementary material for: Light effect on Click reaction: Role of photonic quantum dot catalyst
Source: Sci Rep. 2016 Sep 13;6:33025. doi: 10.1038/srep33025 (PMC5020494; doi:10.1038/srep33025)
Supplement: Supplementary Information [file srep33025-s1.doc]

*Supporting information*

**Light effect on Click reaction: Role of photonic quantum dot catalyst**

**Debkumar Nandi, Abu Taher, Rafique Ul Islam, Meenakshi Choudhary, Samarjeet Siwal, Kaushik Mallick***

Department of Chemistry, University of Johannesburg, P.O. Box: 524, Auckland Park 2006, South Africa.

List of contents

| **General Considerations** | **S1** |
| --- | --- |
| **Characterization of Products** | **S2-S9** |
| **Experimental References** | **S10** |
| **NMR Spectra** | **S11-S35** |

**General Considerations:**

Solvents were distilled from appropriate drying agent prior to use. Commercially available reagents were used without further purification unless otherwise stated. ^1^H and ^13^C NMR spectra were recorded on a Bruker AVANCE^III^-400 spectrometer.^1^H NMR (400 MHz) and ^13^C NMR (100 MHz) were registered in CDCl_3_ as solvent and tetramethylsilane (TMS) as an internal standard. Chemical shifts are reported in δ units (ppm). All coupling constants (*J*) are reported in hertz (Hz).

**Characterization of cycloadducts:**

**1-Benzyl-4-phenyl-1*H*-1,2,3-triazole** (**3aa**): ^1^ White solid, (UV: 207mg, 88%; Daylight + Et_3_N: 146mg, 62%; Daylight: 94mg, 40%; Dark+ Et_3_N: 82 mg, 35%; Dark: 19 mg, 8%) mp: 136-138^o^C. Synthesized following the general procedure from azidomethylbenzene **1a** (133 mg, 1.0 mmol), phnylacetelene **2a** (102 mg, 1.0 mmol), triethylamine (0.14 mL, 1 mmol). ^1^H NMR (400 MHz, CDCl_3_): δ 5.51 (s, 2H), 7.25-7.38 (m, 8H), 7.66 (s, 1H) 7.78 (d, *J*= 7.2 Hz, 2H); ^13^C NMR (100 MHz, CDCl_3_): δ 54.0, 119.5, 125.5 (2C), 127.8 (2C), 128.0, 128.6, 128.7 (2C), 128.9 (2C), 130.4, 134.5, 147.9.

**1-(2-Bromobenzyl)-4-phenyl-1*H*-1,2,3-triazole** (**3ba**): ^1^ White solid, (UV: 289mg, 92%; Daylight + Et_3_N: 185mg, 59%; Daylight: 151mg, 48%; Dark+ Et_3_N: 126 mg, 40%; Dark: 22 mg, 7%) mp: 110-112^o^C. Synthesized following the general procedure from 1-(azidomethyl)-2-bromobenzene **1b** (212 mg, 1.0 mmol), phnylacetelene **2a** (102 mg, 1.0 mmol), triethylamine (0.14 mL, 1 mmol). ^1^H NMR (400 MHz, CDCl_3_): δ 5.62 (s, 2H), 7.10 (dd, *J*=7.6, 1.6 Hz, 1H), 7.16(dt, *J*=7.6, 1.6 Hz, 1H), 7.21-7.30 (m, 2H), 7.36 (t, *J*=7.6, 2H), 7.56 (dd, *J*=7.2, 1 Hz, 1H) 7.78-7.80 (m, 3H); ^13^C NMR (100 MHz, CDCl_3_): δ 53.6, 119.8, 123.1, 125.5 (2C), 127.9, 128.0, 128.6, 129.9 (2C), 130.1, 130.2, 132.9, 134.0, 147.8.

**1-Benzyl-4-(4-methoxyphenyl)-1*H*-1,2,3-triazole** (**3ab**): ^1^ White solid, (UV: 236mg, 89%; Daylight + Et_3_N: 172mg, 65%; Daylight: 140 mg, 49%; Dark+ Et_3_N: 109 mg, 41%; Dark: 27 mg, 10%) mp: 144-146^o^C. Synthesized following the general procedure from azidomethylbenzene **1a** (133 mg, 1.0 mmol), 1-ethynyl-4-methoxybenzene **2b** (132 mg, 1.0 mmol), triethylamine (0.14 mL, 1 mmol). ^1^H NMR (400 MHz, CDCl_3_): δ 3.79(s, 3H), 5.52(s, 2H), 6.90 (d, *J* = 8.8 Hz, 2H), 7.26-7.35 (m, 5H), 7.56 (s, 1H), 7.70 (d, *J* = 8.8 Hz, 2H); ^13^C NMR (100 MHz, CDCl_3_): δ 54.1, 54.2, 114.1 (2C), 118.7, 123.2, 126.9 (2C), 127.9 (2C), 128.7, 129.0 (2C), 134.72, 148.0, 159.5.

**1-(2-Bromobenzyl)-4-(4-methoxyphenyl)-1*H*-1,2,3-triazole** (**3bb**): White solid, (UV: 313 mg, 91%; Daylight + Et_3_N: 206 mg, 60%; Daylight: 144 mg, 42%; Dark+ Et_3_N: 127 mg, 37%; Dark: 34 mg, 10%) mp: 156-158^o^C. Synthesized following the general procedure from 1-(azidomethyl)-2-bromobenzene **1b** (212 mg, 1.0 mmol), 1-ethynyl-4-methoxybenzene **2b** (132 mg, 1.0 mmol), triethylamine (0.14 mL, 1 mmol). ^1^H NMR (400 MHz, CDCl_3_): δ 3.80 (s, 3H), 5.66 (s, 2H), 6.91(d, *J* = 8.4 Hz, 2H), 7.13(d, *J* = 7.6 Hz, 2H), 7.20 (td, *J*=7.6, 1.2 Hz, 1H), 7.28 (t, *J*=7.5 Hz, 1H), 7.60 (d, *J* = 8.0 Hz, 1H), 7.72 (d, *J* = 8.4 Hz, 2H); ^13^C NMR (100 MHz, CDCl_3_): δ 53.6, 55.1, 114.1 (2C), 118.9, 122.9, 123.2, 126.9 (2C), 128.1, 130.0, 130.2, 133.0, 134.1, 147.8, 159.5.

**1-Benzyl-4(4-(trifluoromethoxy) phenyl)-1H-1,2,3-triazole (3ac)**: ^2^ White solid, (UV: 239mg, 75%; Daylight + Et_3_N: 188mg, 59%; Daylight:121mg, 38%; Dark+ Et_3_N: 92 mg, 29%; Dark: 29 mg, 9%) mp: 148-150^o^C. Synthesized following the general procedure from azidomethylbenzene **1a** (133 mg, 1.0 mmol), 1-ethynyl-4-trifluromethoxybenzene **2c** (186 mg, 1.0 mmol), triethylamine (0.14 mL, 1 mmol). ^1^H NMR (400 MHz, CDCl_3_): δ 5.52(s, 2H), 7.20 (d, *J* = 6.4 Hz, 2H), 7.26-7.35 (m, 5H), 7.81 (brs, 3H); ^13^C NMR (100 MHz, CDCl_3_): δ 54.2, 118.9, 121.2 (2C), 121.6, 126.9, 127.9 (2C), 128.7 (2C), 128.9 (2C), 129.3, 134.4, 148.7 (2C).

**1-benzyl-4-(thiophen-3-yl)-1H-1,2,3-triazole** (**3ad**): ^1^ White solid, (UV: 195mg, 81%; Daylight + Et_3_N: 128mg, 53%) mp: 149-151^o^C. Synthesized following the general procedure from azidomethylbenzene **1a** (133 mg, 1.0 mmol), 3-ethynylthiophene **2d** (108 mg, 1.0 mmol), triethylamine (0.14 mL, 1 mmol). ^1^H NMR (400 MHz, CDCl_3_): δ 5.50 (s, 2H), 7.24-7.26 (m, 2H), 7.30-7.33 (m, 4H), 7.38-7.39 (m, 1H), 7.64-7.68 (m, 2H); ^13^C NMR (100 MHz, CDCl_3_): δ 54.1, 120.9, 125.6, 126.2, 127.9 (2C), 128.5, 128.9 (2C), 129.4, 131.6, 132.7, 134.5.

**1-(2-Bromobenzyl)-4-(thiophen-3-yl)-1*H*-1,2,3-triazole** (**3bd**): ^1^ White solid, (UV: 246mg, 77%; Daylight + Et_3_N: 163mg, 51%) mp: 104-106^o^C. Synthesized following the general procedure from 1-(azidomethyl)-2-bromobenzene **1b** (212 mg, 1.0 mmol), 3-ethynylthiophene **2d** (108 mg, 1.0 mmol), triethylamine (0.14 mL, 1 mmol). ^1^H NMR (400 MHz, CDCl_3_): δ 5.64 (s, 2H), 7.10-7.13 (m, 1H), 7.17-7.28 (m, 2H), 7.31-7.33 (m, 1H), 7.41 (m, 1H), 7.57-7.59 (m, 1H), 7.63 (dd, *J* = 1.2, 7.6 Hz, 1H) 7.64(s, 1H); ^13^C NMR (100 MHz, CDCl_3_): δ 53.7, 119.6, 121.1, 123.3, 125.7, 126.2, 128.1, 130.1, 130.3, 131.6, 133.1, 134.1, 144.2.

**2-(1-Benzyl-1*H*-1,2,3-triazol-4-yl)ethanol** (**3ae**): ^3^ White solid, (UV: 124mg, 62%; Daylight + Et_3_N: 81mg, 40%) mp: 90-92^o^C. Synthesized following the general procedure from azidomethylbenzene **1a** (133 mg, 1.0 mmol), but-3-yn-1-ol **2e** (70 mg, 1.0 mmol), triethylamine (0.14 mL, 1 mmol). ^1^H NMR (400 MHz, CDCl_3_): δ 2.82 (t, *J* = 6.2 Hz, 2H), 3.77 (brs, 3H), 5.38 (s, 2H), 7.16-7.19 (m, 2H), 7.24-7.27 (m, 3H), 7.34 (s, 1H); ^13^C NMR (100 MHz, CDCl_3_): δ 28.5, 53.8, 60.9, 121.7, 127.8 (2C), 128.4, 128.8 (2C), 134.4, 145.5.

**2-(1-(2-bromobenzyl)-1H-1,2,3-triazol-4-yl)ethanol** (**3be**): Light yellow solid, (UV: 200mg, 71%; Daylight + Et_3_N: 135mg, 48%;) mp: 78-79^o^C. Synthesized following the general procedure from 1-(azidomethyl)-2-bromobenzene **1b** (212 mg, 1.0 mmol), but-3-yn-1-ol **2e** (70 mg, 1.0 mmol), triethylamine (0.14 mL, 1 mmol). ^1^H NMR (400 MHz, CDCl_3_): δ 2.88 (t, *J* = 6.0 Hz, 2H), 3.04 (brs, 1H), 3.86 (t, *J*=6.0 Hz, 2H), 5.57 (s, 2H), 7.08 (dd, *J*=7.6, 1.6 Hz, 1H), 7.16 (dt, J=7.7, 1.7 Hz, 1H), 7.24 (dt, *J*=7.6, 1.2 Hz, 1H), 7.40 (s, 1H), 7.55 (dd, *J*=8.0, 1.2 Hz, 1H); ^13^C NMR (100 MHz, CDCl_3_): δ 28.6, 53.5, 61.3, 121.8, 123.2, 128.0, 130.1, 130.2, 133.0, 134.1, 145.7.

**1-(1-benzyl-1*H*-1,2,3-triazol-4-yl)cyclopentanol** (**3af**): ^6^ White solid, (UV: 168mg, 69%; Daylight + Et_3_N: 92mg, 38%) mp: 71-72^o^C. Synthesized following the general procedure from azidomethylbenzene **1a** (133 mg, 1.0 mmol), 1-ethynylcyclopentanol **2f** (110 mg, 1.0 mmol), triethylamine (0.14 mL, 1 mmol). ^1^H NMR (400 MHz, CDCl_3_): δ 1.67-1.72 (m, 2H), 1.83-2.02 (m, 6H), 3.33 (s, 1H), 5.38 (s, 2H), 7.17-7.20 (m, 2H), 7.27-7.30 (m, 3H), 7.38 (s, 1H); ^13^C NMR (100 MHz, CDCl_3_): δ 23.4 (2C), 41.0 (2C), 53.9, 78.6, 119.8, 127.9 (2C), 128.5, 128.9 (2C), 134.5, 154.6.

**1-(1-(2-bromobenzyl)-1*H*-1,2,3-triazol-4-yl)cyclopentanol** (**3bf**): Colourless viscous mass, (UV: 248mg, 77%; Daylight + Et_3_N: 151mg, 47%). Synthesized following the general procedure from 1-(azidomethyl)-2-bromobenzene **1b** (212 mg, 1.0 mmol), 1-ethynylcyclopentanol **2f** (110 mg, 1.0 mmol), triethylamine (0.14 mL, 1 mmol. ^1^H NMR (400 MHz, CDCl_3_): δ 1.65-1.67 (m, 2H), 1.81-2.01 (m, 6H), 3.49 (s, 1H), 5.49 (s, 2H), 7.00 (dd, *J*=7.4, 1.4 Hz, 1H), 7.11 (dt, *J*=7.6, 1.2 Hz, 1H), 7.18 (dt, *J*=7.6, 1.2 Hz, 1H), 7.47 (s, 1H), 7.50 (dd, *J*=7.8, 1.2 Hz, 1H); ^13^C NMR (100 MHz, CDCl_3_): δ 23.2 (2C), 40.8 (2C), 53.3, 78.4, 120.0, 122.9, 127.8, 129.8, 129.9, 132.7, 133.9, 154.5.

**1-(1-benzyl-1*H*-1,2,3-triazol-4-yl)cyclohexanol** (**3ag**): ^6^ White solid, (UV: 216mg, 84%; Daylight + Et_3_N: 141mg, 55%) mp: 100-102^o^C. Synthesized following the general procedure from azidomethylbenzene **1a** (133 mg, 1.0 mmol), 1-ethynylcyclohexanol **2g** (124 mg, 1.0 mmol), triethylamine (0.14 mL, 1 mmol). ^1^H NMR (400 MHz, CDCl_3_): δ 1.25-1.29 (m, 1H), 1.43-1.54 (m, 3H), 1.64-1.69 (m, 2H), 1.78-1.89 (m, 4H), 2.99 (s, 1H), 5.42 (s, 2H), 7.19-7.21 (m, 2H), 7.28-7.31 (m, 3H), 7.35 (s, 1H); ^13^C NMR (100 MHz, CDCl_3_): δ 21.6 (2C), 25.1, 37.7 (2C), 53.8, 69.2, 119.4, 127.8 (2C), 128.4, 128.8 (2C), 134.4, 155.8.

**1-(1-(2-bromobenzyl)-1*H*-1,2,3-triazol-4-yl)cyclohexanol** (**3bg**): White solid, (UV: 205mg, 79%; Daylight + Et_3_N: 178mg, 53%) mp: 81-82^o^C. Synthesized following the general procedure from from 1-(azidomethyl)-2-bromobenzene **1b** (212 mg, 1.0 mmol), 1-ethynylcyclohexanol **2g** (124 mg, 1.0 mmol), triethylamine (0.14 mL, 1 mmol).  ^1^H NMR (400 MHz, CDCl_3_): δ 1.27-1.94 (m, 10H), 2.57 (s, 1H), 5.57 (s, 2H), 7.06 (dd, *J*=7.6, 1.6 Hz, 1H), 7.16 (dt, *J*=7.7, 1.7 Hz, 1H), 7.24 (dt, *J*=7.6, 1.2 Hz, 1H) 7.44 (s, 1H) 7.55 (dd, *J*=8.0, 1.2 Hz, 1H); ^13^C NMR (100 MHz, CDCl_3_): δ 21.7 (2C), 25.1, 37.8 (2C), 53.5, 69.3, 119.8, 123.1, 127.9, 129.9, 130.1, 132.9, 134.1, 155.8.

**4-((1-benzyl-1*H*-1,2,3-triazol-4-yl)methoxy)-6-methyl-2H-pyran-2-one** (**3ah**): White solid, (UV: 205mg, 69%; Daylight + Et_3_N: 134mg, 45%) mp: 133-134^o^C. Synthesized following the general procedure from azidomethylbenzene **1a** (133 mg, 1.0 mmol), 6-methyl-4-(prop-2-ynyloxy)-2H-pyran-2-one **2h** (164 mg, 1.0 mmol), triethylamine (0.14 mL, 1 mmol). ^1^H NMR (400 MHz, CDCl_3_): δ 2.08 (s, 3H), 5.02 (s, 2H), 5.44 (d, *J*= 2.0 Hz, 1H), 5.46 (s, 2H), 5.69 (s, 1H), 7.19-7.21 (m, 2H), 7.28-7.30 (m, 3H), 7.54 (s, 1H); ^13^C NMR (100 MHz, CDCl_3_): δ 19.7, 54.2, 61.9, 88.2, 100.2, 123.3, 128.0 (2C), 128.8, 129.1 (2C), 129.2, 134.1, 162.2, 164.5, 169.7.

**4-(4-methoxyphenyl)-1-phenyl-1H-1,2,3-triazole** (**3cb**): ^7^ White solid, (UV: 213mg, 85%; Daylight + Et_3_N: 153mg, 61%) mp: 143-144^o^C. Synthesized following the general procedure from azidobenzene **1c** (119 mg, 1.0 mmol), 1-ethynyl-4-methoxybenzene **2b** (132 mg, 1.0 mmol),, triethylamine (0.14 mL, 1 mmol). ^1^H NMR (400 MHz, CDCl_3_): δ 3.82 (s, 3H), 6.96 (d, *J*=8.8 Hz, 2H), 7.39-7.43 (m, 1H), 7.50 (dt, *J*=6.8, 1.3 Hz, 2H), 7.74-7.76 (m, 2H), 7.79-81 (m, 2H), 8.08 (s, 1H); ^13^C NMR (100 MHz, CDCl_3_): δ 55.3, 114.2 (2C), 116.7, 120.4 (2C), 122.8, 127.1 (2C), 128.6, 129.7 (2C), 137.0, 148.2, 159.7.

**4-(naphthalen-2-yl)-1-phenyl-1H-1,2,3-triazole** (**3da**): White solid, (UV: 238mg, 88%; Daylight + Et_3_N: 171mg, 63%) mp: 190-191^o^C. Synthesized following the general procedure from 2-azidonaphthalene **1d** (169 mg, 1.0 mmol), phnylacetelene **2a** (102 mg, 1.0 mmol), triethylamine (0.14 mL, 1 mmol). ^1^H NMR (400 MHz, CDCl_3_): δ 7.35-7.37 (m, 1H), 7.45-7.51 (m, 2H), 7.48-7.63 (m, 4H), 7.69 (d, *J*=8.4, 1H), 7.96 (dd, *J*=7.2, 1.6 3H), 8.02 (d, *J*=8.0 Hz, 1H), 8.13 (s, 1H); ^13^C NMR (100 MHz, CDCl_3_): δ 122.2, 122.3, 123.5, 124.9, 125.8 (2C),127.0, 127.8, 128.2, 128.3, 128.5, 128.9 (2C), 130.2, 130.4, 133.7, 134.1, 147.

**4-Phenyl-1-(2,3,4,6-tetra-*O*-acetyl-β-D-glucopyranosyl)-1*H*-1,2,3-triazole** (**3ea**):^8^ White solid, (UV: 352 mg, 74%; Daylight + Et_3_N: 244 mg, 52%) mp: 211-212^o^C. Synthesized following the general procedure from 1-azido-2,3,4,6-tetra-*O*-acetyl-β-D-glucopyranose **1e** (373 mg, 1.0 mmol), phnylacetelene **2a** (102 mg, 1.0 mmol). ^1^H NMR (400 MHz, CDCl_3_): δ1.87 (s, 3H), 1.99 (s, 3H), 2.02 (s, 3H), 2.22 (s, 3H), 4.13-4.18 (m, 2H), 4.20-4.26 (m, 1H), 5.26 (dd, *J*= 10.2, 3.4 Hz, 1H), 5.55 (d, *J*= 2.8 Hz, 1H), 5.61 (t, *J*= 9.8 Hz, 1H), 5.88 (d, *J*= 9.2 Hz, 1H), 7.30-7.34 (m, 1H), 7.38-7.42 (m, 2H), 7.81-7.84 (m, 2H), 8.03 (s, 1H); ^13^C NMR (100 MHz, CDCl_3_): δ 20.2, 20.5, 20.5, 20.6, 61.8, 66.9, 67.7, 70.8, 74.0, 86.3, 117.8, 125.9 (2C), 128.5, 128.8 (2C), 129.9, 148.4, 167.1, 169.7, 169.9, 170.3.

**4-Phenyl-1-(2,3,4,6-tetra-*O*-acetyl-β-D-galactopyranosyl)-1*H*-1,2,3-triazole** (**3fa**): White solid, (UV: 333 mg, 70%; Daylight + Et_3_N: 228 mg, 48%) mp: 208-209^o^C. Synthesized following the general procedure from 1-azido-2,3,4,6-tetra-*O*-acetyl-β-D-galactopyranose **1f** (373 mg, 1.0 mmol), phnylacetelene **2a** (102 mg, 1.0 mmol).^1^H NMR (400 MHz, CDCl_3_): δ1.87 (s, 3H), 1.99 (s, 3H), 2.22 (s, 3H), 2.22 (s, 3H), 3.82-4.01 (m, 1H), 4.20-4.23 (m, 2H), 5.24 (dd, *J*= 10.2, 3.4 Hz, 1H), 5.54 (d, *J*= 3.2, 1H), 5.61 (t, *J*= 9.8 Hz, 1H), 5.86 (d, *J*= 9.6 Hz, 1H), 6.94 (d, *J*= 8.8 Hz, 3H), 7.75 (d, *J*= 8.8 Hz, 2H), 7.93 (s, 1H); ^13^C NMR (100 MHz, CDCl_3_): δ 20.3, 20.5, 20.6, 20.7, 61.2, 66.9, 67.7, 70.8, 74.0, 86.3, 114.2 (2C), 116.9, 122.7, 127.2 (2C), 133.4, 148.3, 169.1, 169.8, 169.9, 170.3.

**4-p-tolyl-1-(2,3,4,6-tetra-*O*-acetyl-β-D--glucopyranosyl)-1*H*-1,2,3-triazole** (**3ei**): White solid, (UV: 357 mg, 73%; Daylight + Et_3_N: 270 mg, 55%) mp: 198-199^o^C. Synthesized following the general procedure from 1-azido-2,3,4,6-tetra-*O*-acetyl-β-D-glucopyranose **1e** (373 mg, 1.0 mmol), 1-ethynyl-4-methylbenzene **2i** (116 mg, 1.0 mmol). ^1^H NMR (400 MHz, CDCl_3_): δ1.85 (s, 3H), 2.00 (s, 3H), 2.05 (s, 3H), 2.06 (s, 3H), 2.35 (s, 3H), 3.98-4.03 (m, 1H) 4.13 (dd, *J*= 12.4, 1.6 Hz, 1H), 4.30 (dd, *J*= 12.6, 5.0 Hz, 1H), 5.24 (t, *J*= 9.6 Hz, 1H), 5.43 (t, *J*= 9.6 Hz, 1H), 5.47 (t, *J*= 9.6 Hz, 1H), 5.91 (d, *J*= 8.0 Hz, 1H) 7.21 (d, *J*= 7.6 Hz, 2H), 7.70 (d, *J*= 8.0Hz, 2H), 7.93 (s, 1H); ^13^C NMR (100 MHz, CDCl_3_): δ 20.1 (2C), 20.5, 20.6, 21.3, 61.6, 67.8, 70.2, 72.8, 75.1, 85.7, 117.3, 125.8 (2C), 127.1, 129.5 (2C), 138.4, 148.5, 168.9, 169.3, 169.8, 170.4.

**4-p-tolyl-1-(2,3,4,6-tetra-*O*-acetyl-β-D-galactopyranosyl)-1*H*-1,2,3-triazole** (**3fi**): White solid, (UV: 337 mg, 69%; Daylight + Et_3_N: 205 mg, 42%) mp: 200-201^o^C. Synthesized following the general procedure from 1-azido-2,3,4,6-tetra-*O*-acetyl-β-D-galactopyranose **1f** (373 mg, 1.0 mmol), 1-ethynyl-4-methylbenzene **2i** (116 mg, 1.0 mmol). ^1^H NMR (400 MHz, CDCl_3_): δ1.84 (s, 3H), 1.97 (s, 3H), 1.99 (s, 3H), 2.19 (s, 3H), 2.33 (s, 3H), 4.09-4.19 (m, 2H) 4.22-4.25 (m, 1H), 5.25 (dd, *J*= 10.2, 3.4 Hz, 1H), 5.52 (d, *J*= 2.8 Hz, 1H), 5.60 (t, *J*= 9.8 Hz, 1H), 5.88 (d, *J*= 9.2 Hz, 1H), 7.19 (d, *J*= 8.0 Hz, 2H), 7.70 (d, *J*= 8.0Hz, 2H), 7.98 (s, 1H); ^13^C NMR (100 MHz, CDCl_3_): δ 20.1(2C), 20.4, 20.4, 21.5, 61.1, 66.9, 67.7, 70.8, 73.9, 86.1, 117.4, 125.7 (2C), 127.1, 129.4 (2C), 138.2, 148.3, 169.0, 169.7, 169.9, 170.2.

**Methyl 1-benzyl-1*H*-1,2,3-triazole-4-carboxylate** (**5aj**): ^1^ White solid, (126mg, 58%) mp: 113-115^o^C. Synthesized following the general procedure from benzylbromide **4a** (171 mg, 1.0 mmol), NaN_3_(78 mg, 1.2 mmol), methyl propiolate **2j** (84 mg, 1.0 mmol). ^1^H NMR (400 MHz, CDCl_3_): δ 3.79(s, 3H), 5.49 (s, 2H), 7.18-7.20 (m, 2H), 7.24-7.28 (m, 3H), 7.99 (s, 1H); ^13^C NMR (100 MHz, CDCl_3_): δ 51.8, 54.1, 127.3, 127.9 (2C), 128.7, 128.9 (2C), 133.6, 139.8, 160.7.

**Methyl 1-(2-bromobenzyl)-1*H*-1,2,3-triazole-4-carboxylate** (**5bj**): ^1^ White solid, (160 mg, 54%) mp: 120-122^o^C. Synthesized following the general procedure from *o*-bromobenzylbromide **4b** (249 mg, 1.0 mmol), NaN_3_(78 mg, 1.2 mmol), methyl propiolate **2j** (84 mg, 1.0 mmol). ^1^H NMR (400 MHz, CDCl_3_): δ 3.87 (s, 3H), 5.66 (s, 2H), 7.19-7.23 (m, 2H), 7.26-7.31 (m, 1H), 7.58 (d, *J*=8.0 Hz, 1H), 8.05 (s, 1H); ^13^C NMR (100 MHz, CDCl_3_): δ 52.1, 54.1, 123.7, 127.6, 128.3, 130.7, 130.8, 133.1, 133.3, 140.9, 161.9.

**Ethyl 1-benzyl-1*H*-1,2,3-triazole-4-carboxylate** (**5ak**): ^1^ Pale yellow solid, (141mg, 61%) mp: 92-94^o^C. Synthesized following the general procedure from *o*-bromobenzylbromide **4b** (249 mg, 1.0 mmol), NaN_3_(78 mg, 1.2 mmol), ethyl propiolate **2k** (98 mg, 1.0 mmol). ^1^H NMR (400 MHz, CDCl_3_): δ 1.32 (t, *J* = 7.0 Hz, 3H), 4.33 (q, *J* = 7.2 Hz, 2H), 5.53 (s, 2H), 7.22-7.25(m, 2H), 7.31-7.33 (m, 3H), 7.96 (s, 1H); ^13^C NMR (100 MHz, CDCl_3_): δ 14.1, 54.3, 61.1, 127.3, 128.1 (2C), 128.9, 129.1 (2C), 133.7, 140.4, 160.5.

**1-benzyl-4-propyl-1H-1,2,3-triazole** (**5al**): ^5^ Light yellow liquid, (131mg, 65%). Synthesized following the general procedure from benzylbromide **4a** (171 mg, 1.0 mmol), NaN_3_(65 mg, 1.0 mmol), methyl pent-1-yne **2l** (68 mg, 1.0 mmol), triethylamine (0.14 mL, 1 mmol). ^1^H NMR (400 MHz, CDCl_3_): δ 0.91(t, *J* = 7.2 Hz, 3H), 1.63 (q, *J* = 7.6 Hz, 2H,), 2.63 (t, *J* = 7.2 Hz, 2H), 5.44 (s, 2H), 7.20-7.24 (m, 3H), 7.28-7.31(m, 3H); ^13^C NMR (100 MHz, CDCl_3_): δ 13.5, 22.3, 27.4, 53.6, 120.5, 127.6 (2C), 128.2, 128.7(2C), 134.8, 148.3.

**1-Benzyl-4-butyl-1*H*-1,2,3-triazole** (**5am**): ^5^ Yellow liquid, (135mg, 63%). Synthesized following the general procedure from azidomethylbenzene **4a** (133 mg, 1.0 mmol), hex-1-yne **2m** (82 mg, 1.0 mmol), triethylamine (0.14 mL, 1 mmol). ^1^H NMR (400 MHz, CDCl_3_): δ 0.81 (t, *J* = 7.2 Hz, 3H), 1.26 (sex, *J*=7.2 Hz, 2H), 1.53 (pent, *J*= 7.6 Hz, 2H), 2.62 (t, *J* = 7.8 Hz, 2H), 5.37 (s, 2H), 7.12-7.16 (m, 3H), 7.21-7.25 (m, 3H); ^13^C NMR (100 MHz, CDCl_3_): δ 13.5, 21.9, 25.0, 31.2, 53.5, 120.4, 127.5 (2C), 128.2, 128.6 (2C), 134.8, 148.4.

Experimental references:

1. (a) R. U. Islam, A. Taher, M. Choudhary, S. Siwal, K. Mallick, *Sci*. *Rep*., **2015**, *5.* 9632. (b) R. [Islam, A. U](http://0-fl-www.reaxys.com.ujlink.uj.ac.za/reaxys/secured/paging.do?performed=true&action=restore). [Taher,](http://0-fl-www.reaxys.com.ujlink.uj.ac.za/reaxys/secured/paging.do?performed=true&action=restore) M. [Choudhary,](http://0-fl-www.reaxys.com.ujlink.uj.ac.za/reaxys/secured/paging.do?performed=true&action=restore) J. M.  [Witcomb,](http://0-fl-www.reaxys.com.ujlink.uj.ac.za/reaxys/secured/paging.do?performed=true&action=restore) K.  [Mallick,](http://0-fl-www.reaxys.com.ujlink.uj.ac.za/reaxys/secured/paging.do?performed=true&action=restore) 
   *Dalton Trans.*, **2014**, *44*, 1341 – 1349. (c) A. Taher, D. Nandi, R. U. Islam, M. Choudhary, K. Mallick, *RSC Advances*, **2015**, *5*, 47275-47283.
2. S. [Proietti, A. Ilaria](http://0-fl-www.reaxys.com.ujlink.uj.ac.za/reaxys/secured/paging.do?performed=true&action=restore), G. N. [Khairallah,](http://0-fl-www.reaxys.com.ujlink.uj.ac.za/reaxys/secured/paging.do?performed=true&action=restore)  S. Y. [Wan,](http://0-fl-www.reaxys.com.ujlink.uj.ac.za/reaxys/secured/paging.do?performed=true&action=restore) T. [Quach,](http://0-fl-www.reaxys.com.ujlink.uj.ac.za/reaxys/secured/paging.do?performed=true&action=restore) S. [Tsegay,](http://0-fl-www.reaxys.com.ujlink.uj.ac.za/reaxys/secured/paging.do?performed=true&action=restore) C. M. [Williams,](http://0-fl-www.reaxys.com.ujlink.uj.ac.za/reaxys/secured/paging.do?performed=true&action=restore) R. A. J. [O'Hair,](http://0-fl-www.reaxys.com.ujlink.uj.ac.za/reaxys/secured/paging.do?performed=true&action=restore) [Donnelly, S. Paul](http://0-fl-www.reaxys.com.ujlink.uj.ac.za/reaxys/secured/paging.do?performed=true&action=restore), S. J.  [Williams,](http://0-fl-www.reaxys.com.ujlink.uj.ac.za/reaxys/secured/paging.do?performed=true&action=restore) *Org*. *Biomol*. *Chem*., **2011**, *9*, 6082 – 6088.
3. G. [Colombano,](http://0-fl-www.reaxys.com.ujlink.uj.ac.za/reaxys/secured/paging.do?performed=true&action=restore) C. [Albani,](http://0-fl-www.reaxys.com.ujlink.uj.ac.za/reaxys/secured/paging.do?performed=true&action=restore) G. [Ottonello,](http://0-fl-www.reaxys.com.ujlink.uj.ac.za/reaxys/secured/paging.do?performed=true&action=restore) A.  [Ribeiro,](http://0-fl-www.reaxys.com.ujlink.uj.ac.za/reaxys/secured/paging.do?performed=true&action=restore) R.  [Scarpelli,](http://0-fl-www.reaxys.com.ujlink.uj.ac.za/reaxys/secured/paging.do?performed=true&action=restore) G.  [Tarozzo,](http://0-fl-www.reaxys.com.ujlink.uj.ac.za/reaxys/secured/paging.do?performed=true&action=restore) J. [Daglian,](http://0-fl-www.reaxys.com.ujlink.uj.ac.za/reaxys/secured/paging.do?performed=true&action=restore) K-M. [Jung,](http://0-fl-www.reaxys.com.ujlink.uj.ac.za/reaxys/secured/paging.do?performed=true&action=restore) D. [Piomelli,](http://0-fl-www.reaxys.com.ujlink.uj.ac.za/reaxys/secured/paging.do?performed=true&action=restore) T. [Bandiera,](http://0-fl-www.reaxys.com.ujlink.uj.ac.za/reaxys/secured/paging.do?performed=true&action=restore) *ChemMedChem*, **2015**, *10*, 380 – 395.
4. C. [Deraedt,](http://0-fl-www.reaxys.com.ujlink.uj.ac.za/reaxys/secured/paging.do?performed=true&action=restore) N. [Pinaud,](http://0-fl-www.reaxys.com.ujlink.uj.ac.za/reaxys/secured/paging.do?performed=true&action=restore) D. [Astruc,](http://0-fl-www.reaxys.com.ujlink.uj.ac.za/reaxys/secured/paging.do?performed=true&action=restore) *J*. *Am*. *Chem*. *Soc*., **2014**, *136*, 12092 – 12098.
5. B. [Movassagh,](http://0-fl-www.reaxys.com.ujlink.uj.ac.za/reaxys/secured/paging.do?performed=true&action=restore) N.  [Rezaei,](http://0-fl-www.reaxys.com.ujlink.uj.ac.za/reaxys/secured/paging.do?performed=true&action=restore) *Tetrahedron*, **2014**, *70*, 8885 – 8892.
6. J. I. [Sarmiento-Sanchez,](http://0-fl-www.reaxys.com.ujlink.uj.ac.za/reaxys/secured/paging.do?performed=true&action=restore) A. [Ochoa-Teran,](http://0-fl-www.reaxys.com.ujlink.uj.ac.za/reaxys/secured/paging.do?performed=true&action=restore) I. A. [Rivero,](http://0-fl-www.reaxys.com.ujlink.uj.ac.za/reaxys/secured/paging.do?performed=true&action=restore) *Arkivoc*, **2011**, *9*, 177 – 188.
7. Z. [Chen,](http://0-fl-www.reaxys.com.ujlink.uj.ac.za/reaxys/secured/paging.do?performed=true&action=restore) Q. [Yan,](http://0-fl-www.reaxys.com.ujlink.uj.ac.za/reaxys/secured/paging.do?performed=true&action=restore) Z. [Liu,](http://0-fl-www.reaxys.com.ujlink.uj.ac.za/reaxys/secured/paging.do?performed=true&action=restore) Y.  [Zhang,](http://0-fl-www.reaxys.com.ujlink.uj.ac.za/reaxys/secured/paging.do?performed=true&action=restore) *Chem*. *Eur*. *J*., **2014**, *20*, 17635 – 17639.
8. D. Goyard, A. S. Chajistamatiou, A. I.  Sotiropoulou, E. D. Chrysina, J-P Praly, S. Vidal, *Chem*. *Eur*. *J*., **2014**, 20, 5423 – 5432.


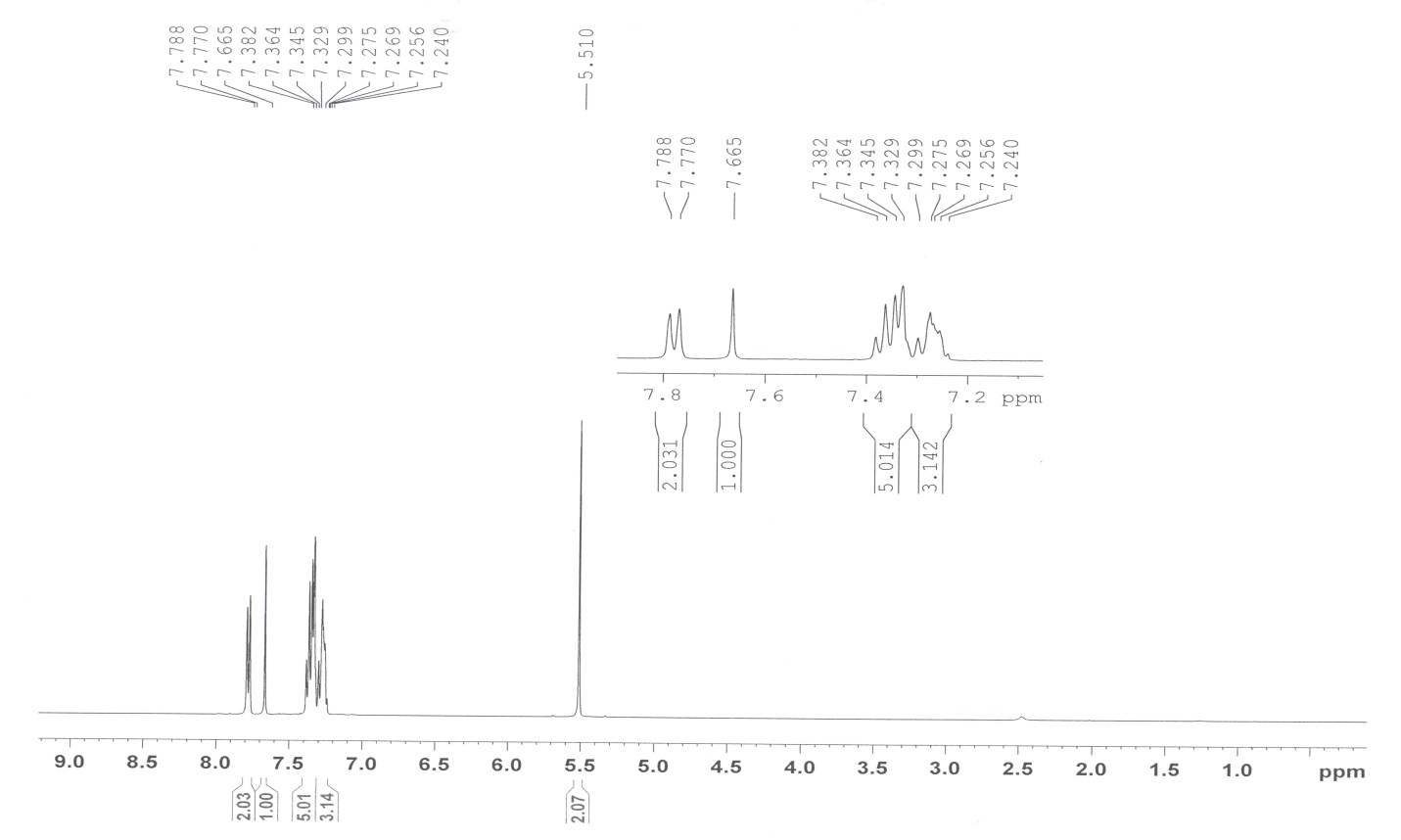


**Figure S1**. ^1^H NMR spectrum of **3aa** in CDCl_3_


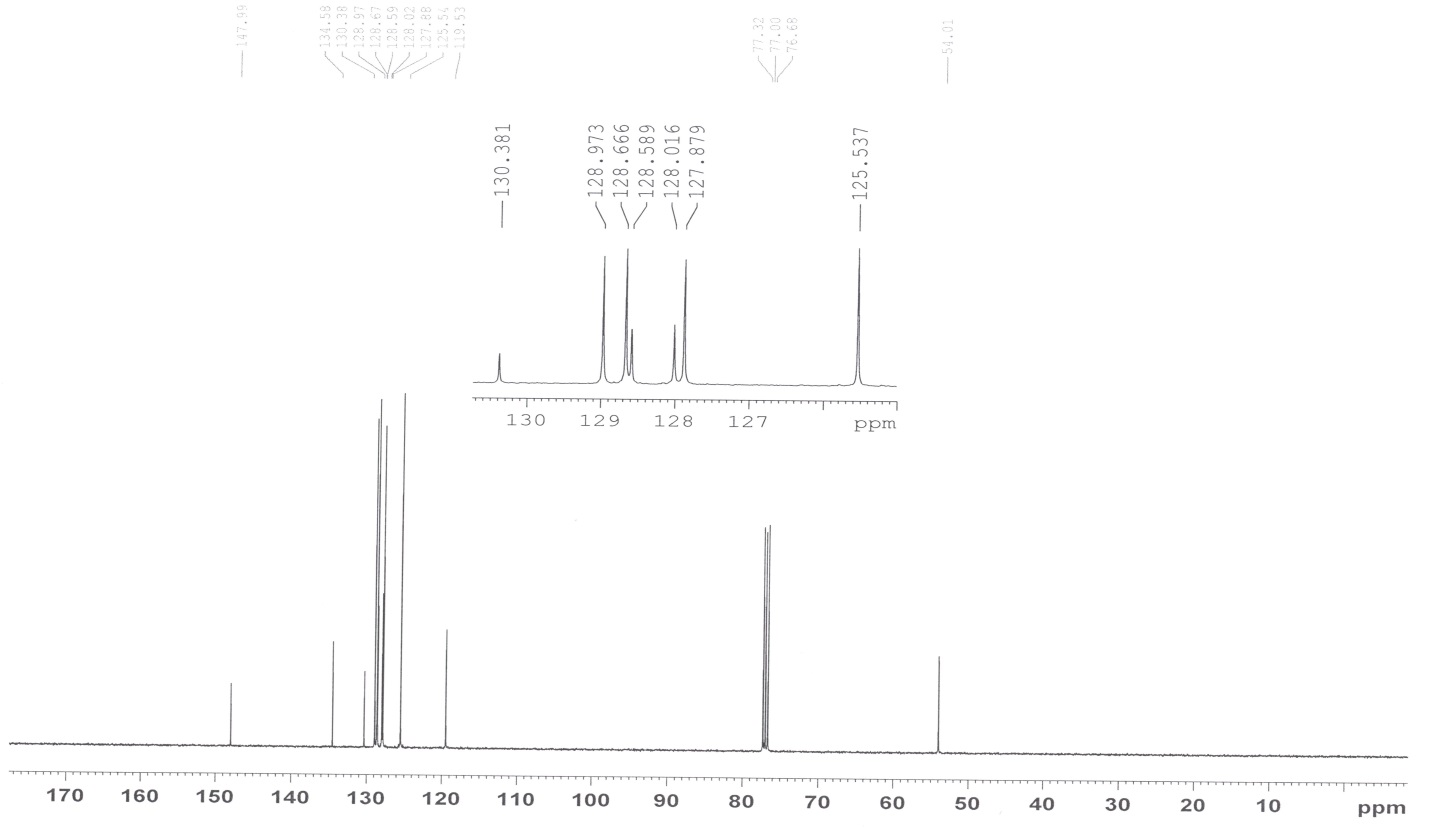


**Figure S2**. ^13^C NMR spectrum of **3aa** in CDCl_3_


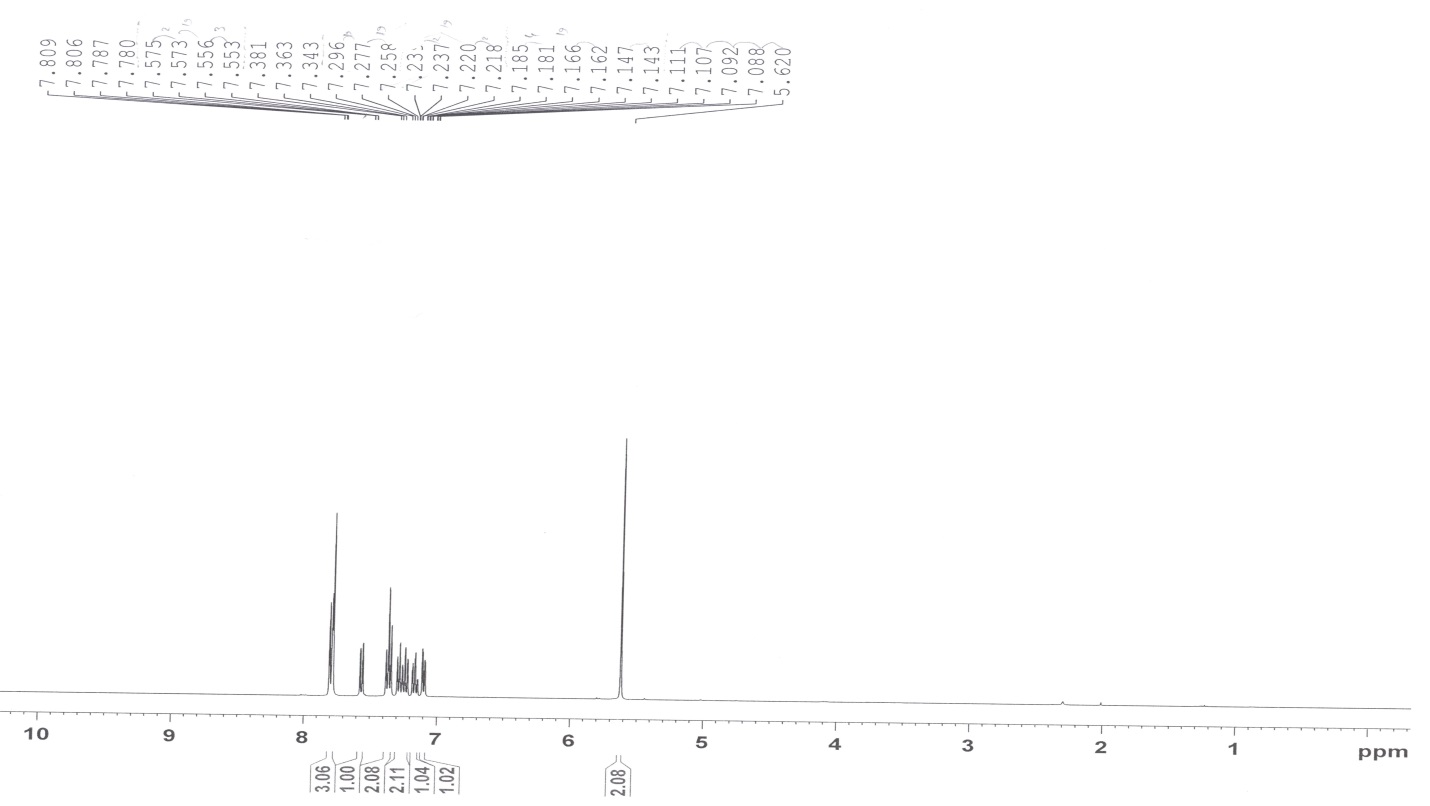


**Figure S3**. ^1^H NMR spectrum of **3ba** in CDCl_3_


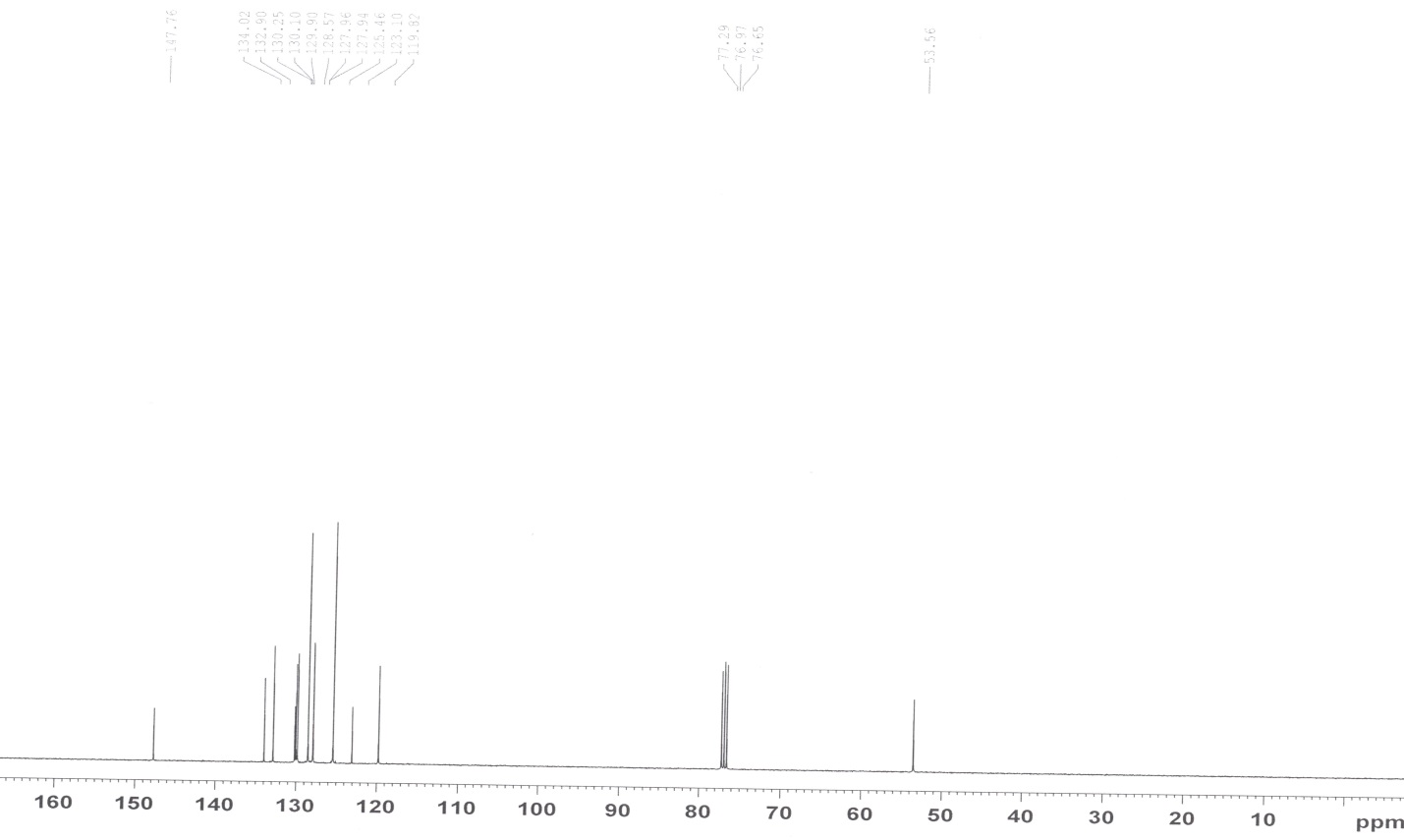


**Figure S4**. ^13^C NMR spectrum of **3ba** in CDCl_3_


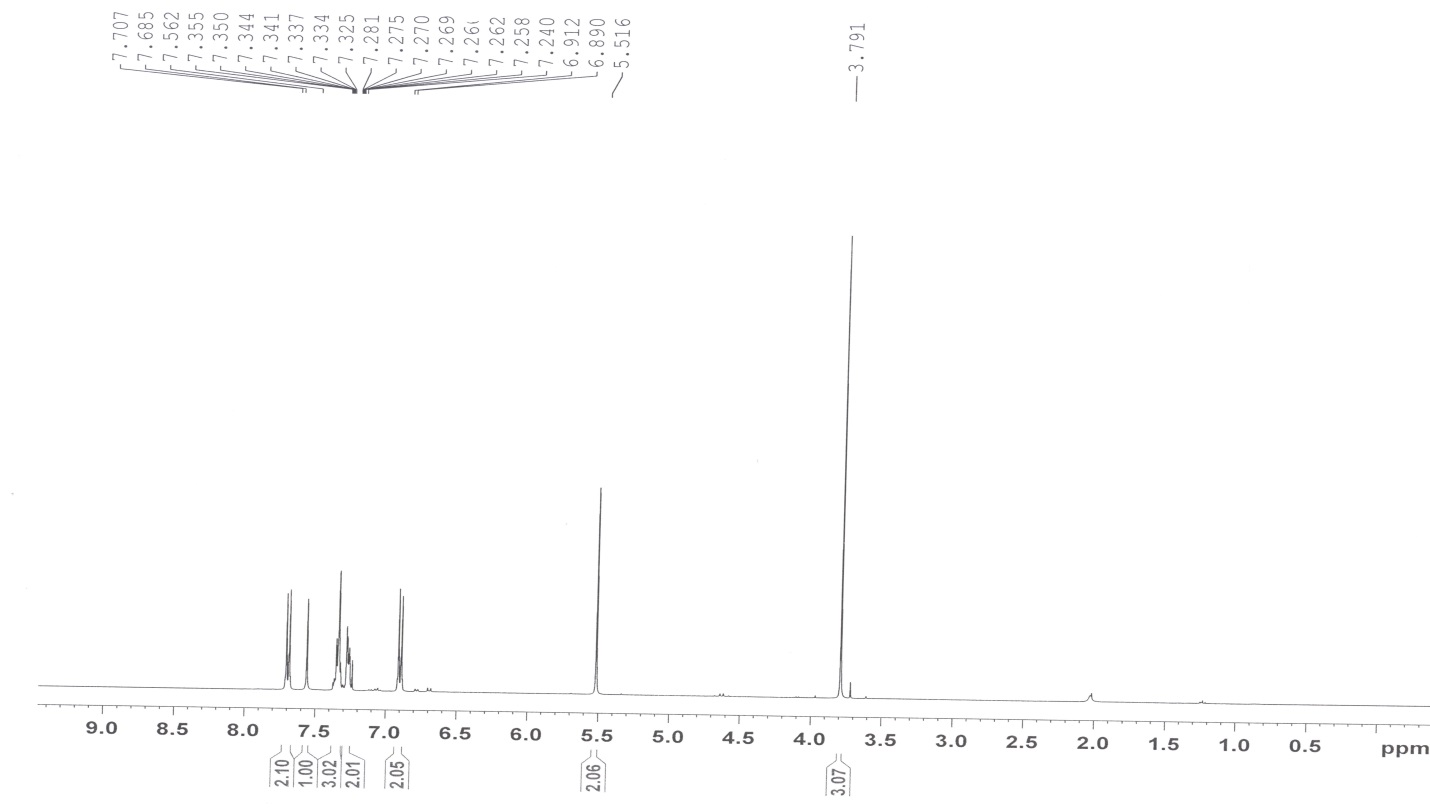


**Figure S5**. ^1^H NMR spectrum of **3ab** in CDCl_3_


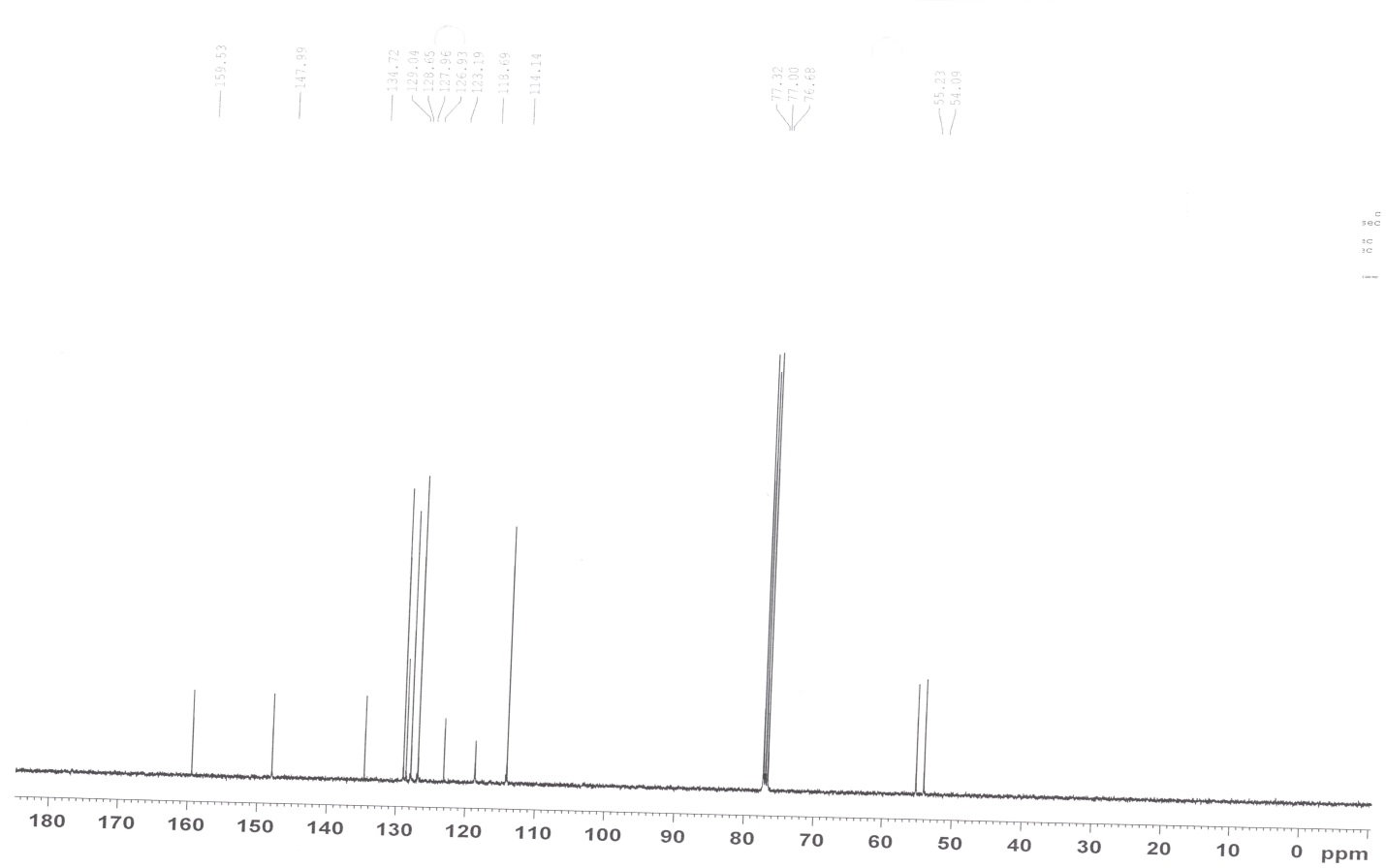


**Figure S6**. ^13^C NMR spectrum of **3ab** in CDCl_3_


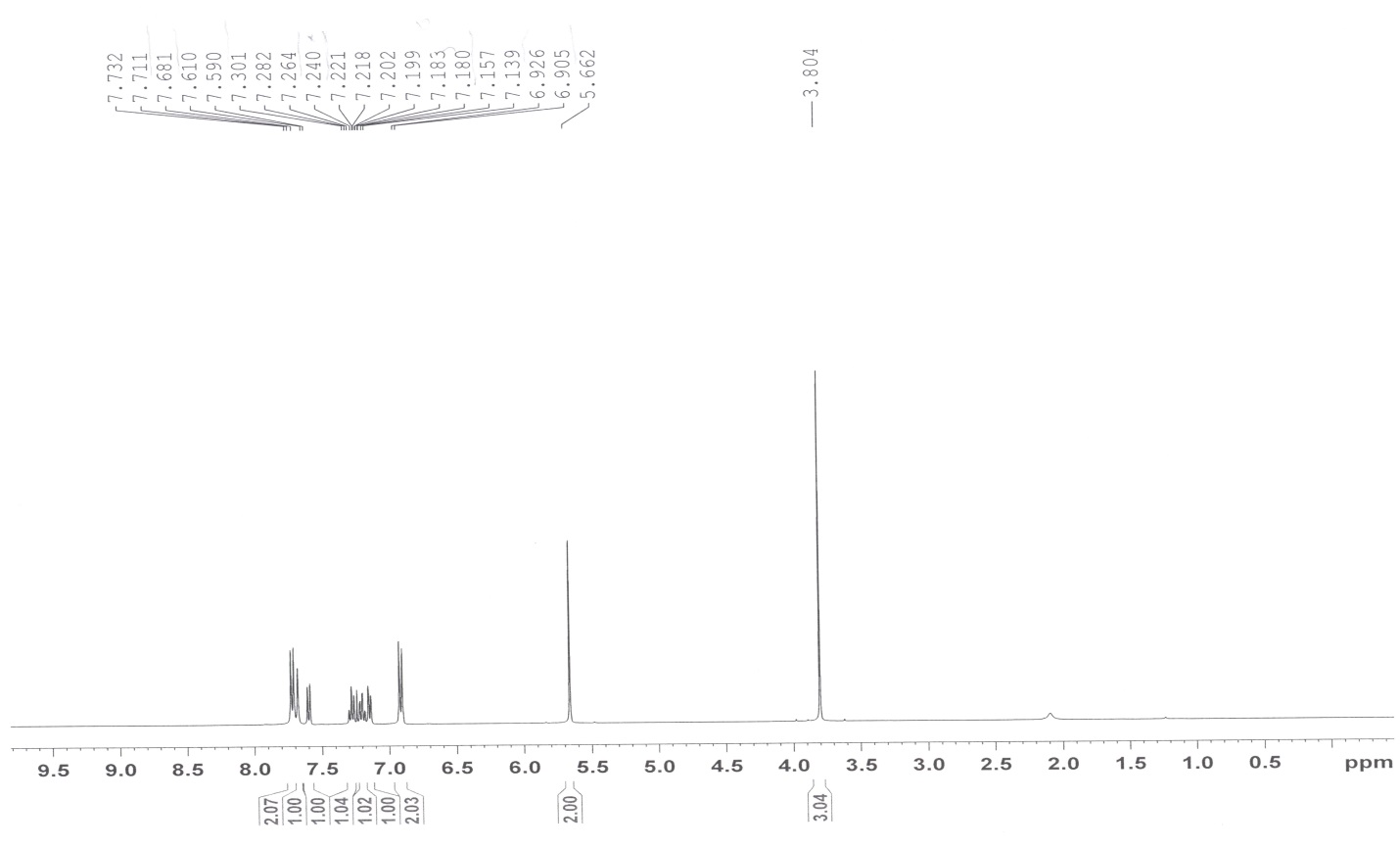


**Figure S7**. ^1^H NMR spectrum of **3bb** in CDCl_3_


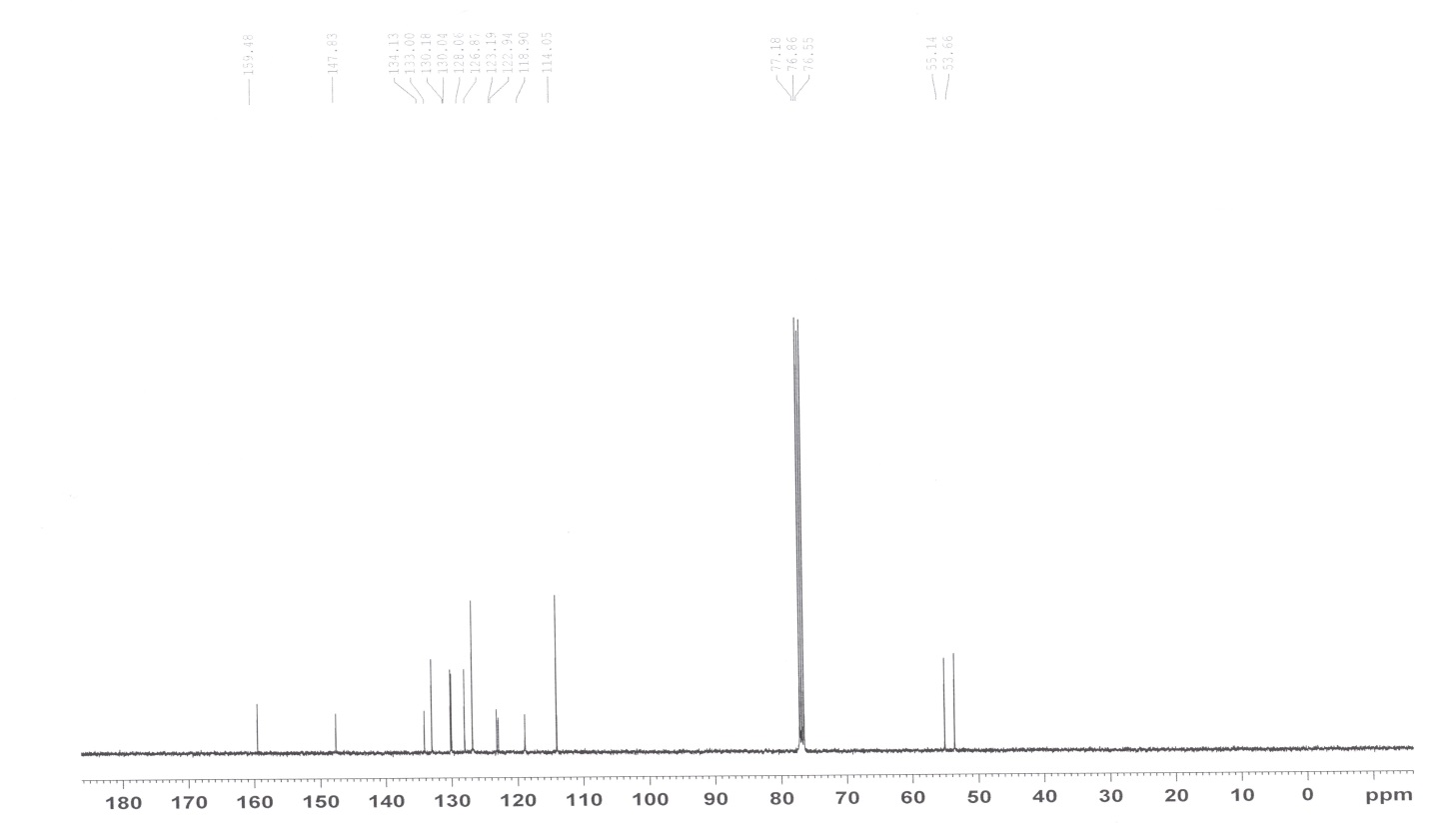


**Figure S8**. ^13^C NMR spectrum of **3bb** in CDCl_3_


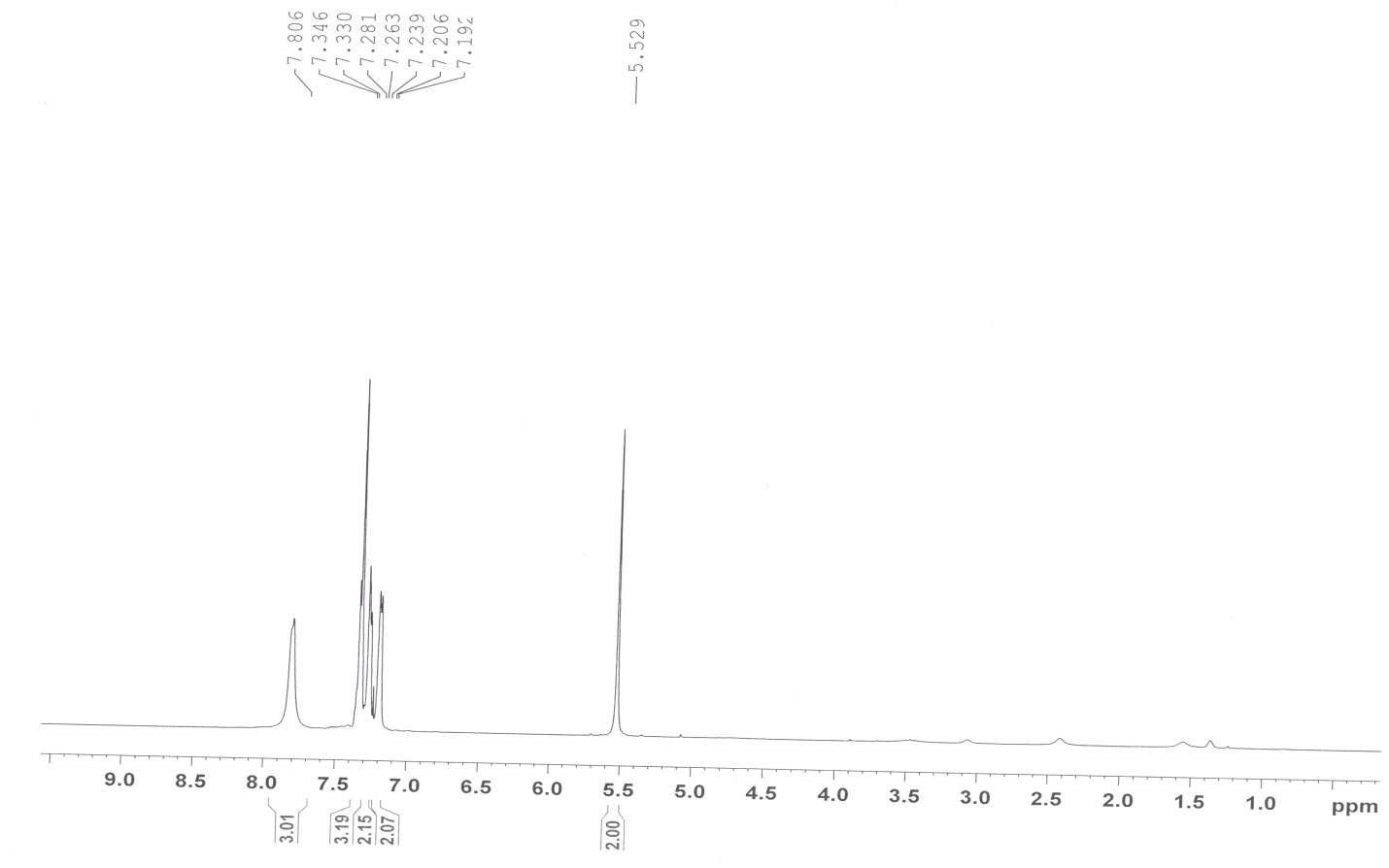


**Figure S9**. ^1^H NMR spectrum of **3ac** in CDCl_3_


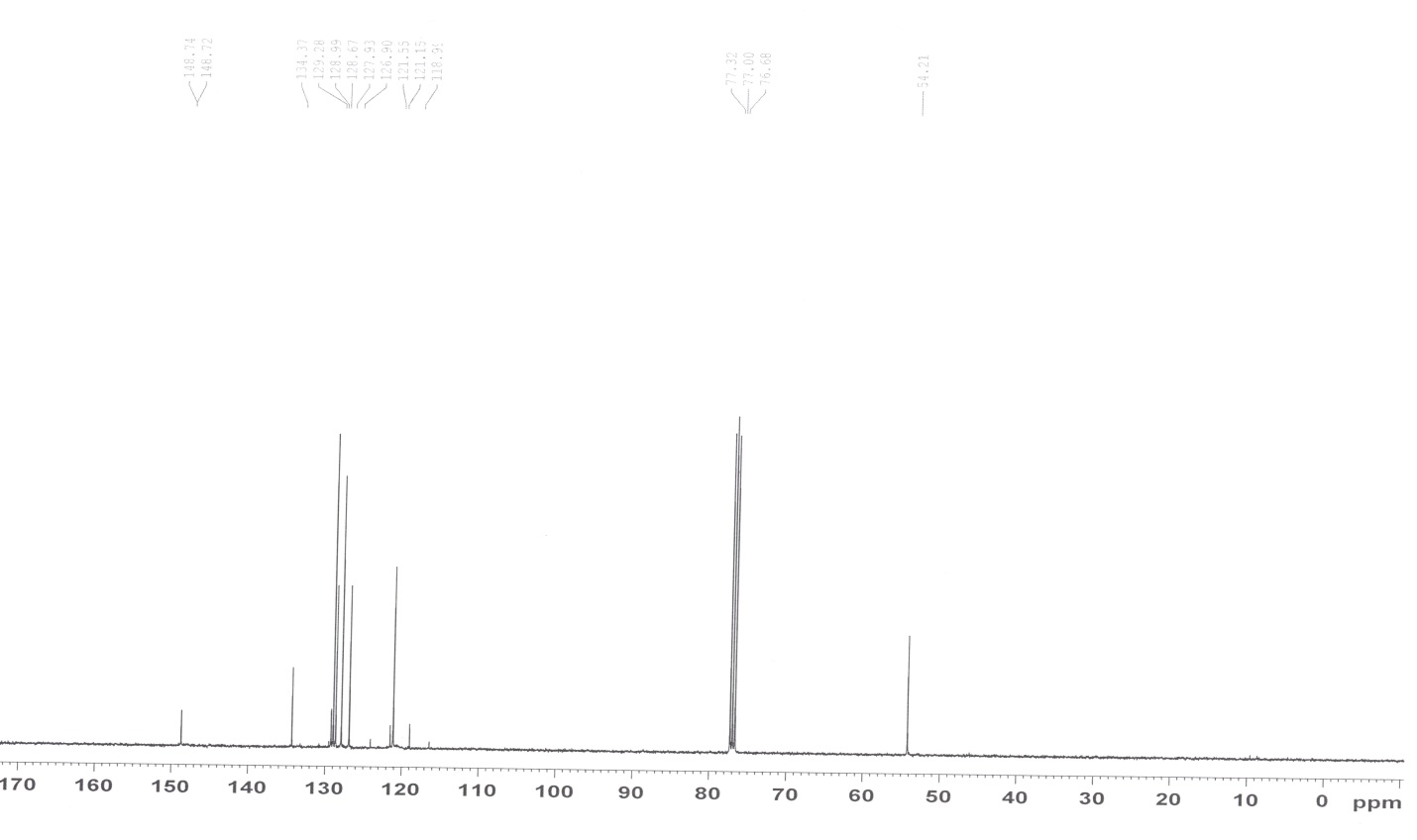


**Figure S10**. ^13^C NMR spectrum of **3ac** in CDCl_3_


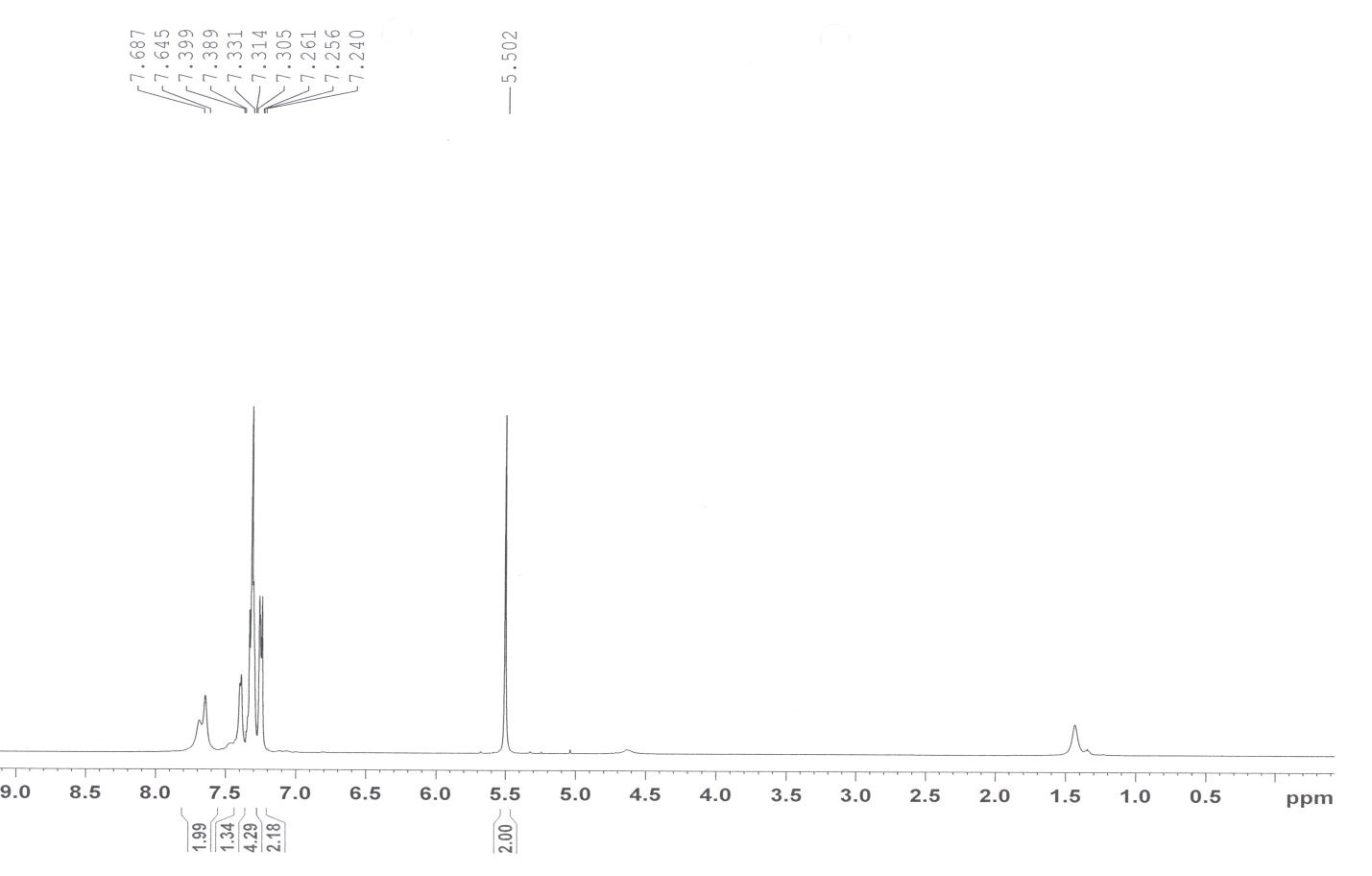


**Figure S11**. ^1^H NMR spectrum of **3ad** in CDCl_3_


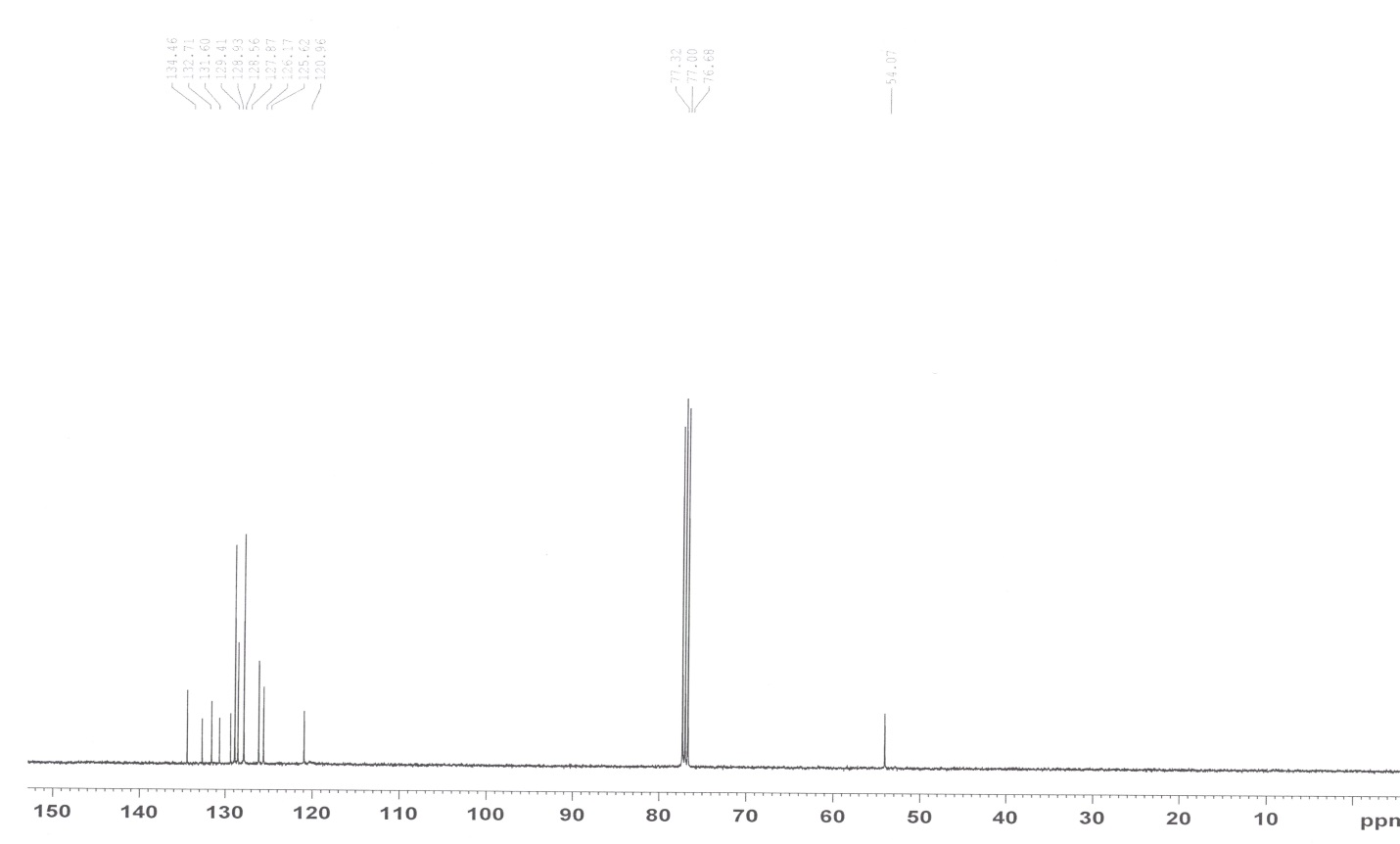
 **Figure S12**. ^13^C NMR spectrum of **3ad** in CDCl_3_


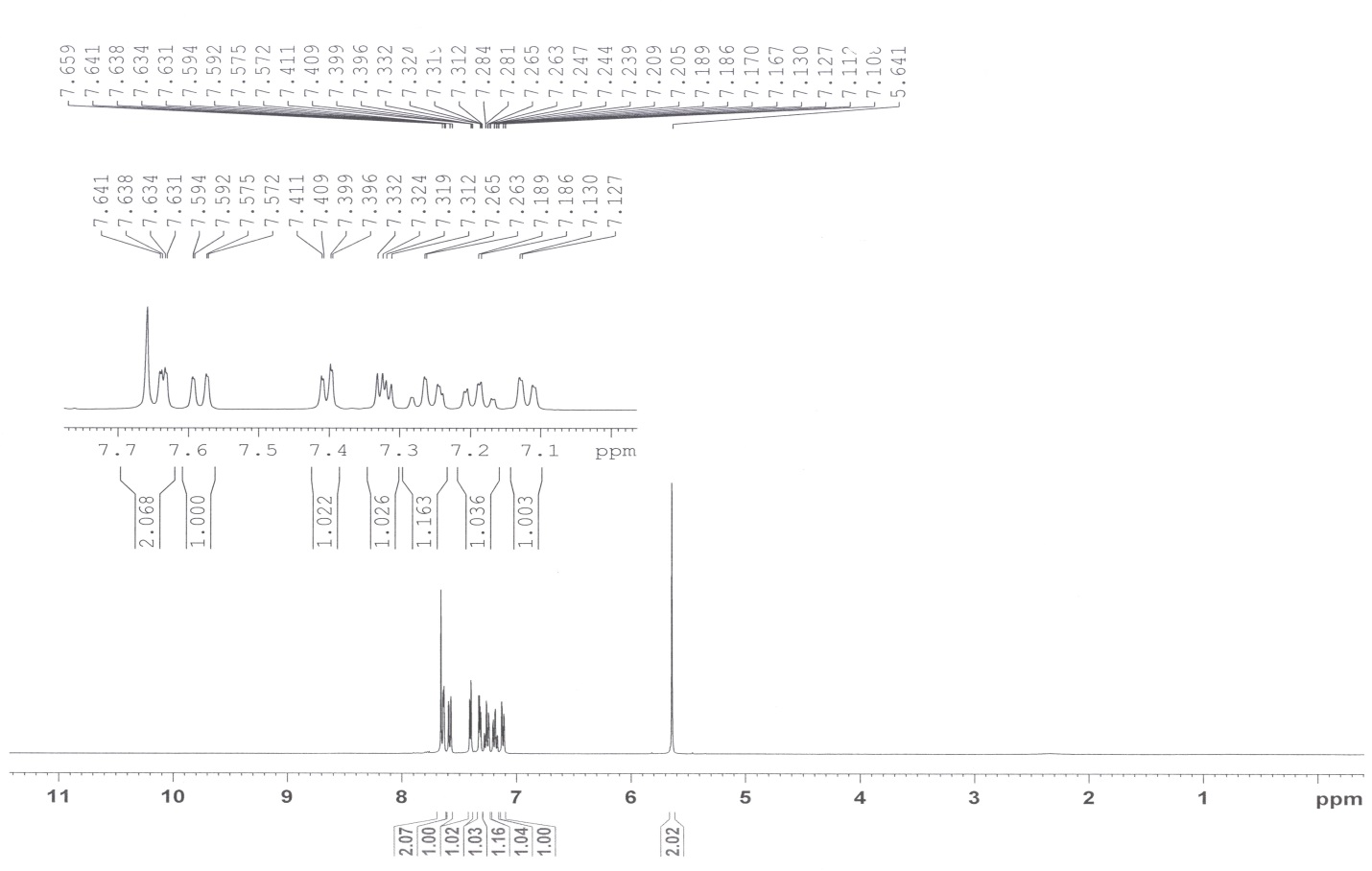


**Figure S13**. ^1^H NMR spectrum of **3bd** in CDCl_3_


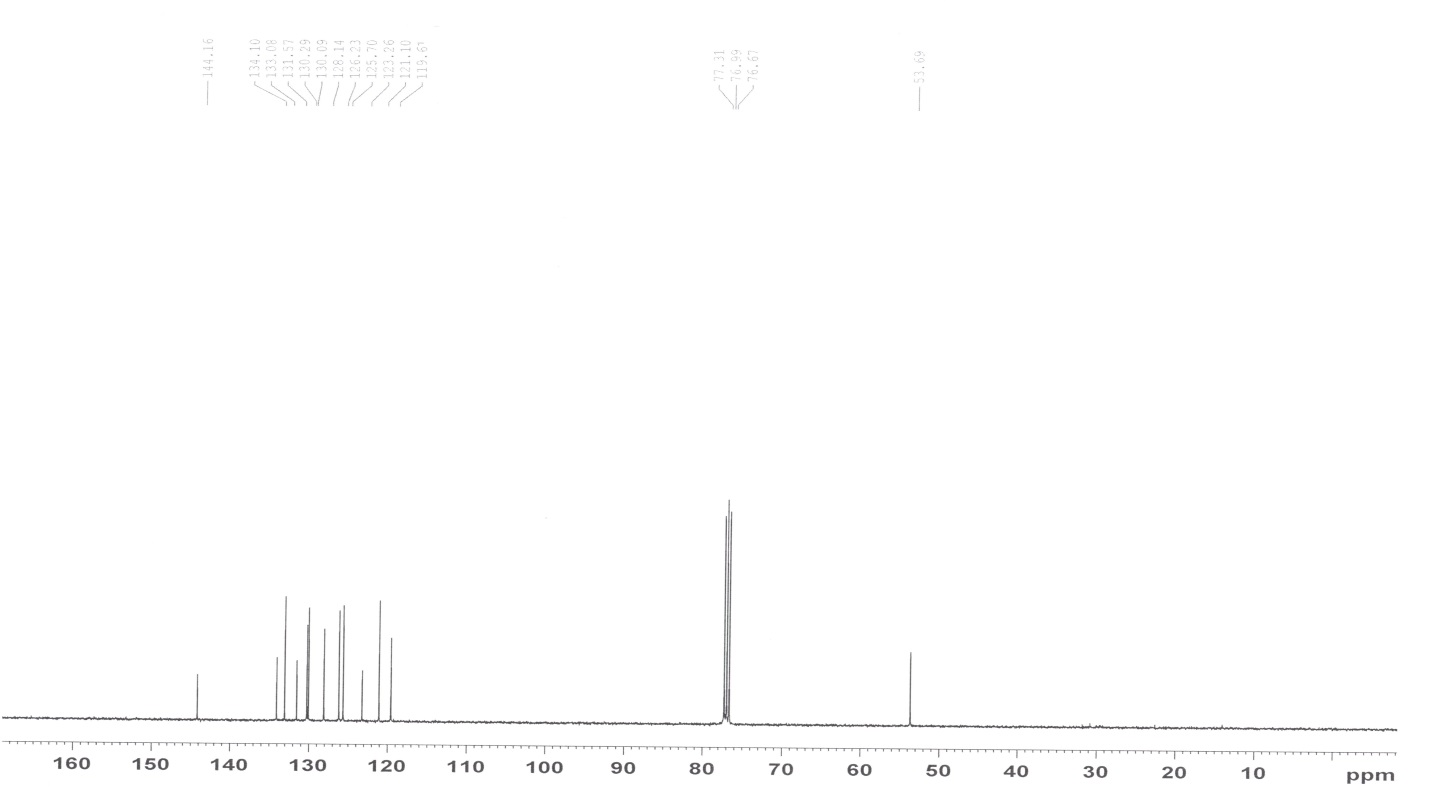


**Figure S14**. ^13^C NMR spectrum of **3bd** in CDCl_3_


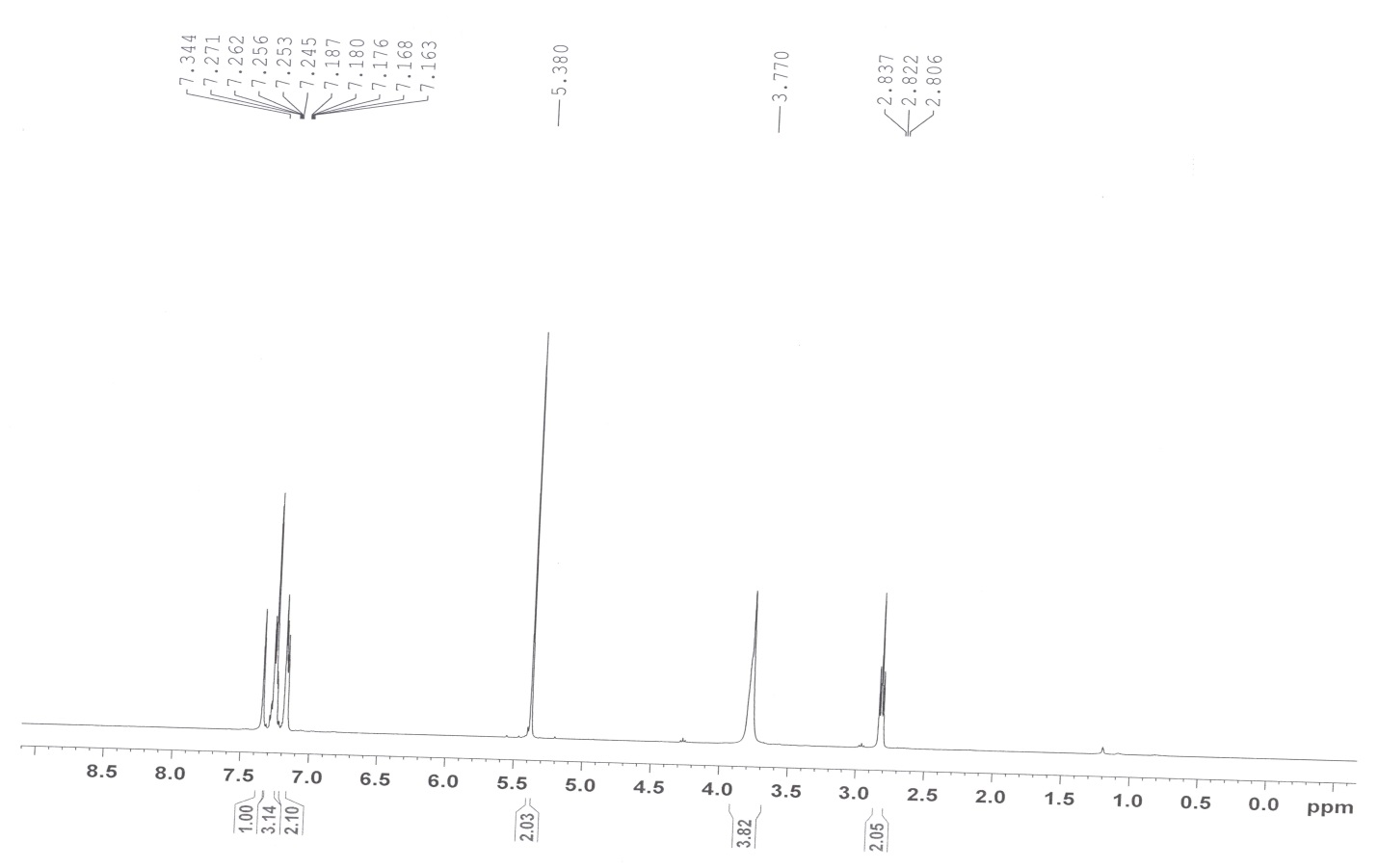
**Figure S15**. ^1^H NMR spectrum of **3ae** in CDCl_3_


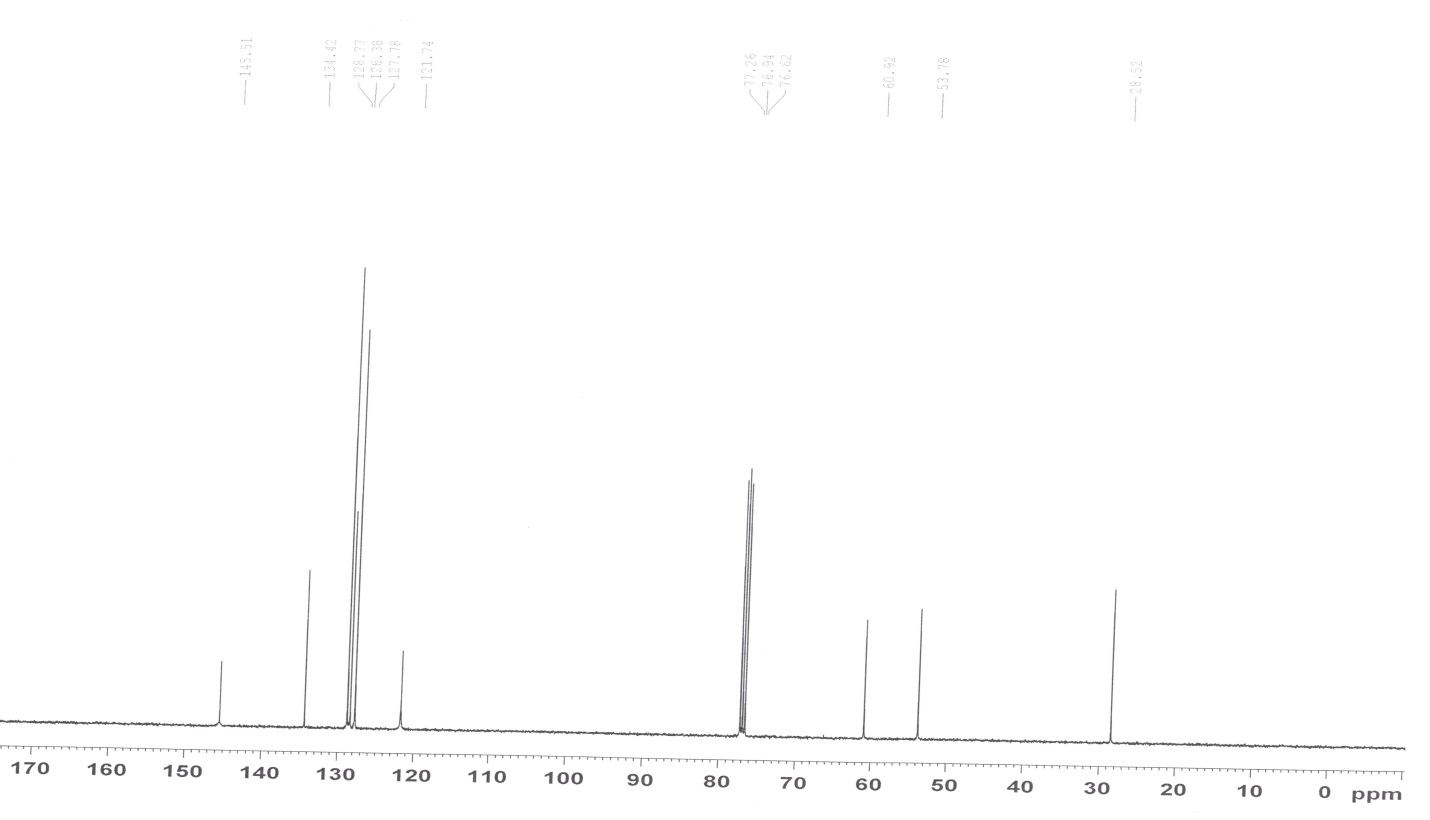
**Figure S16**. ^13^C NMR spectrum of **3ae** in CDCl_3_


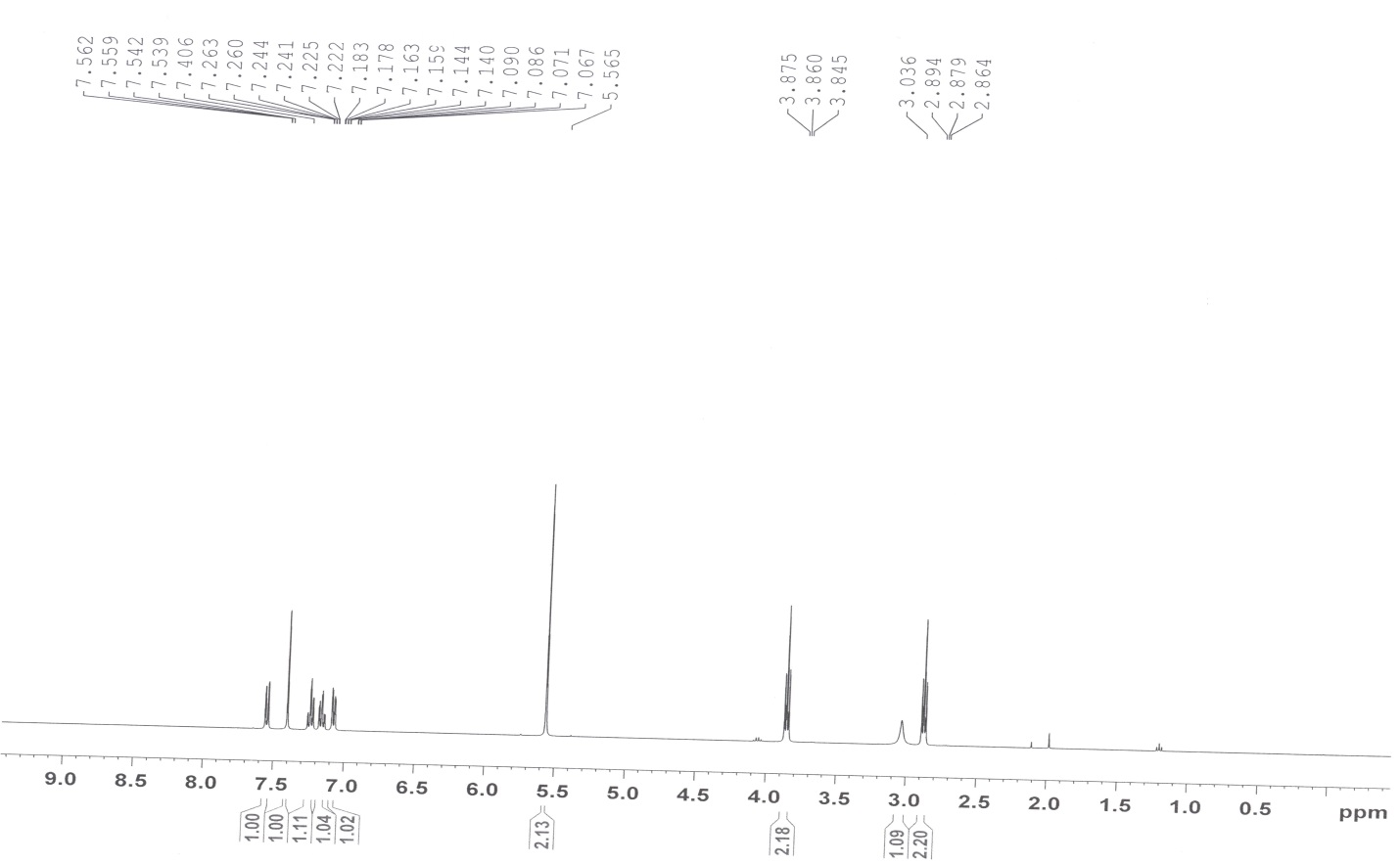


**Figure S17**. ^1^H NMR spectrum of **3be** in CDCl_3_


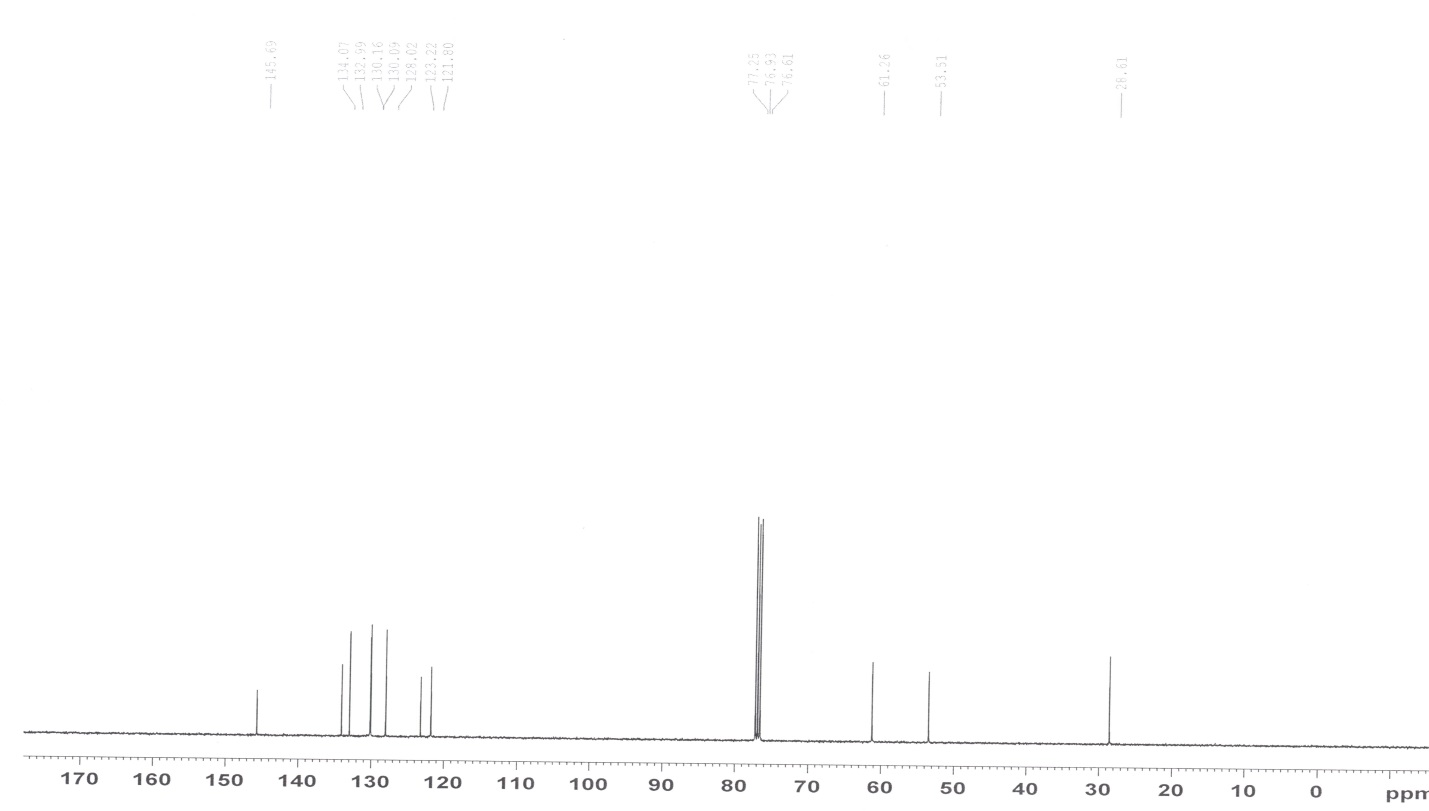


**Figure S18**. ^13^C NMR spectrum of **3be** in CDCl_3_


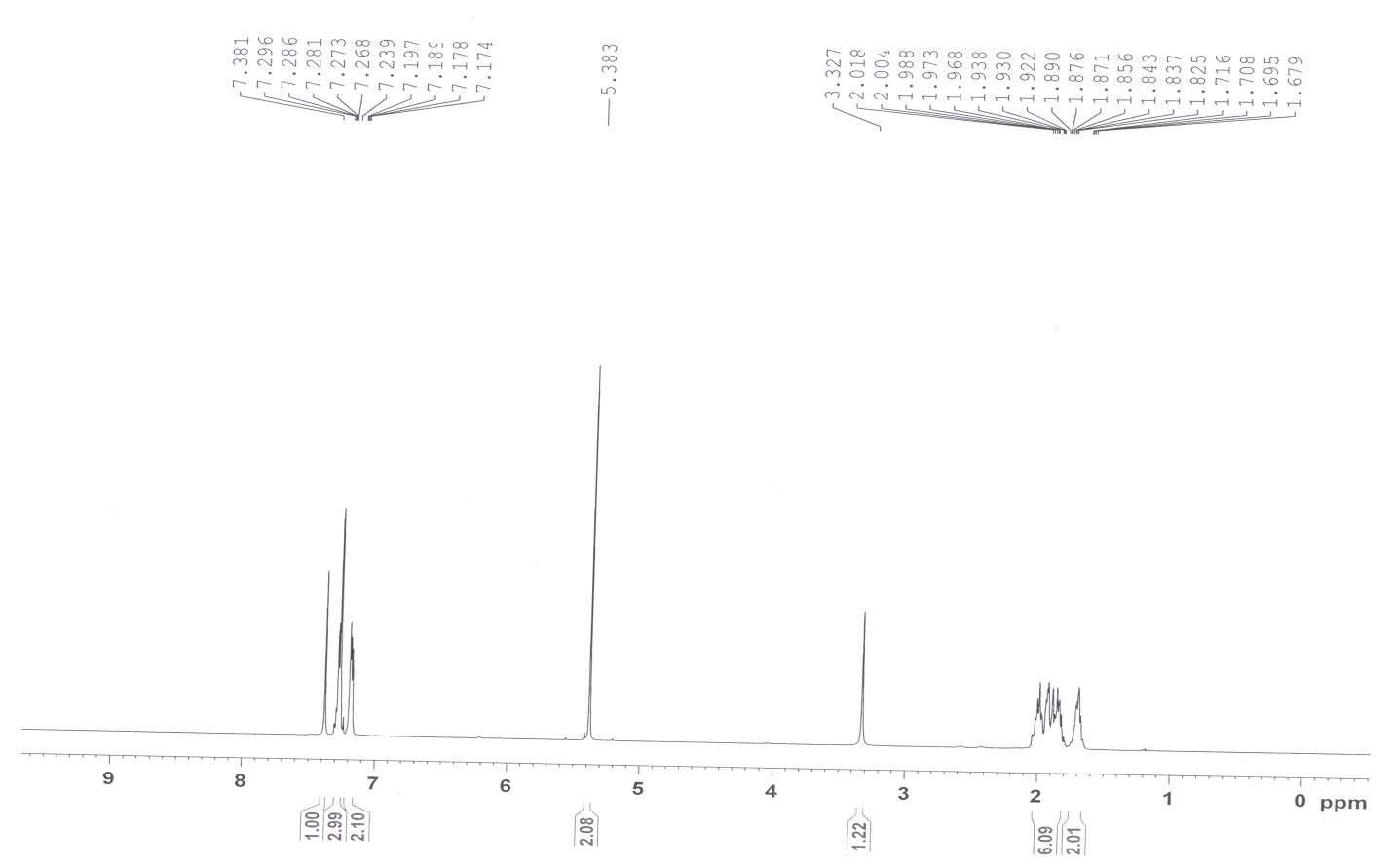


**Figure S19**. ^1^H NMR spectrum of **3af** in CDCl_3_


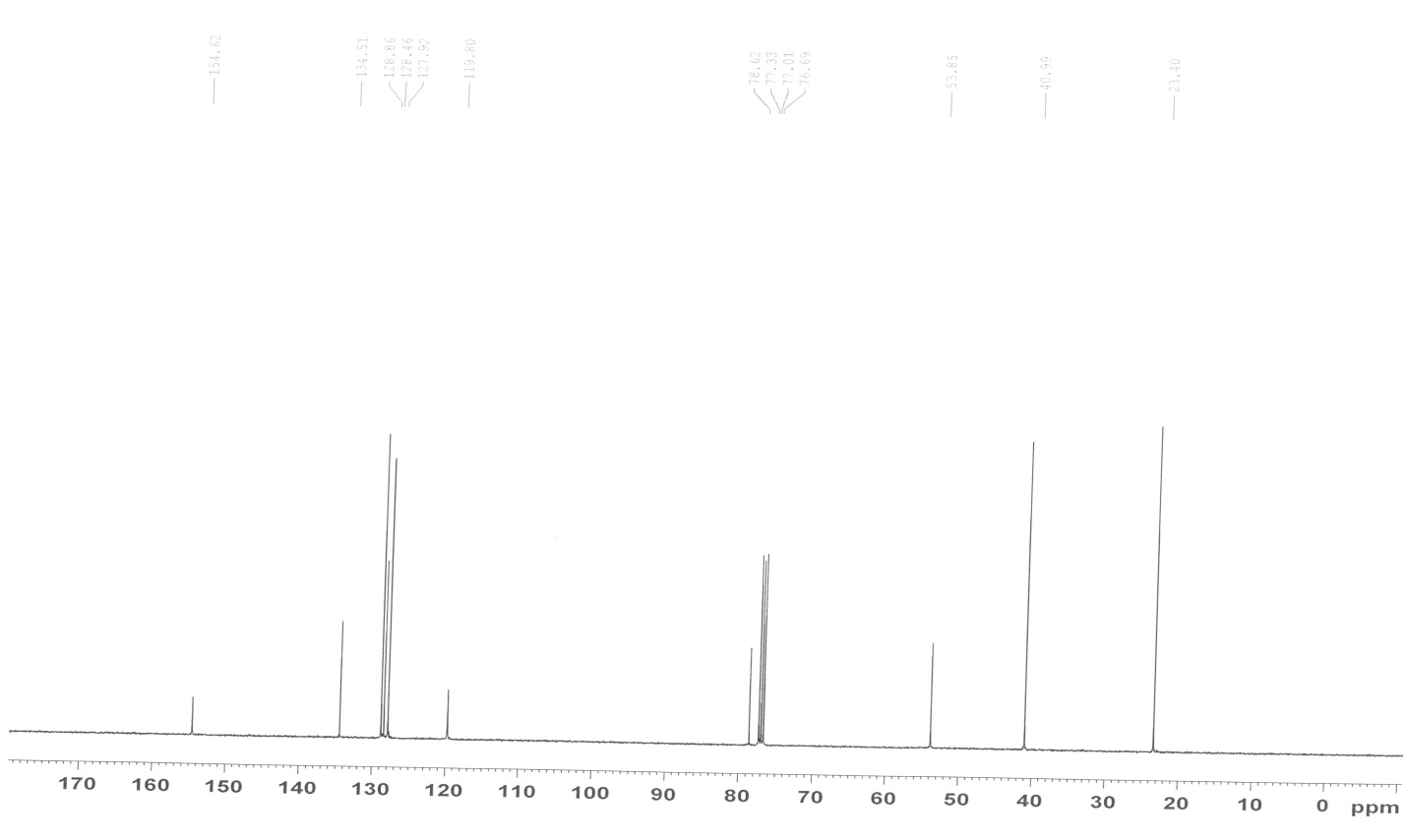


**Figure S20**. ^13^C NMR spectrum of **3af** in CDCl_3_


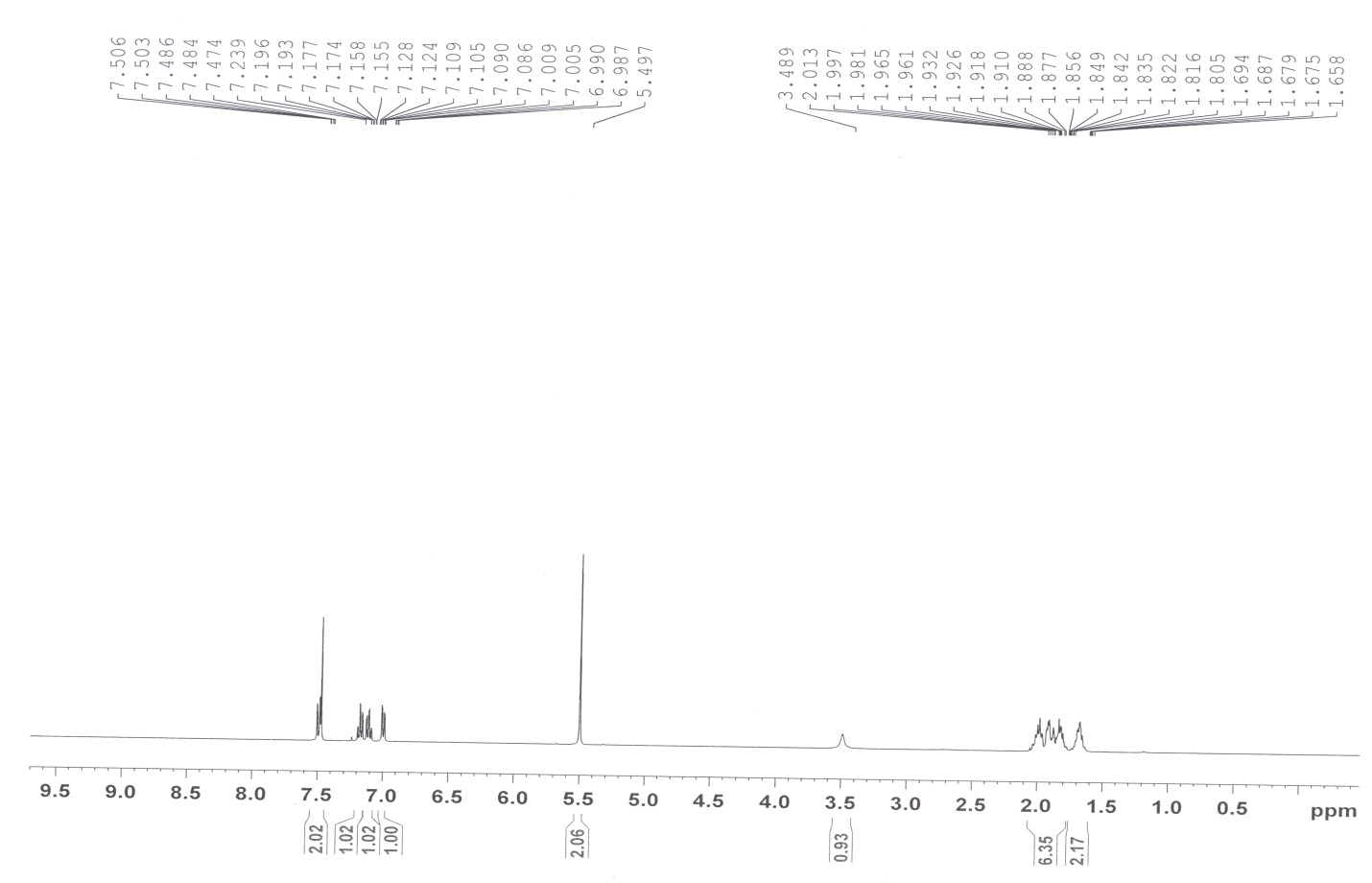


**Figure S21**. ^1^H NMR spectrum of **3bf** in CDCl_3_


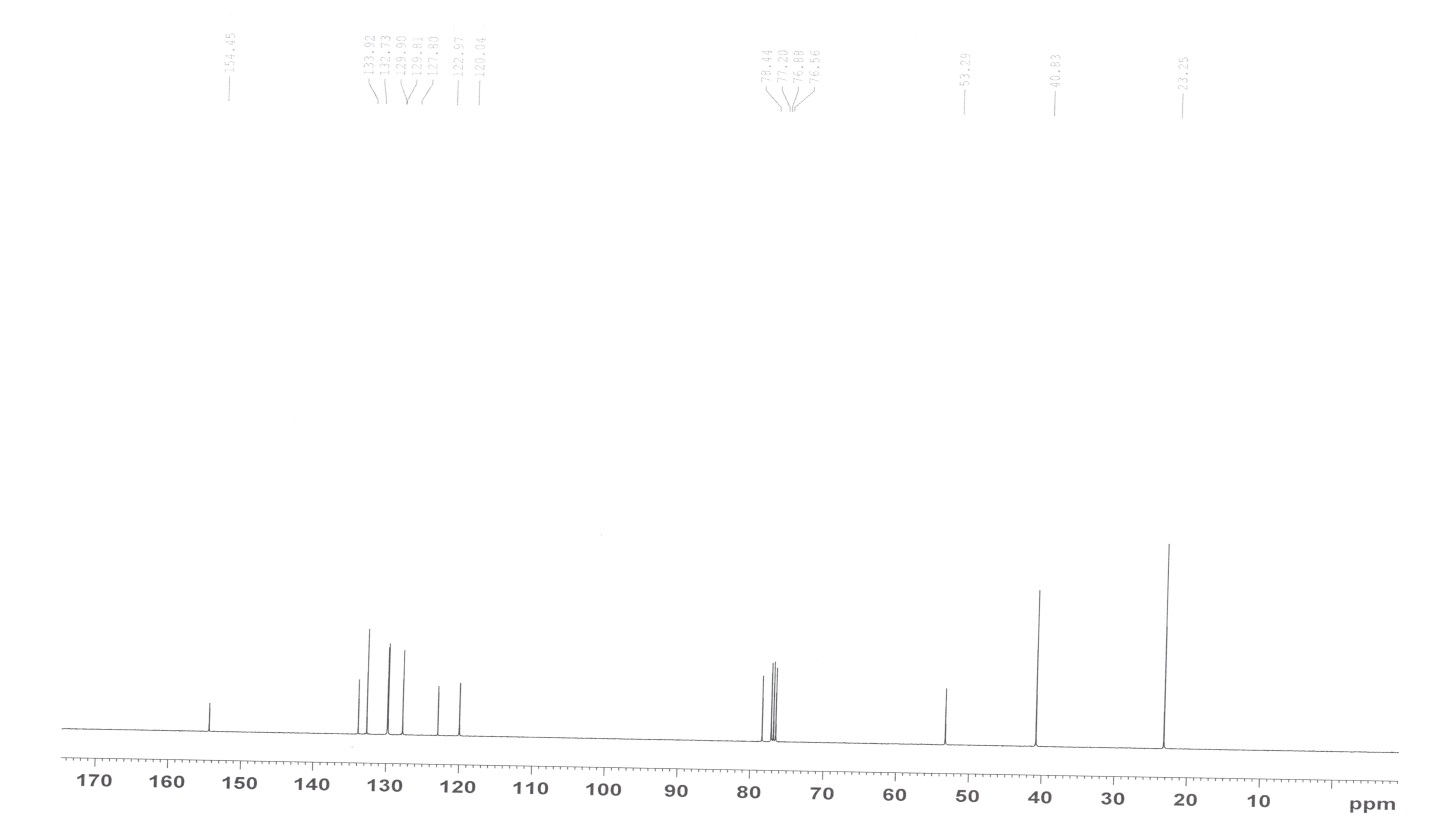


**Figure S22**. ^13^C NMR spectrum of **3bf** in CDCl_3_


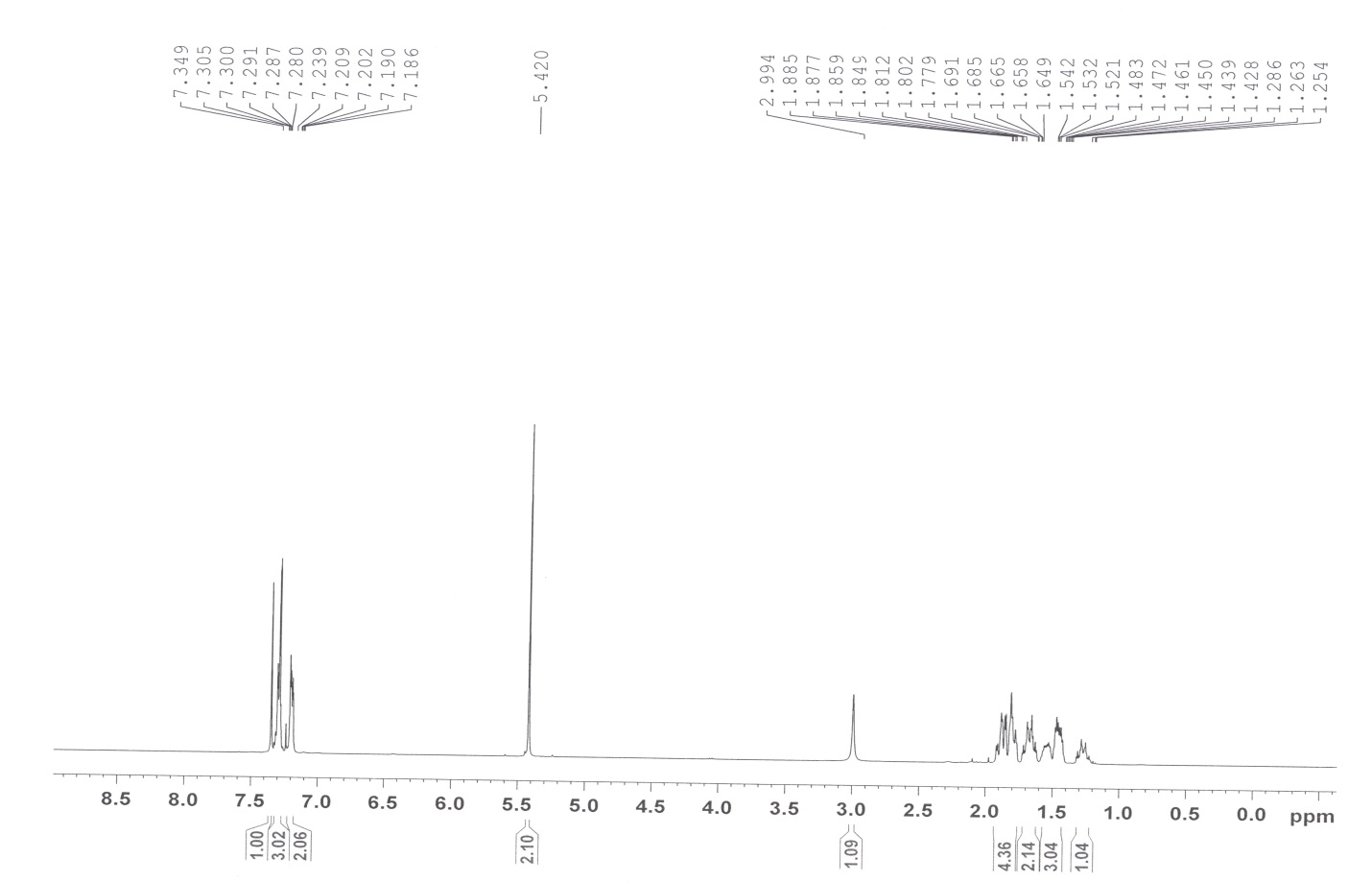


**Figure S23**. ^1^H NMR spectrum of **3ag** in CDCl_3_


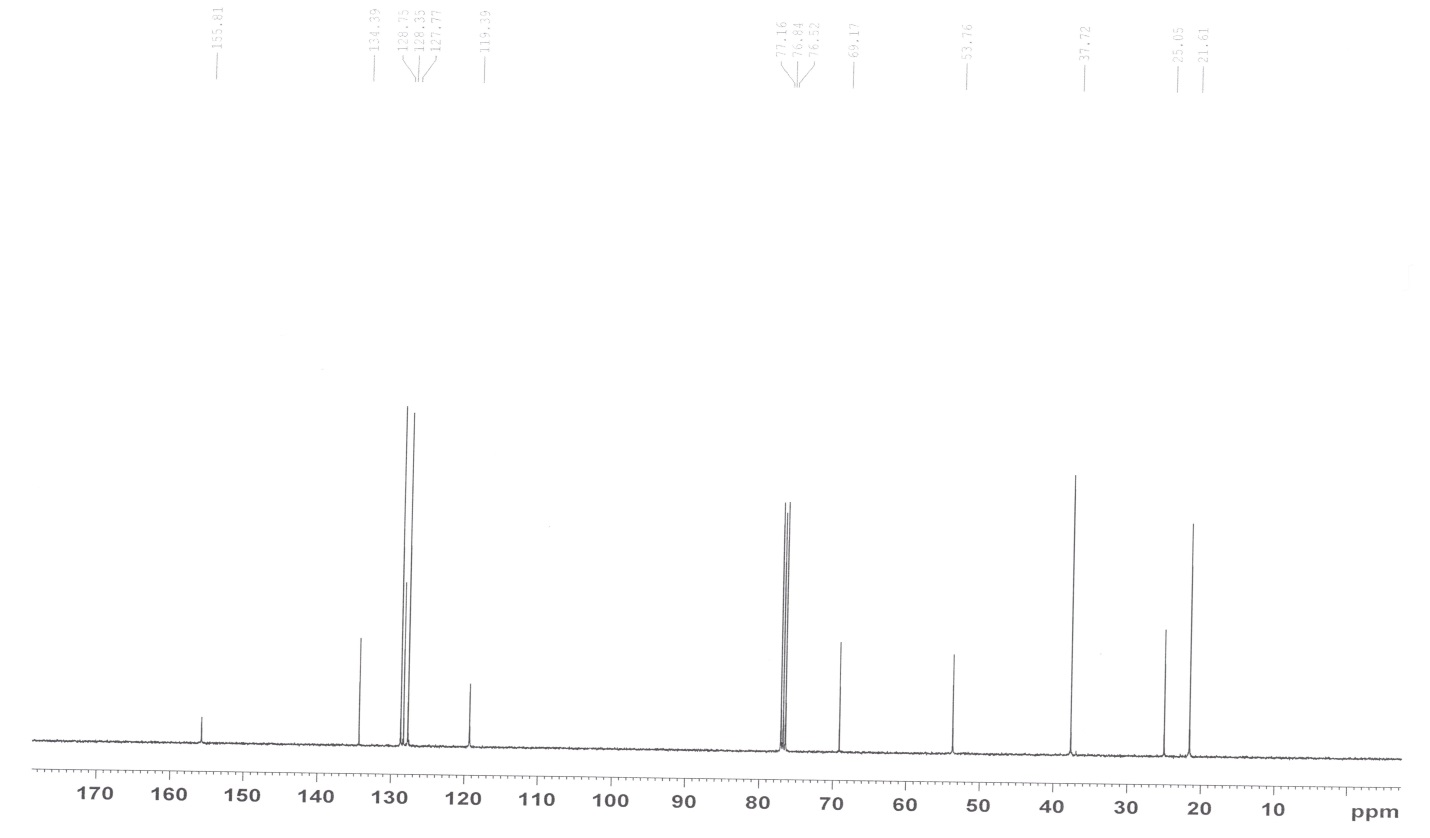


**Figure S24**. ^13^C NMR spectrum of **3ag** in CDCl_3_


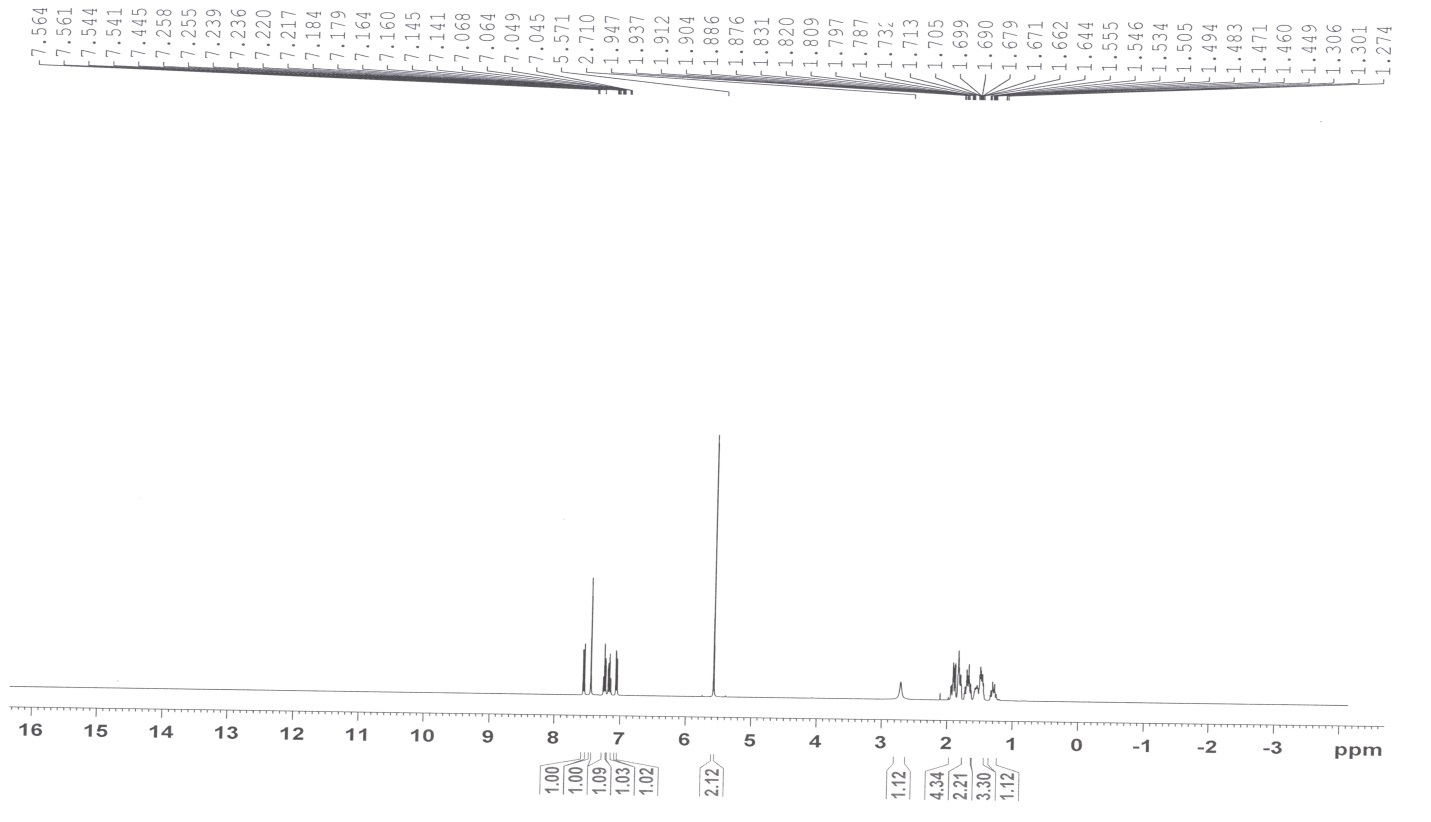


**Figure S25**. ^1^H NMR spectrum of **3bg** in CDCl_3_


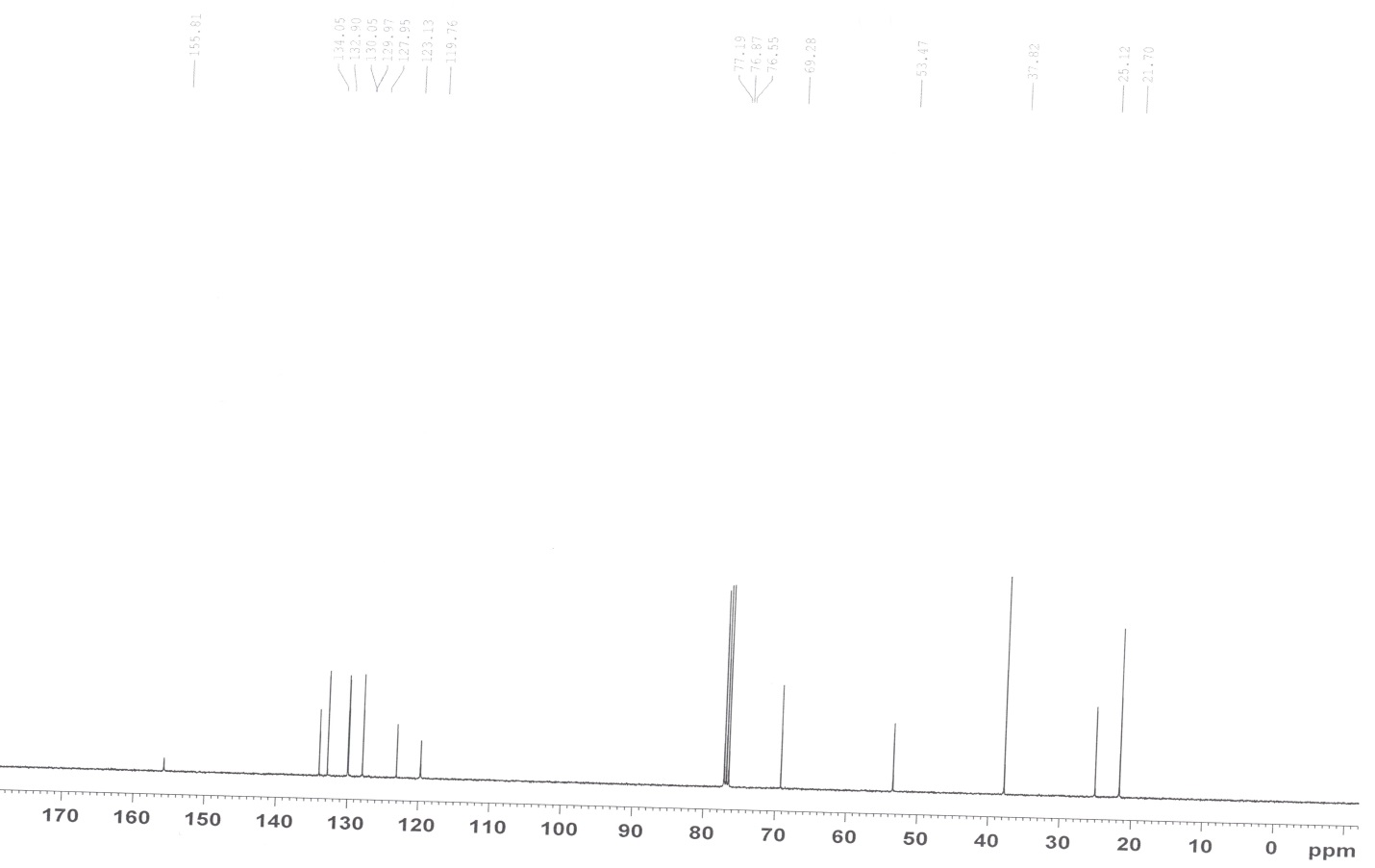


**Figure S26**. ^13^C NMR spectrum of **3bg** in CDCl_3_


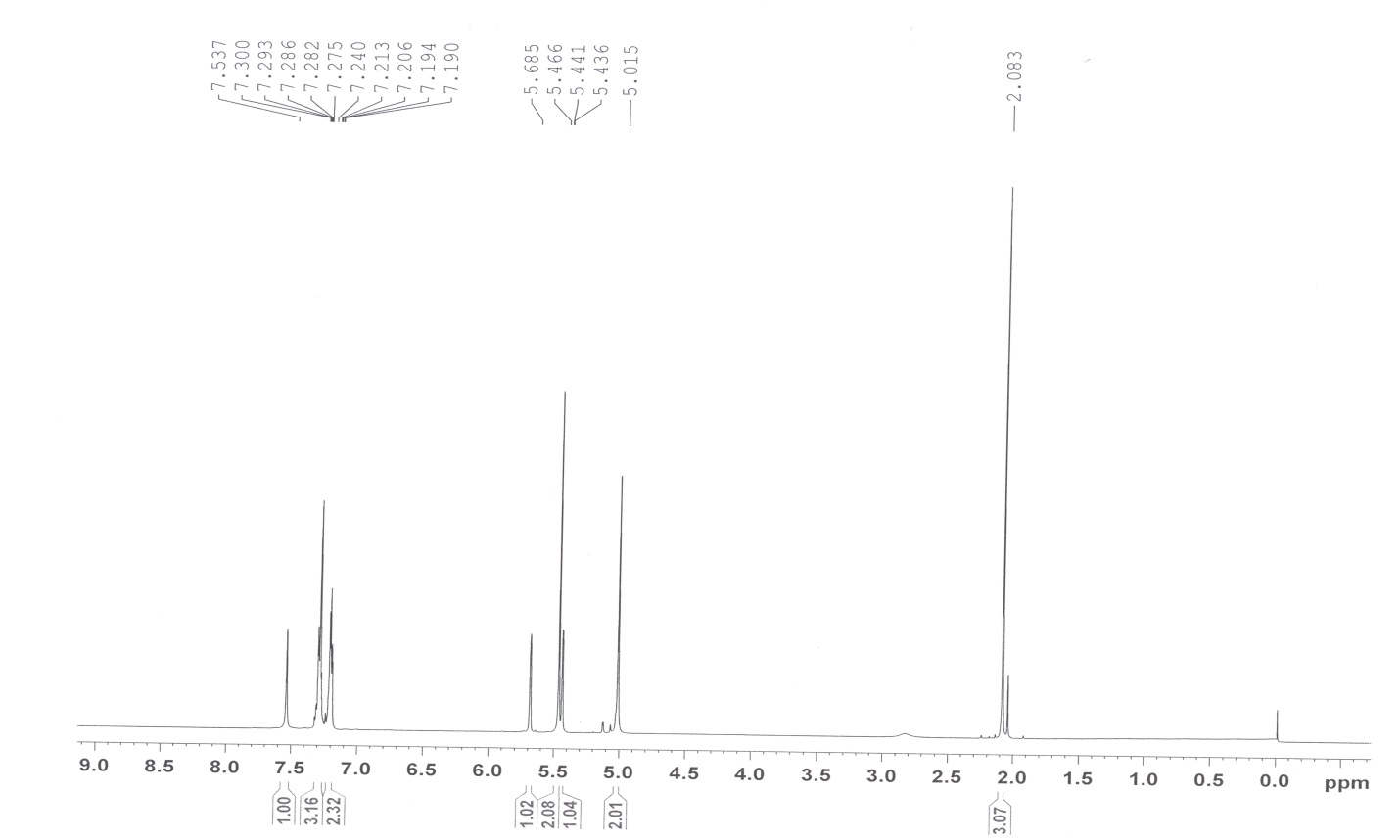


**Figure S27**. ^1^H NMR spectrum of **3ah** in CDCl_3_


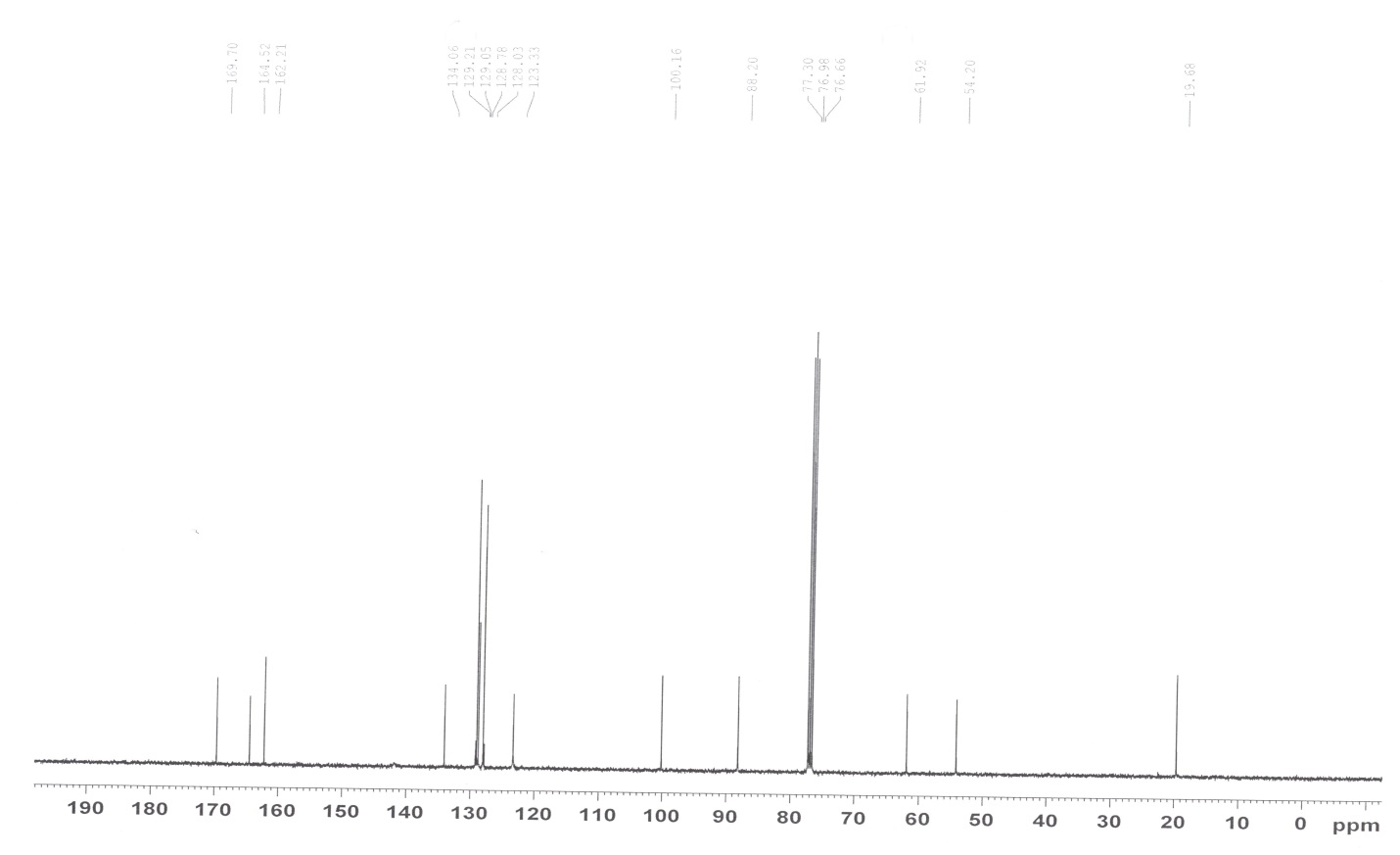


**Figure S28**. ^13^C NMR spectrum of **3ah** in CDCl_3_


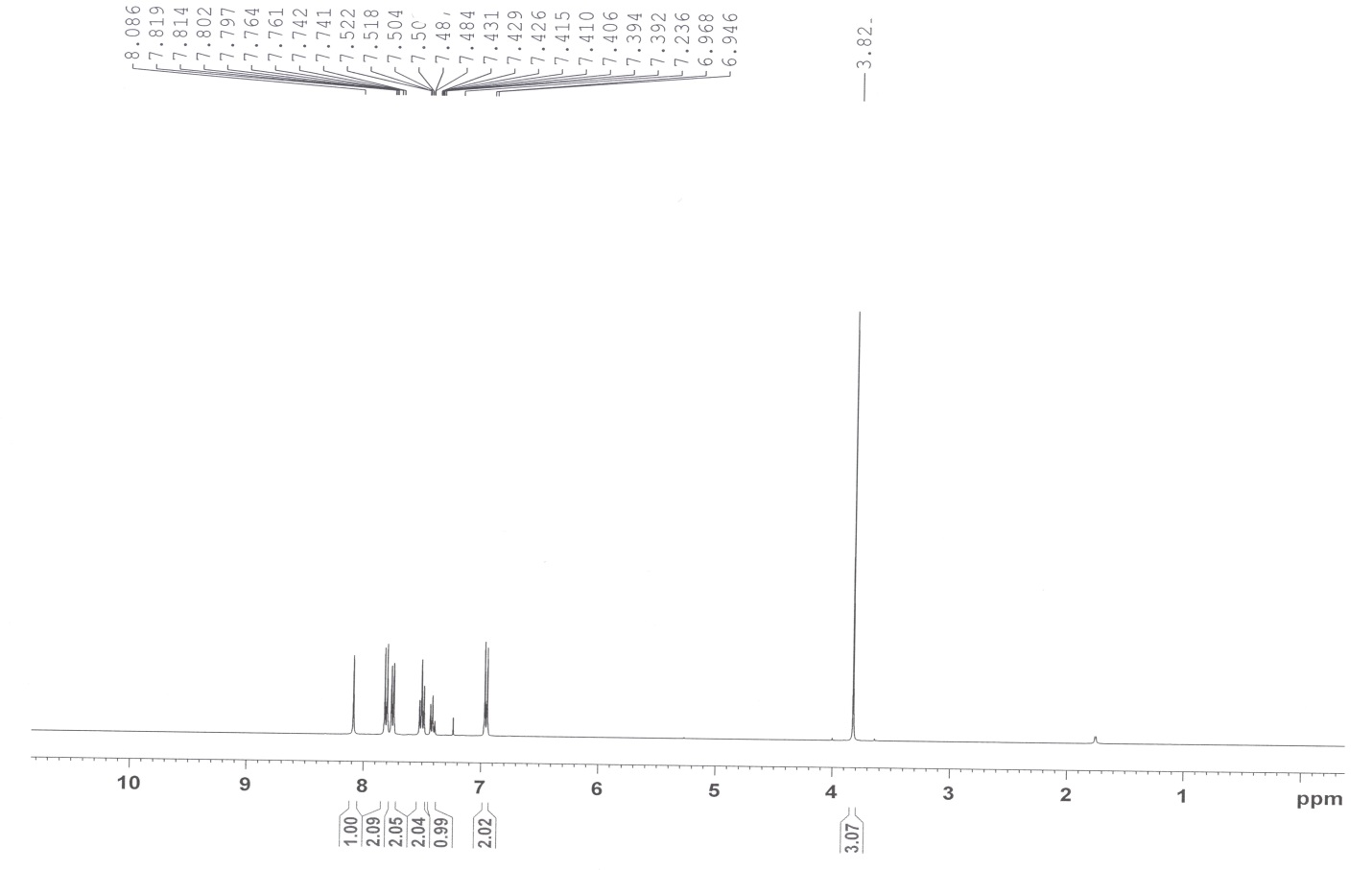


**Figure S29**. ^1^H NMR spectrum of **3cb** in CDCl_3_


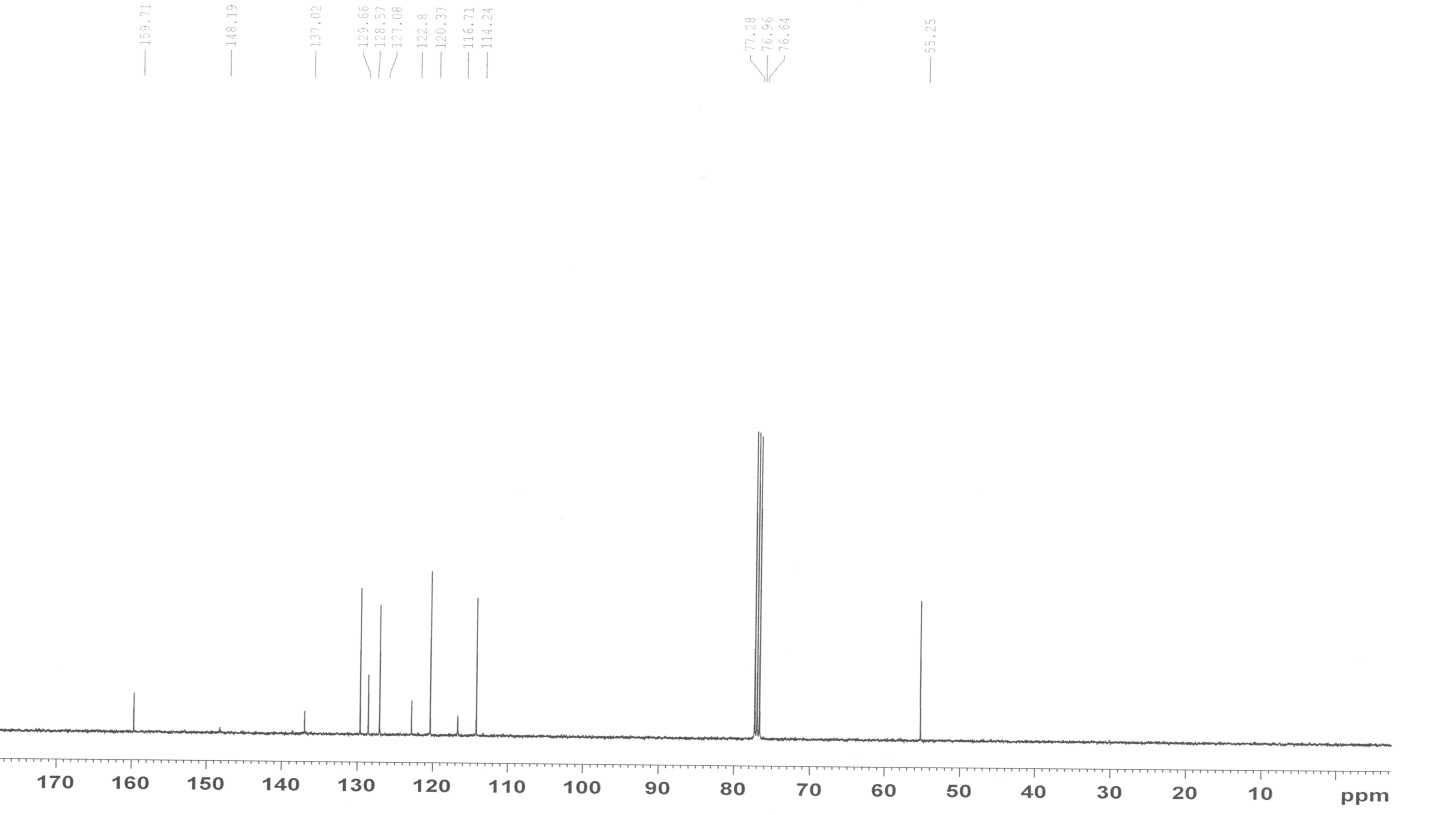


**Figure S30**. ^13^C NMR spectrum of **3cb** in CDCl_3_


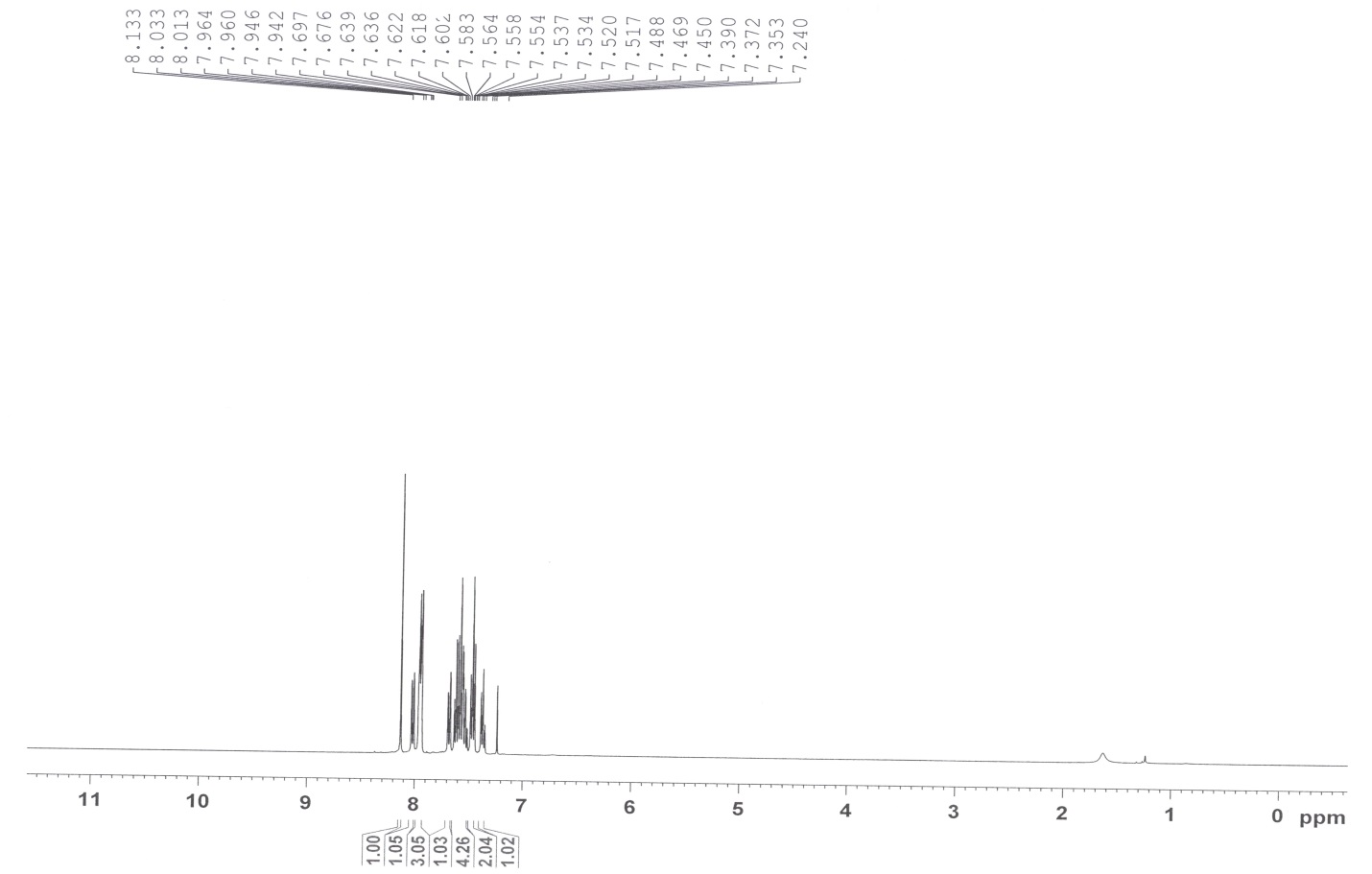


**Figure S31**. ^1^H NMR spectrum of **3da** in CDCl_3_


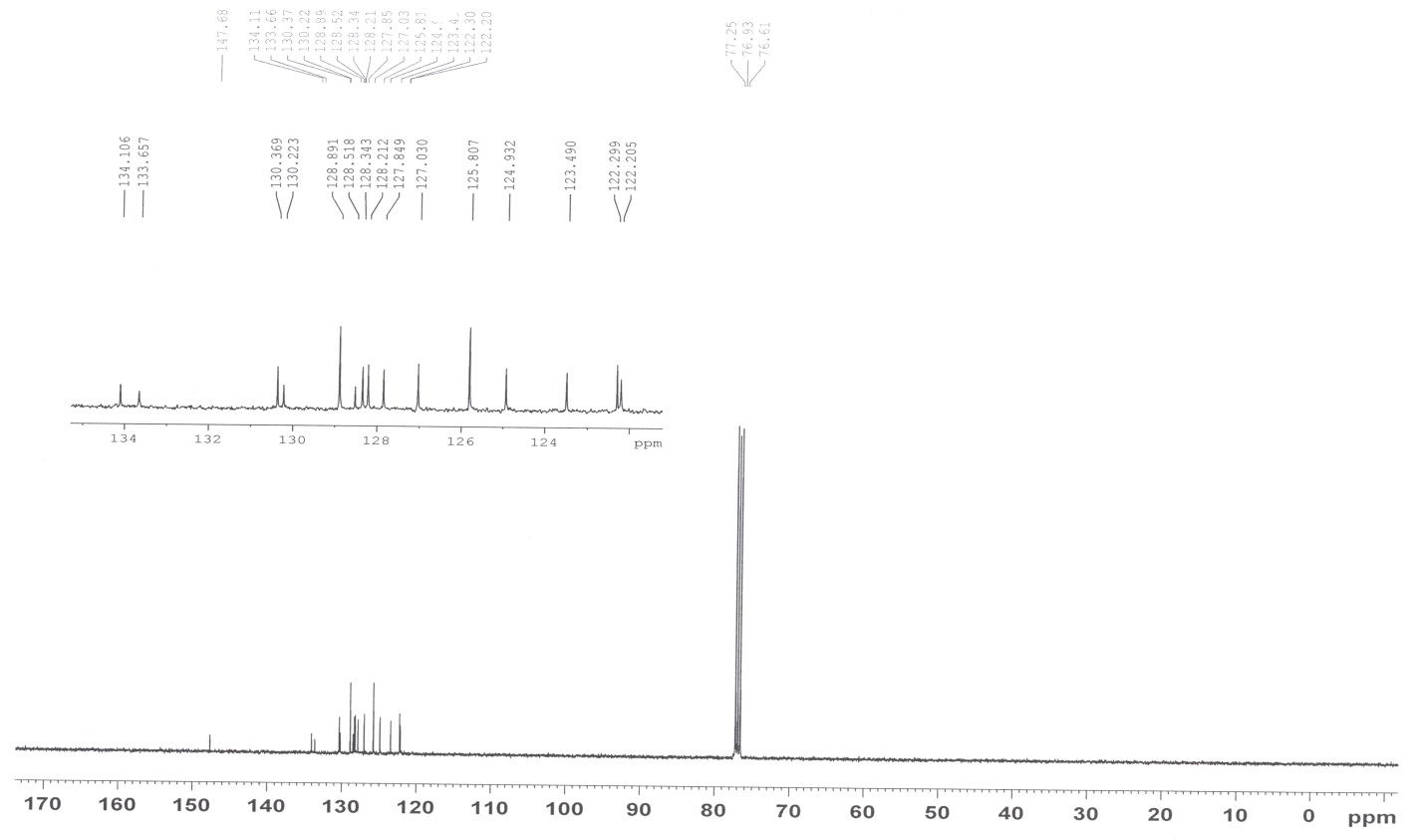


**Figure S32**. ^13^C NMR spectrum of **3da** in CDCl_3_


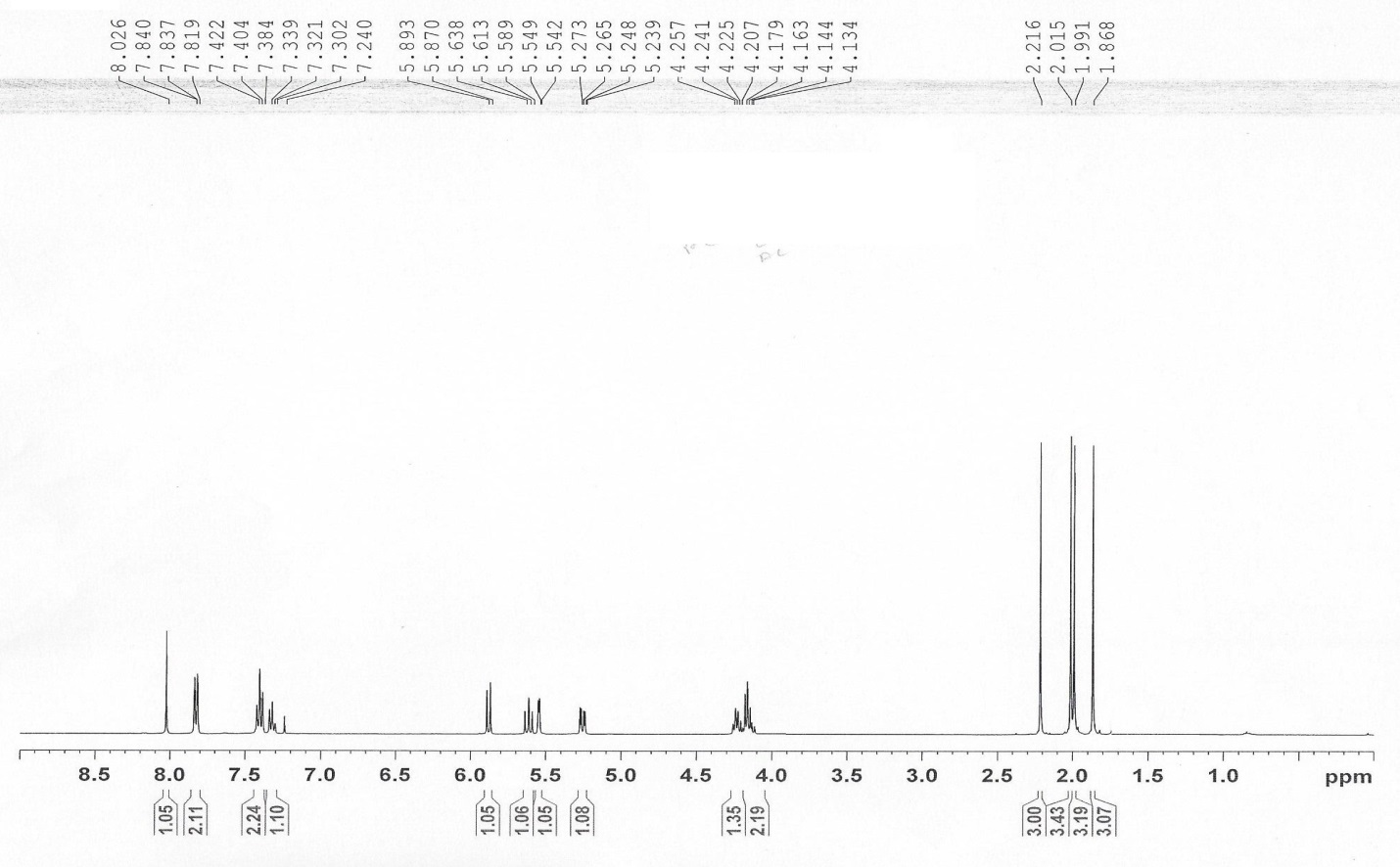


**Figure S33**. ^1^H NMR spectrum of **3ea** in CDCl_3_


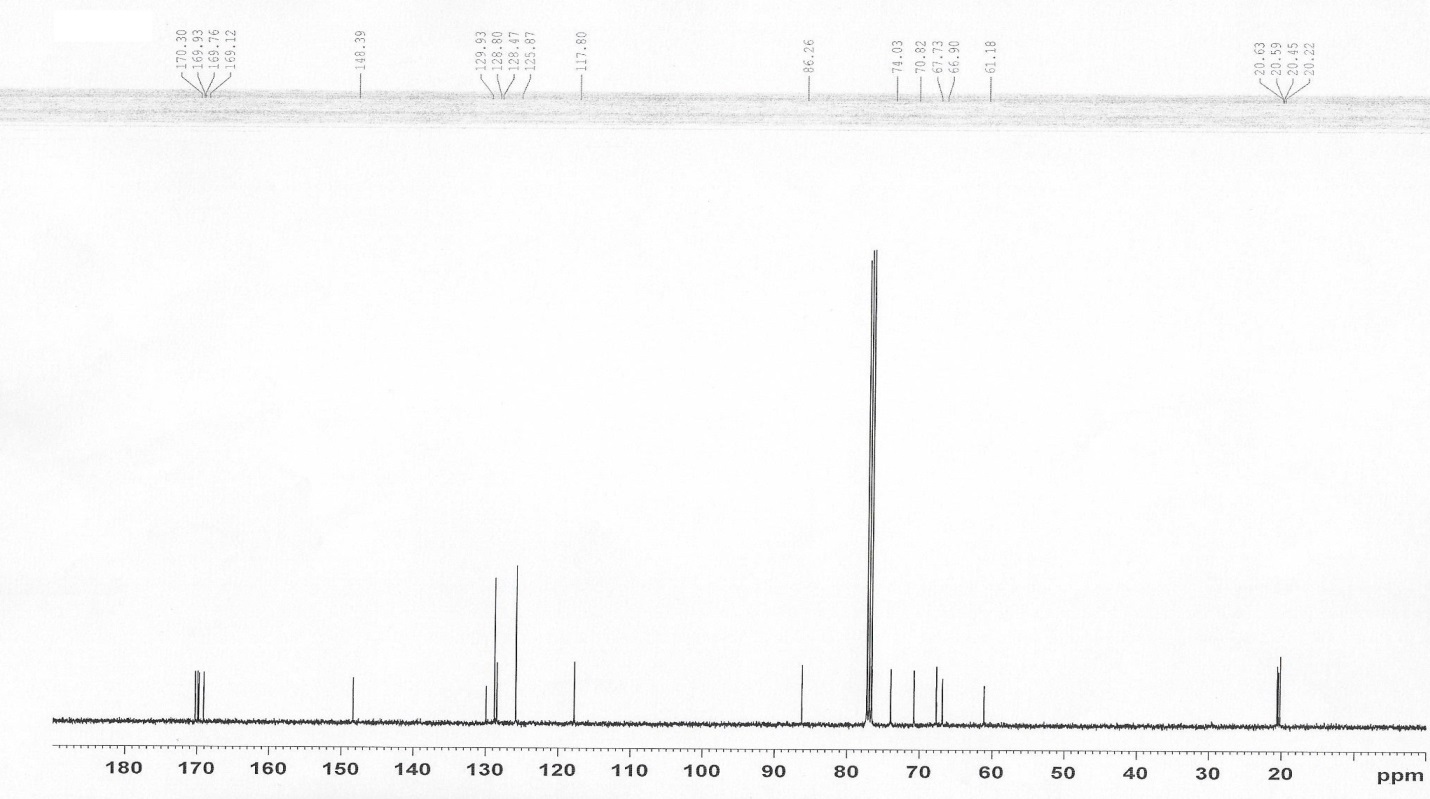


**Figure S34**. ^13^C NMR spectrum of **3ea** in CDCl_3_


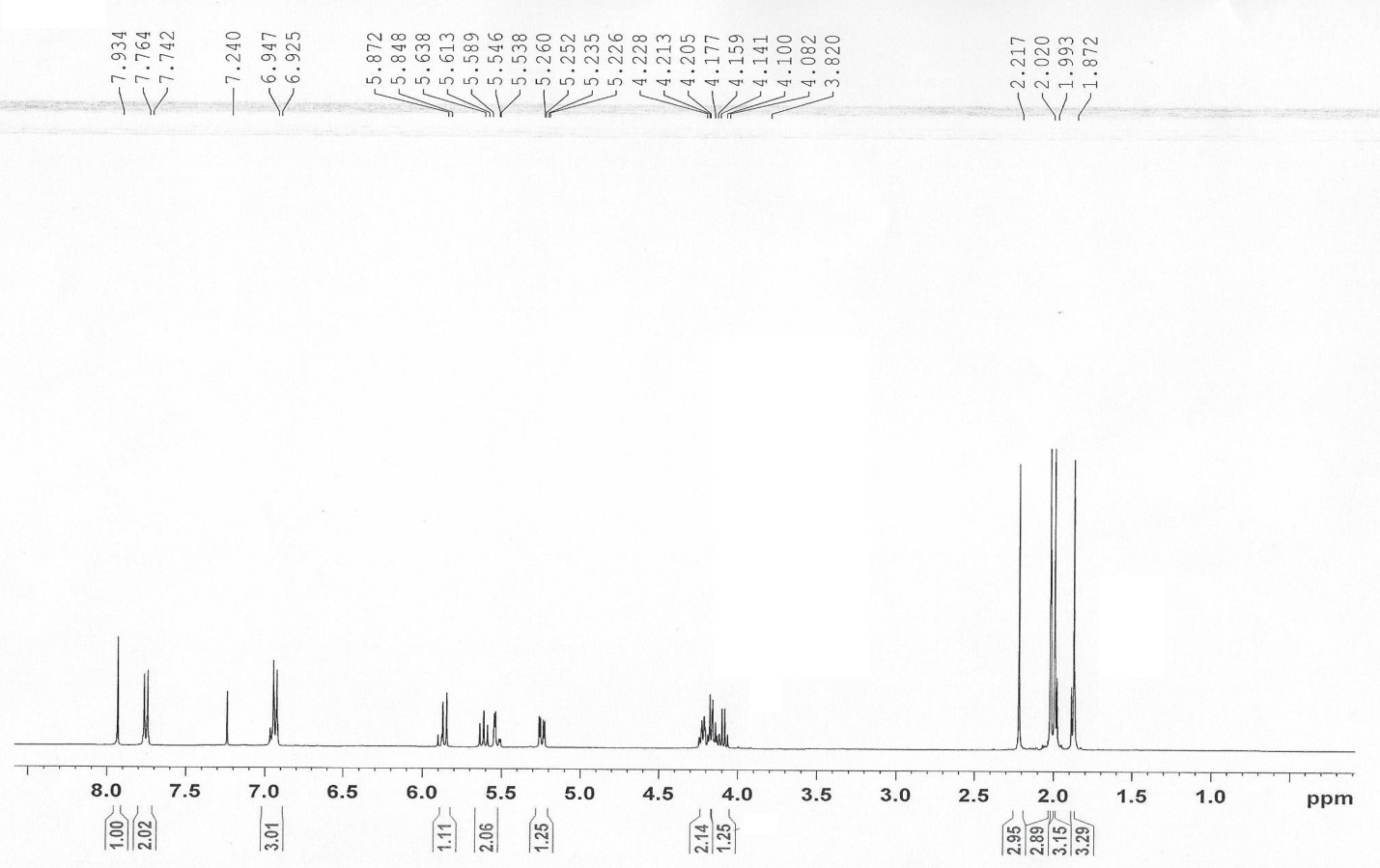


**Figure S35**. ^1^H NMR spectrum of **3fa** in CDCl_3_


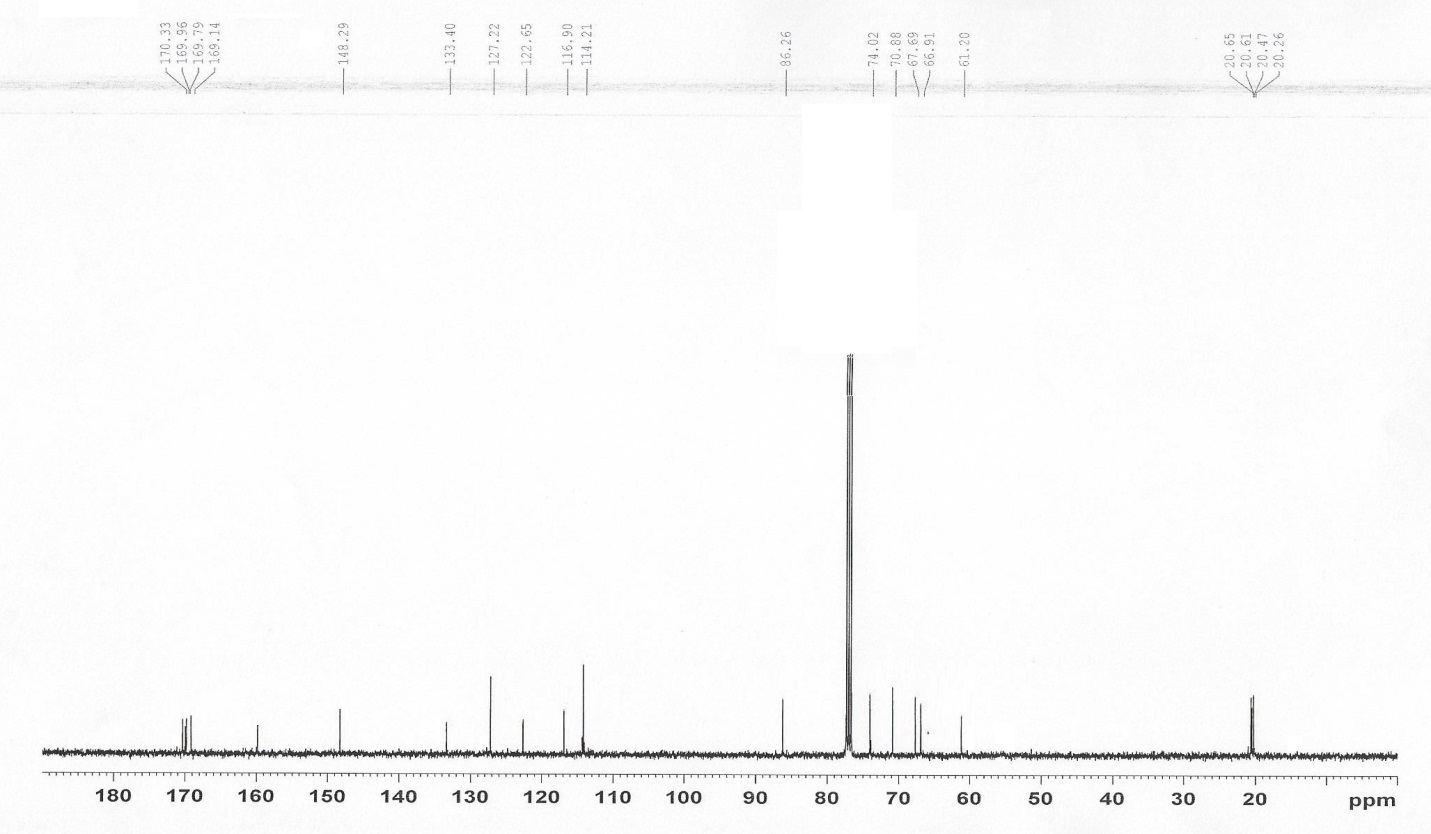


**Figure S36**. ^13^C NMR spectrum of **3fa** in CDCl_3_


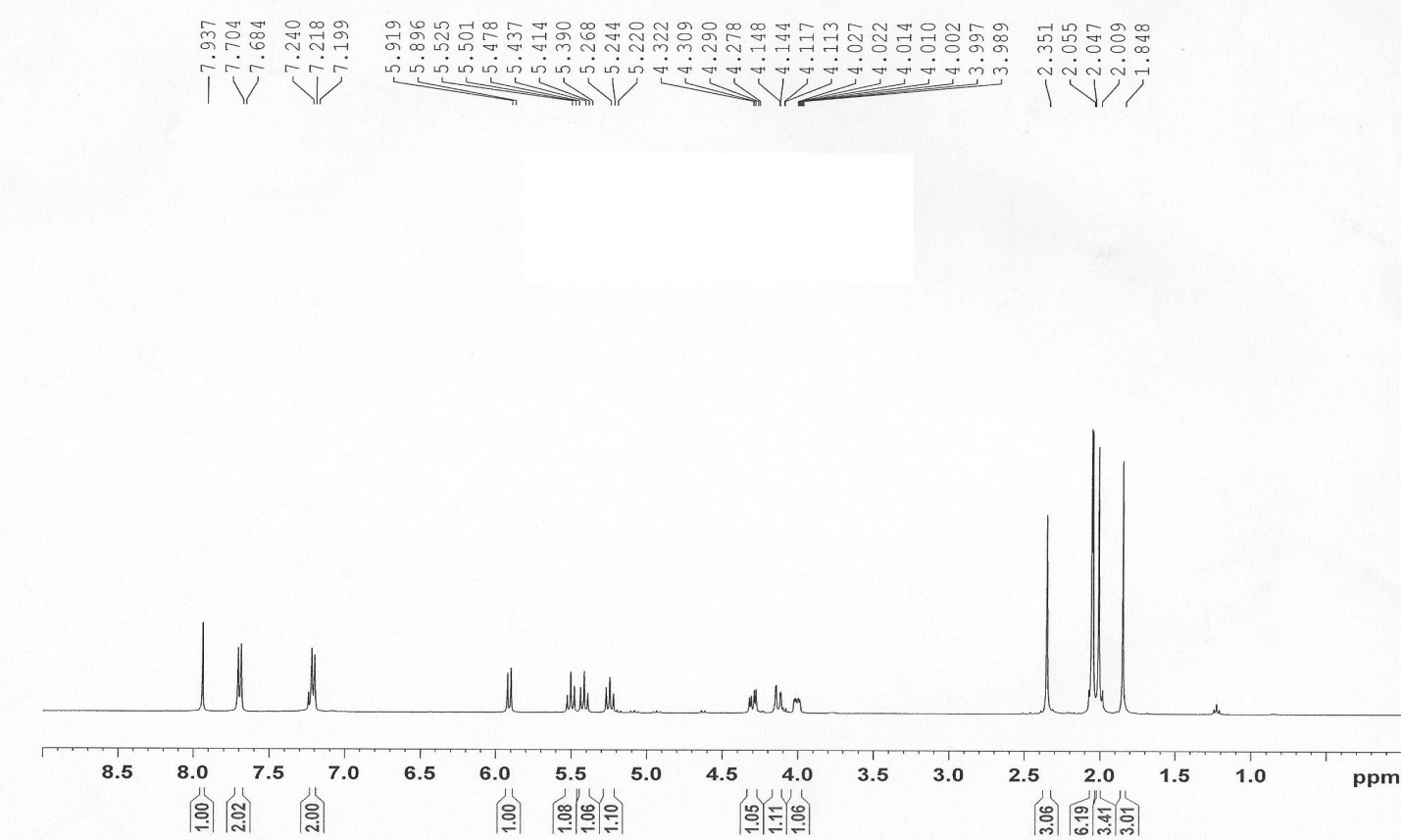


**Figure S37**. ^1^H NMR spectrum of **3ei** in CDCl_3_


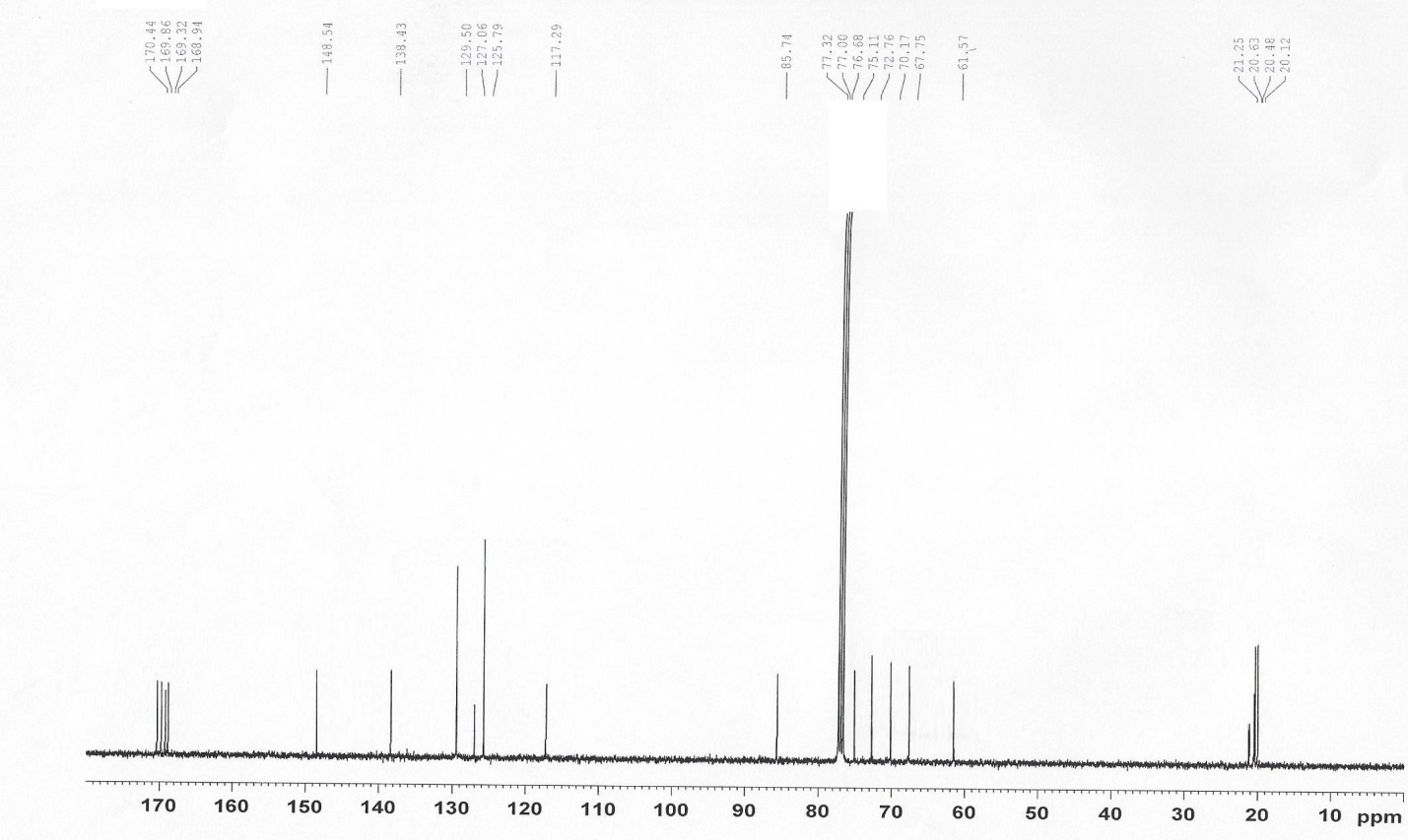


**Figure S38**. ^13^C NMR spectrum of **3ei** in CDCl_3_


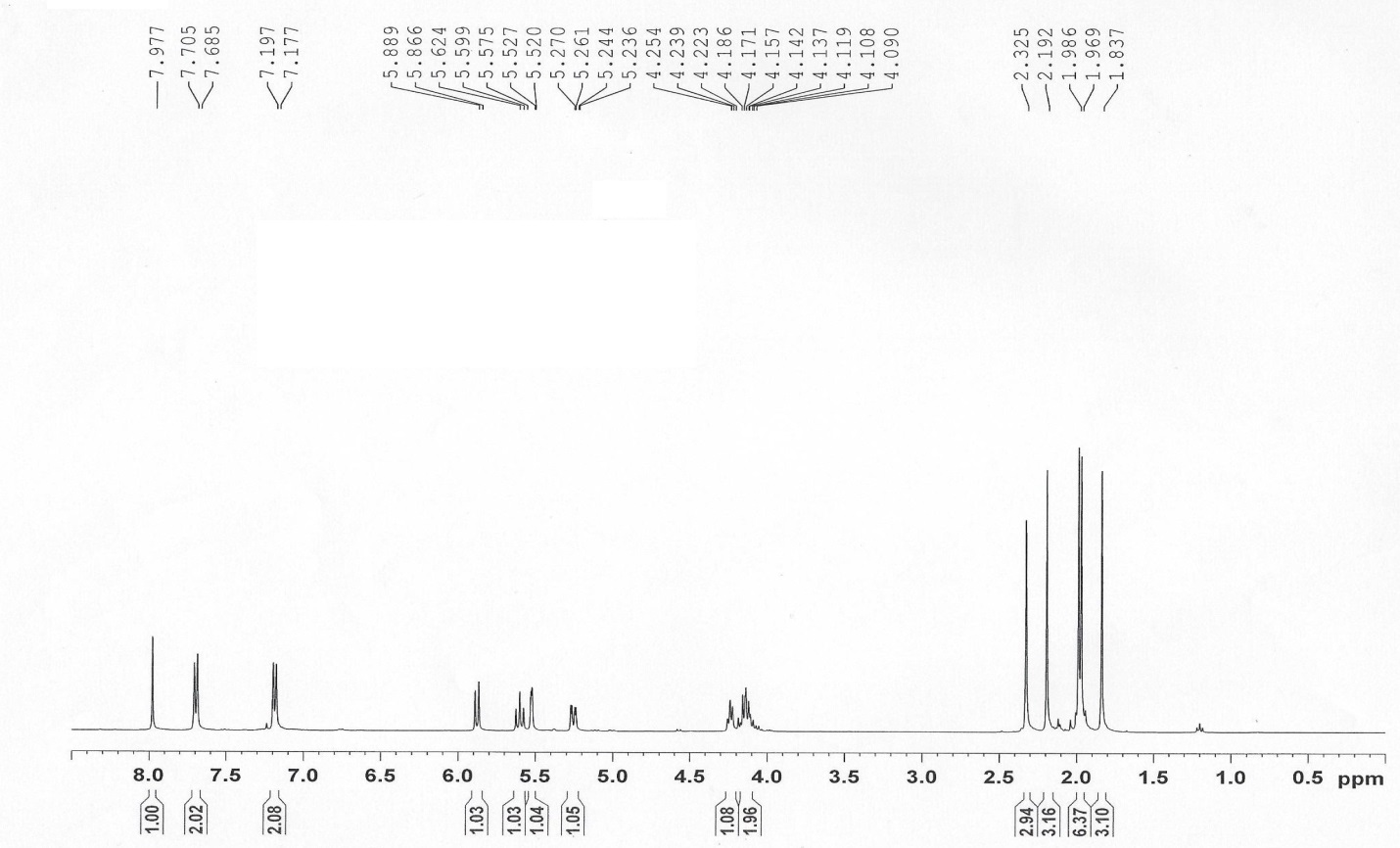


**Figure S39**. ^1^H NMR spectrum of **3fi** in CDCl_3_


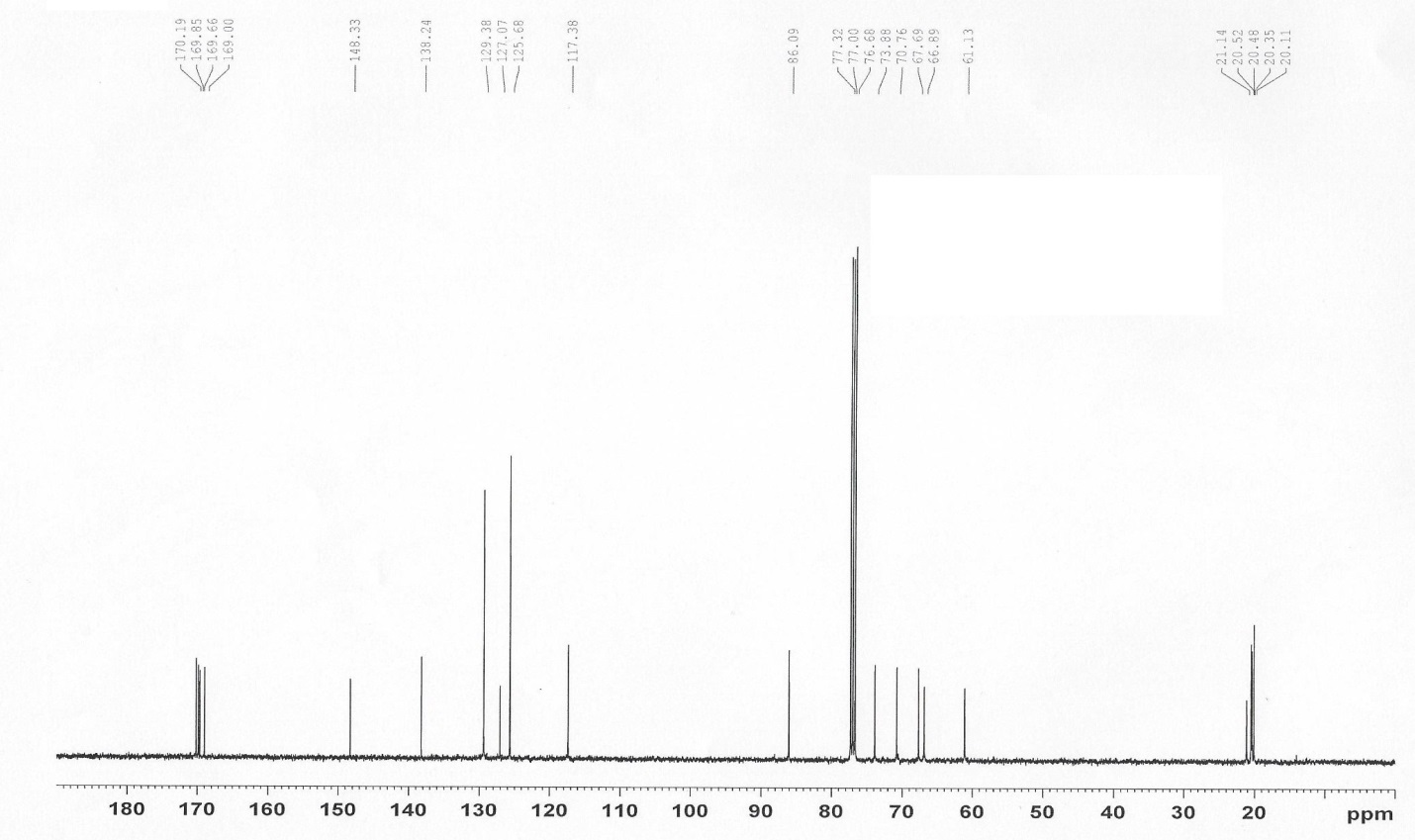


**Figure S40**. ^13^C NMR spectrum of **3fi** in CDCl_3_


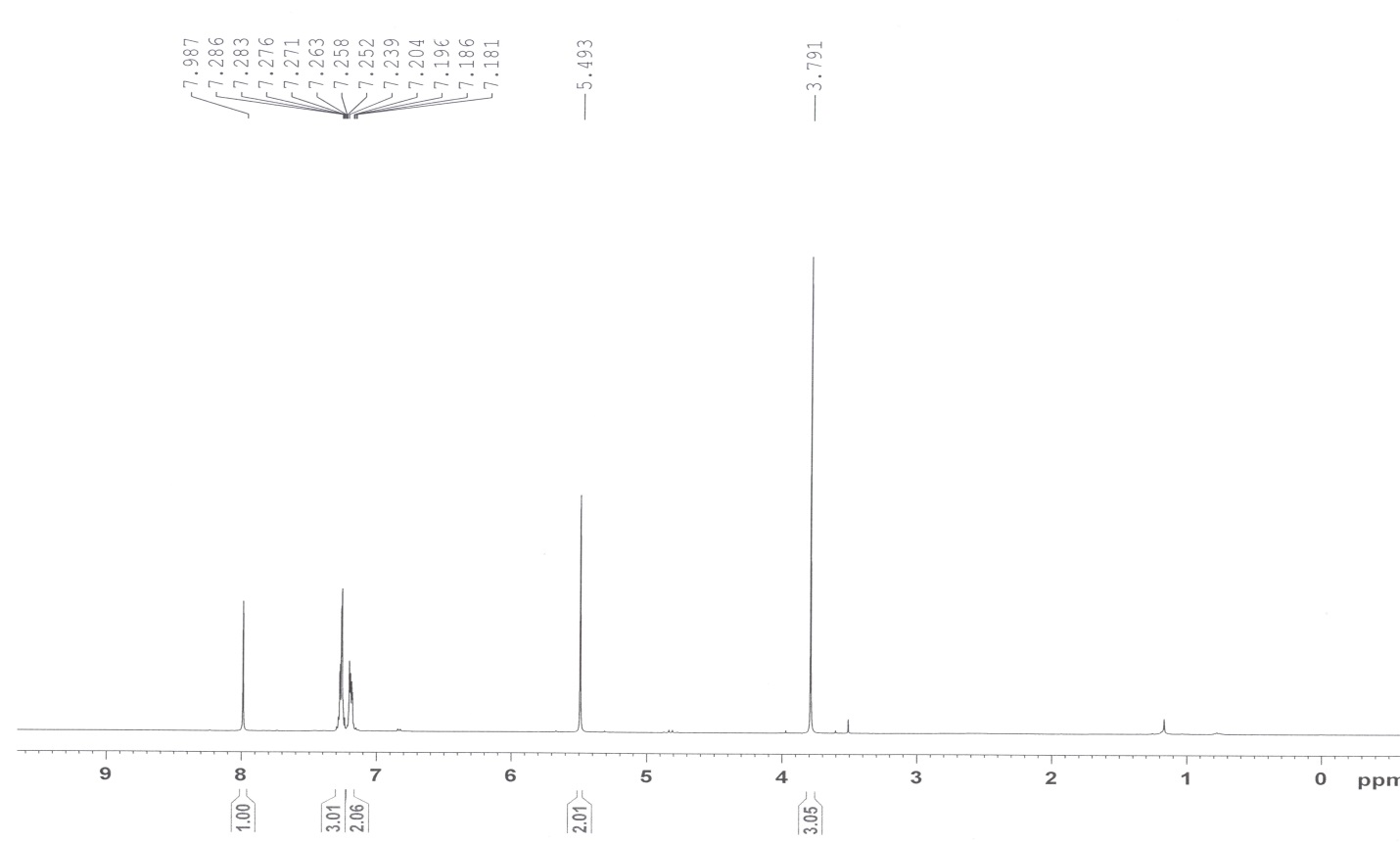


**Figure S41**. ^1^H NMR spectrum of **5aj** in CDCl_3_


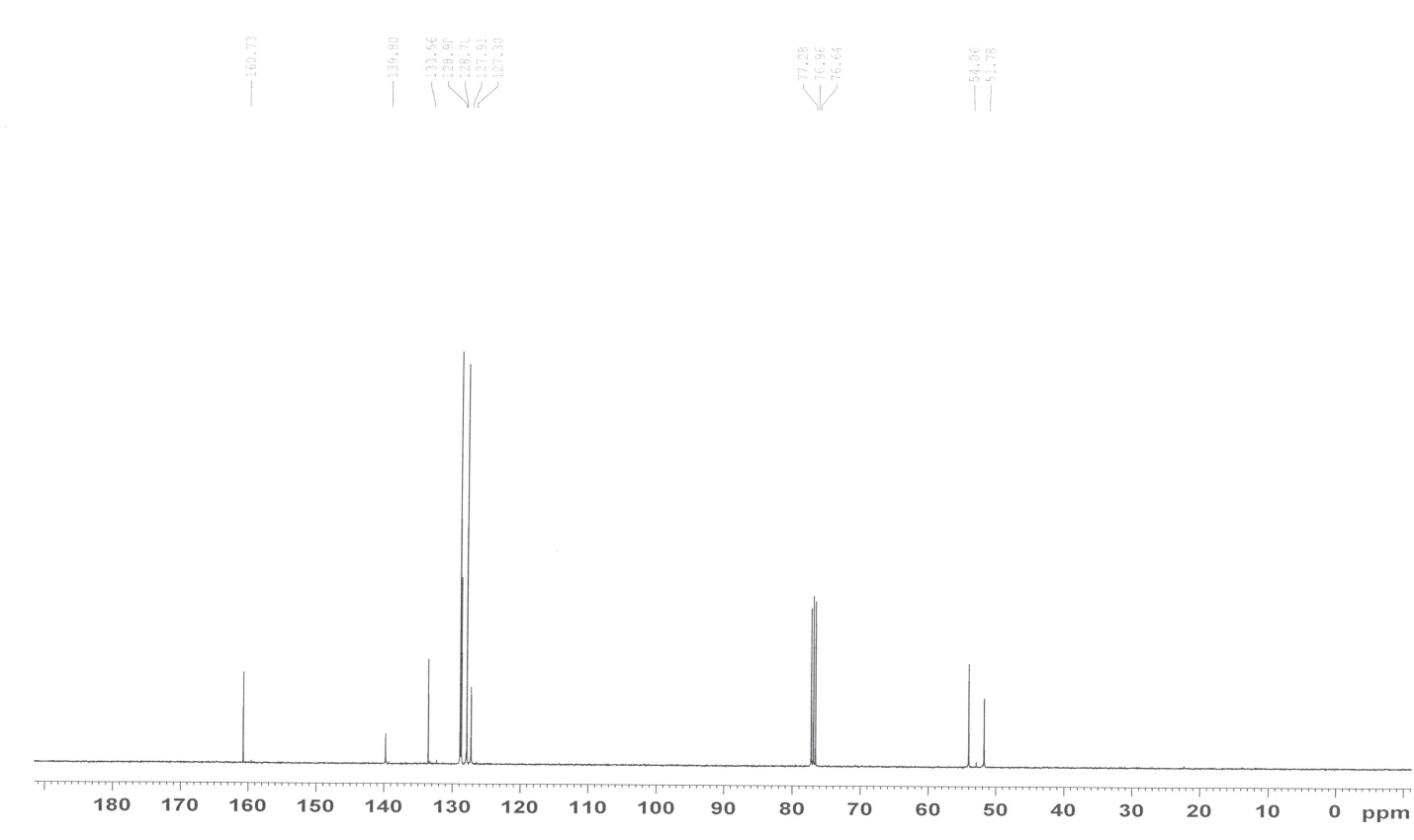


**Figure S42**. ^13^C NMR spectrum of **5aj** in CDCl_3_


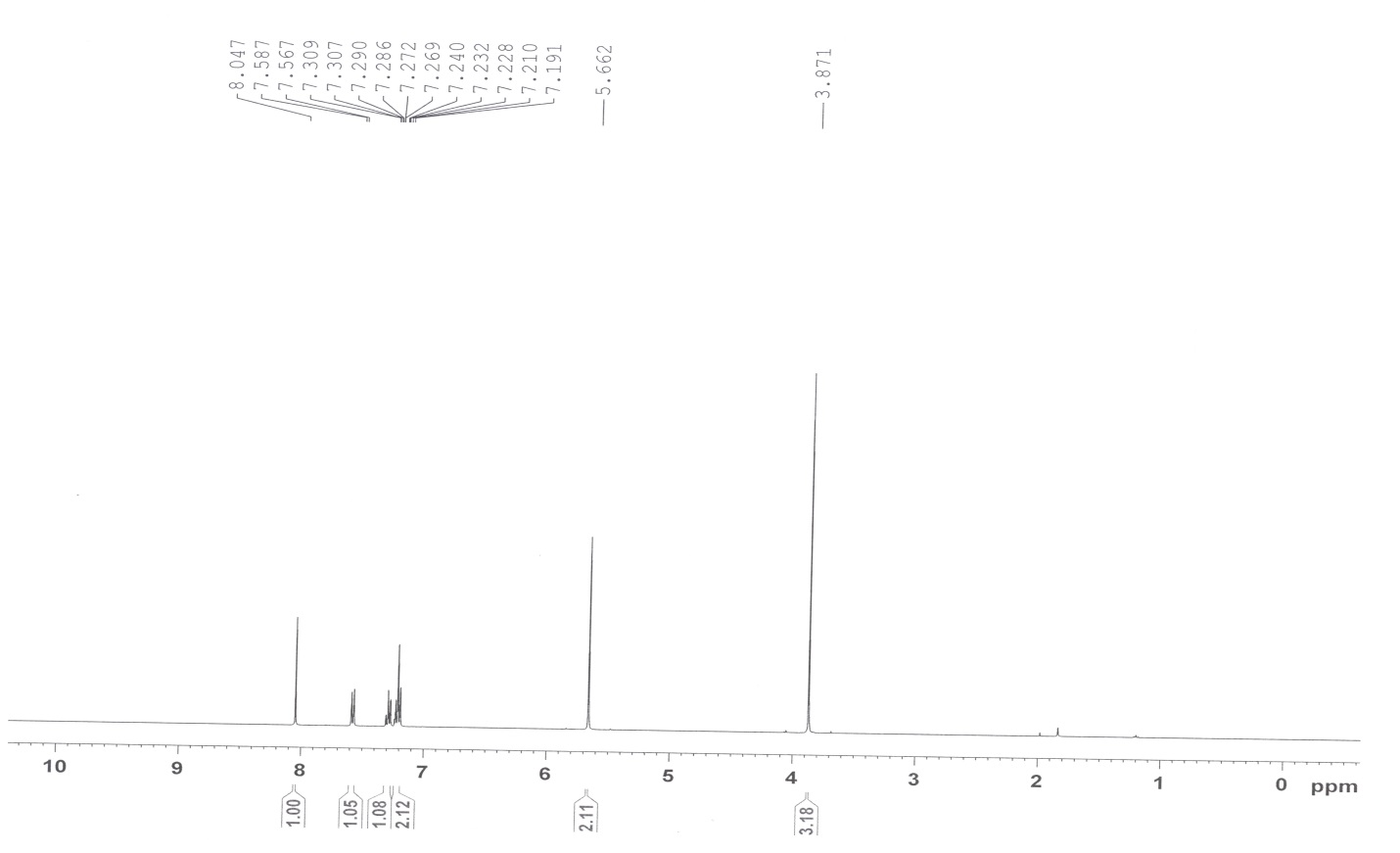


**Figure S43**. ^1^H NMR spectrum of **5bj** in CDCl_3_


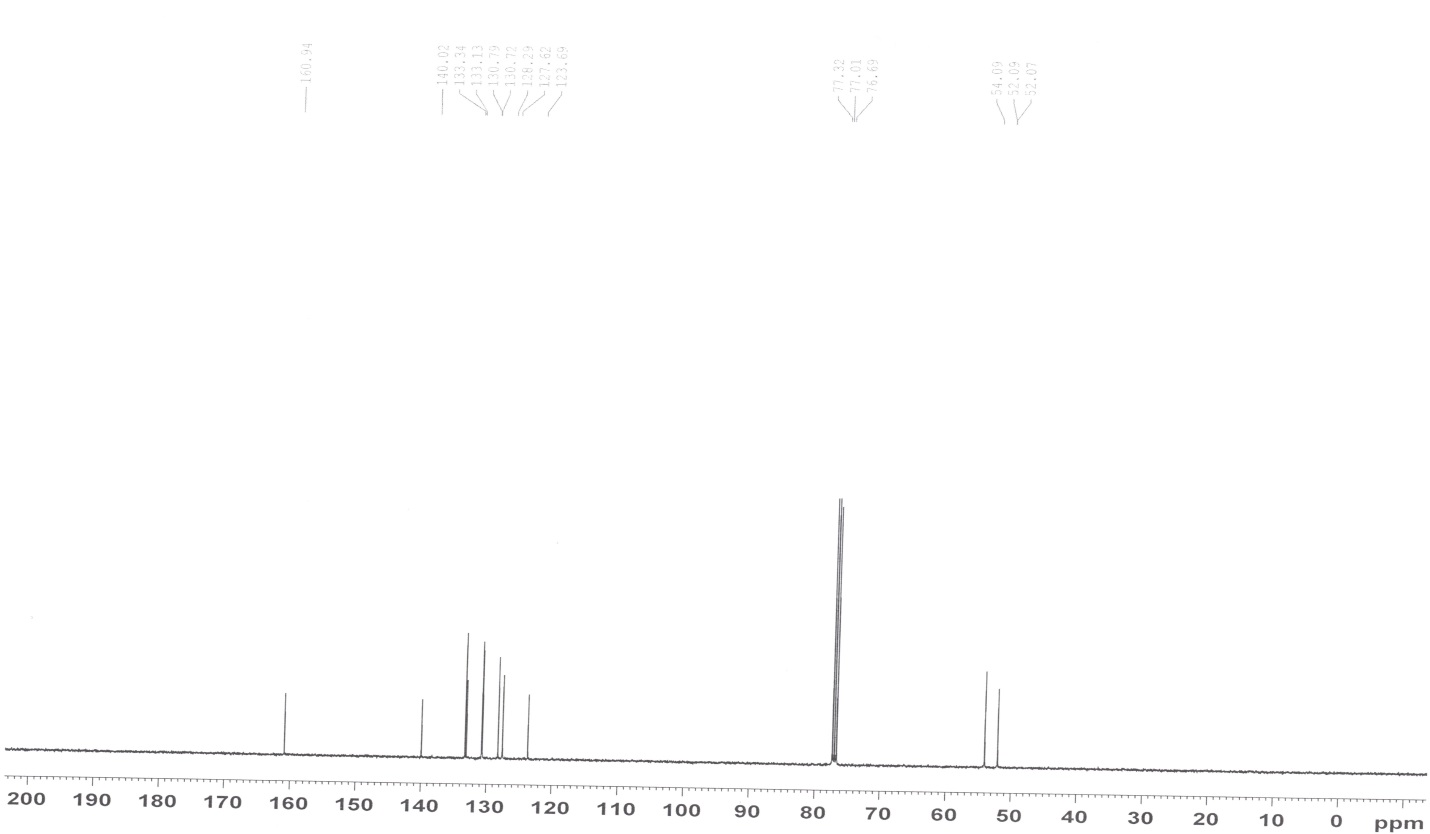


**Figure S44**. ^13^C NMR spectrum of **5bj** in CDCl_3_


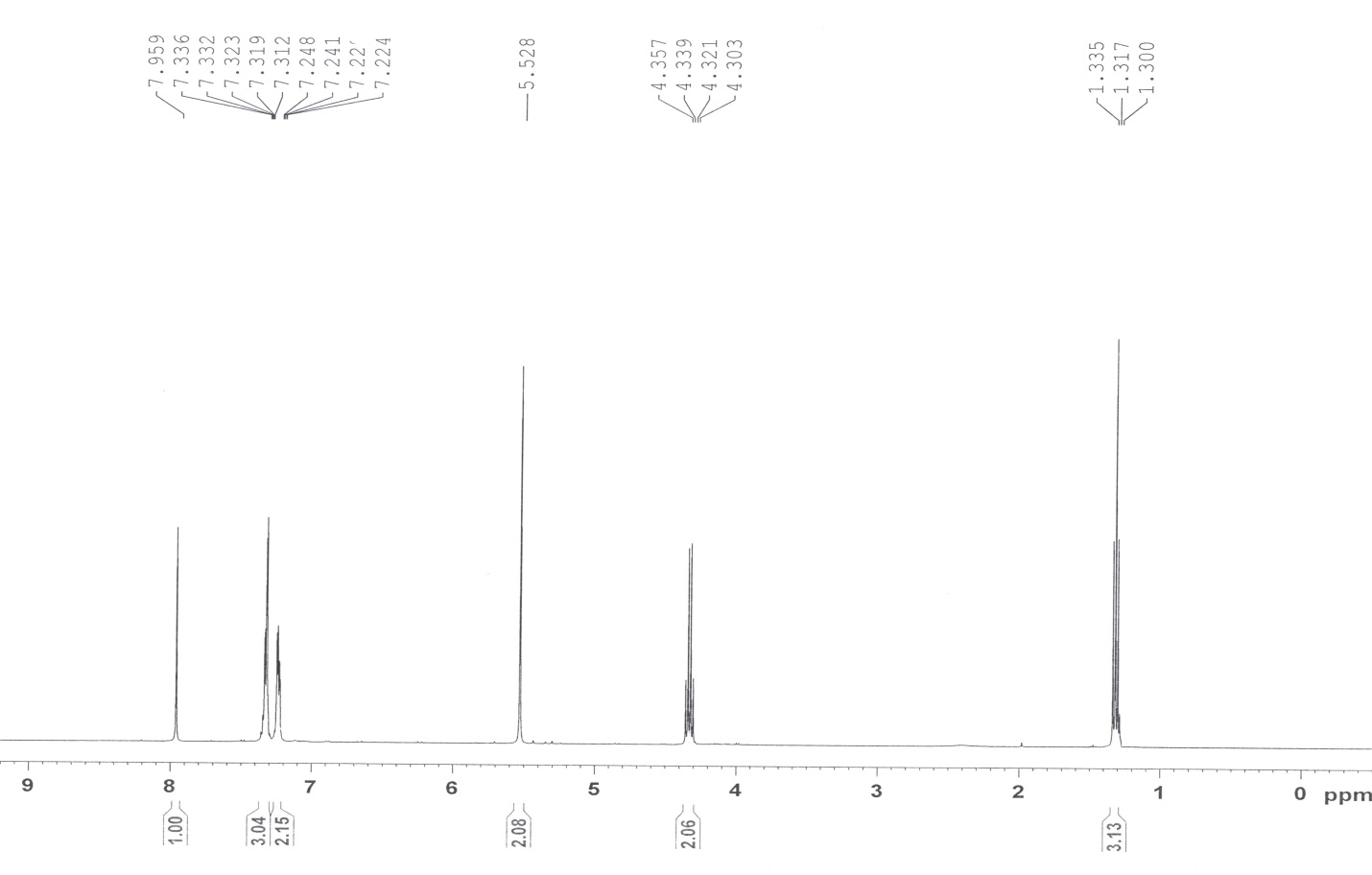


**Figure S45**. ^1^H NMR spectrum of **5ak** in CDCl_3_


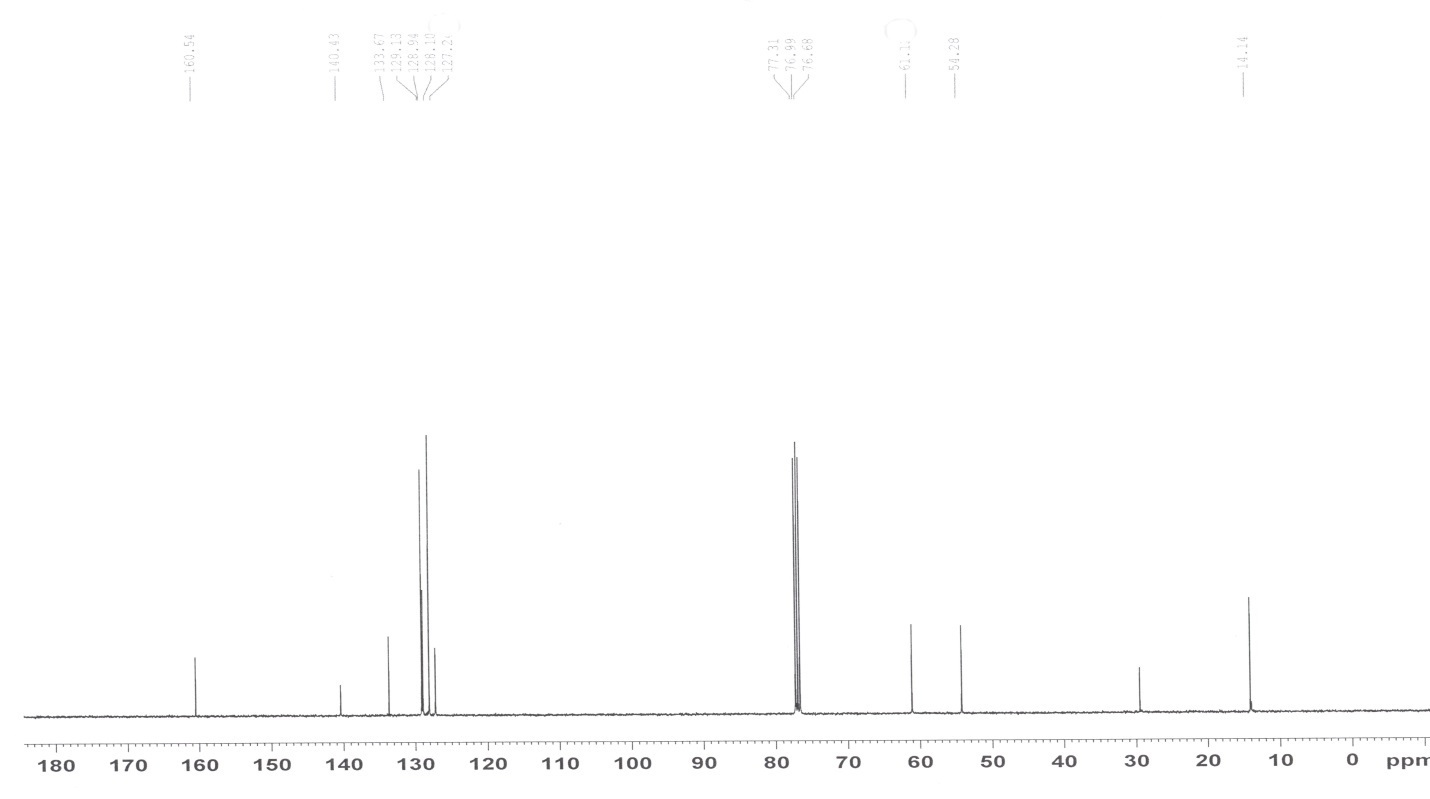


**Figure S46**. ^13^C NMR spectrum of **5ak** in CDCl_3_


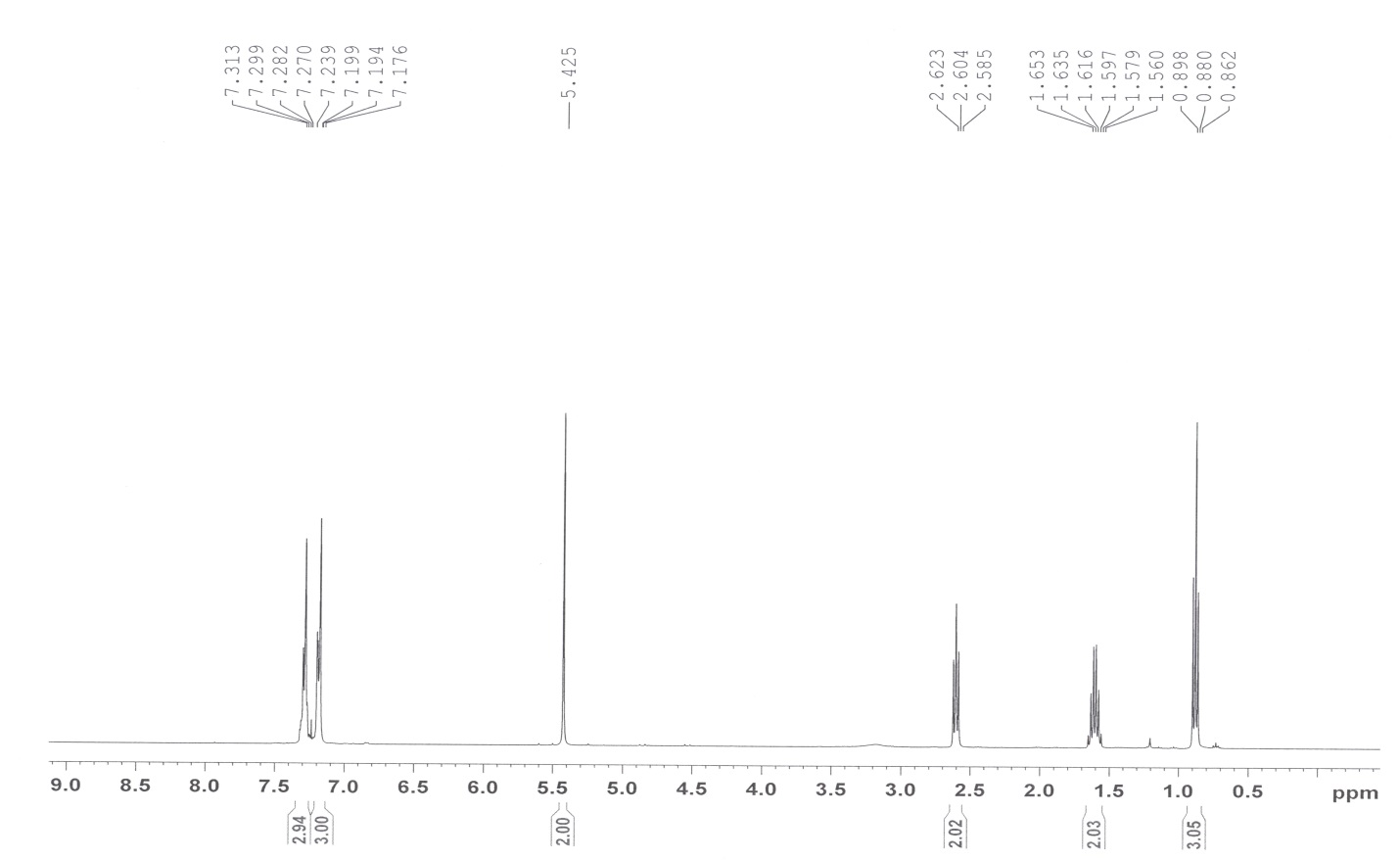


**Figure S47**. ^1^H NMR spectrum of **5al** in CDCl_3_


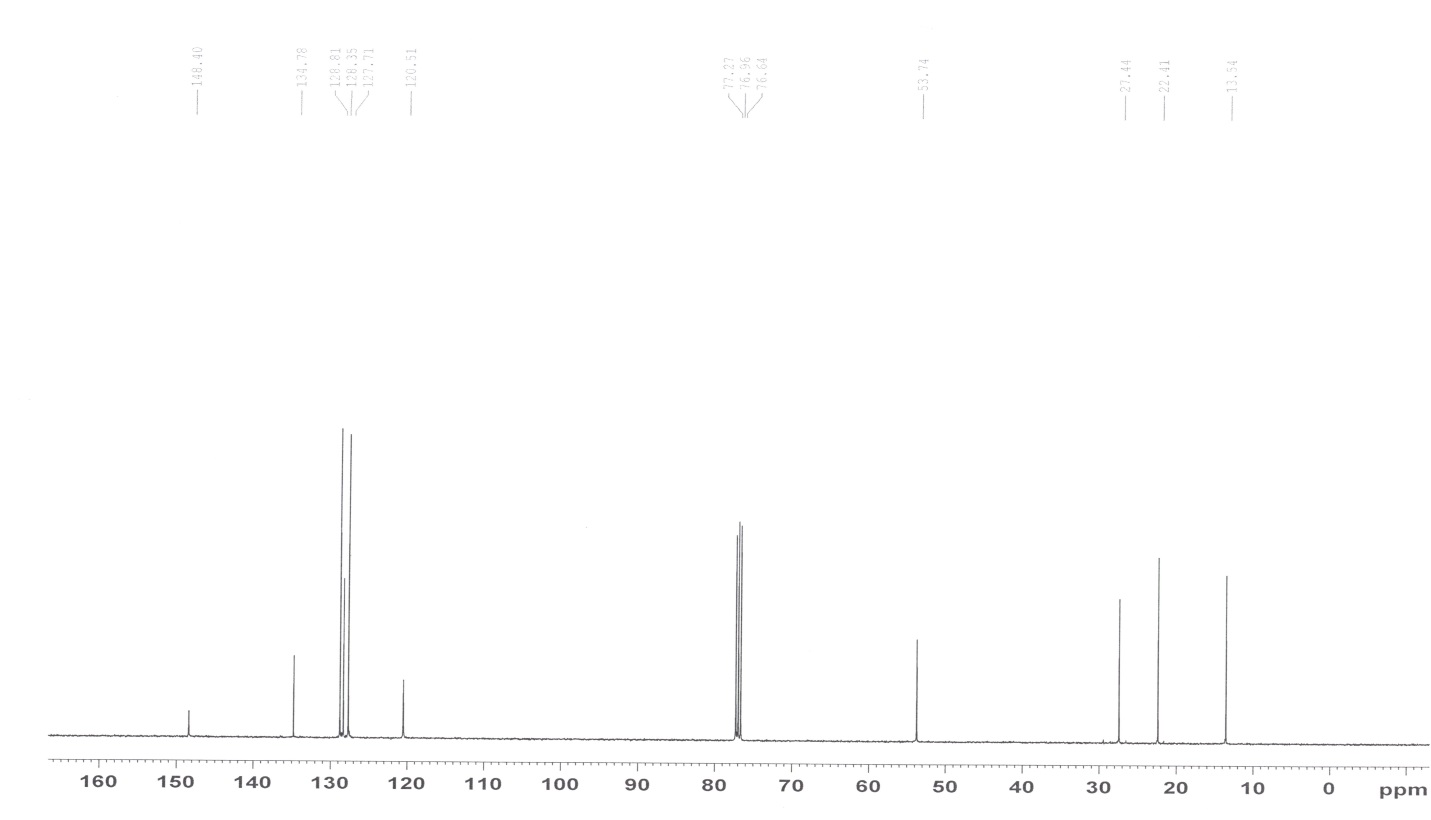


**Figure S48**. ^13^C NMR spectrum of **5al** in CDCl_3_


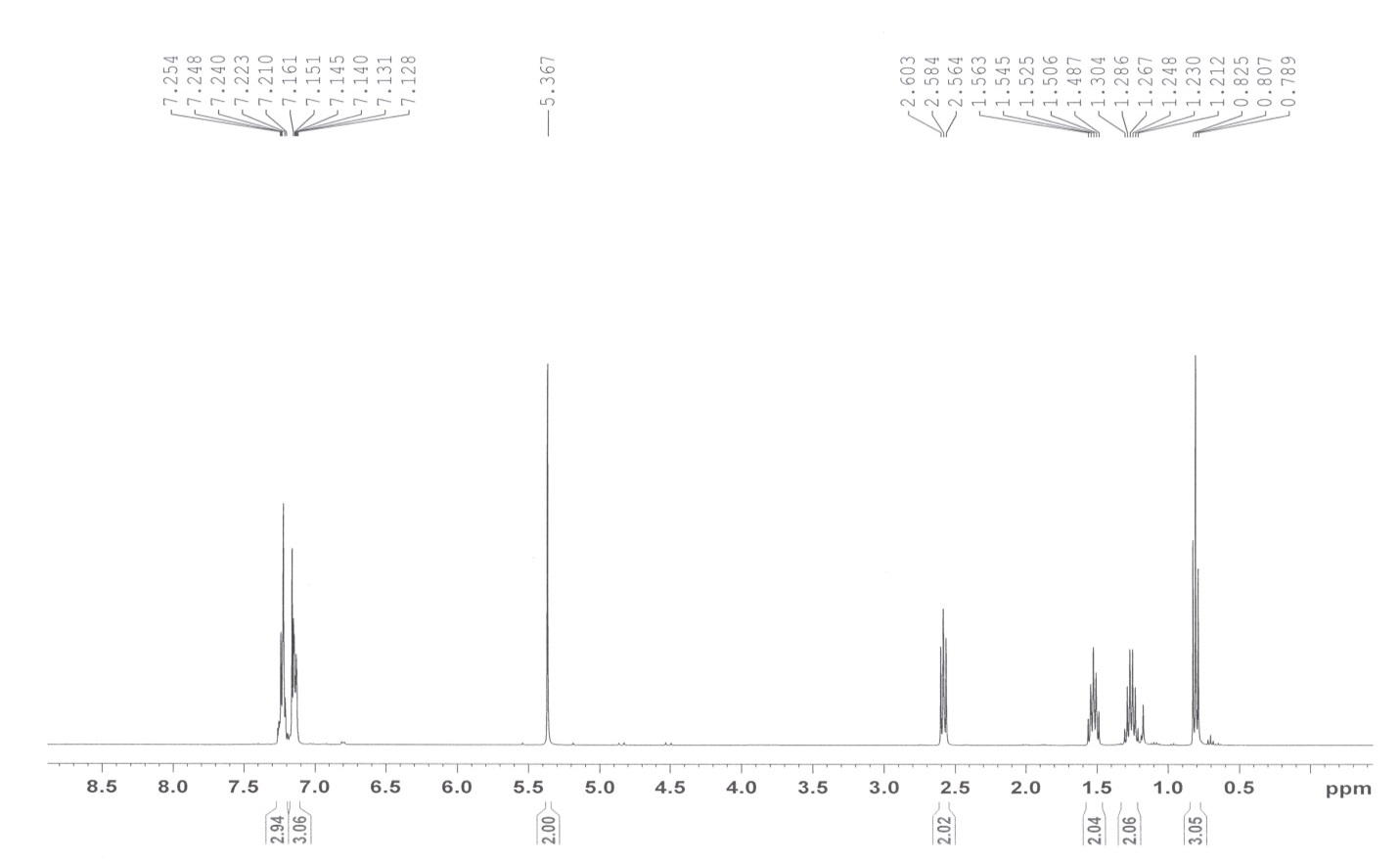


**Figure S49**. ^1^H NMR spectrum of **5am** in CDCl_3_


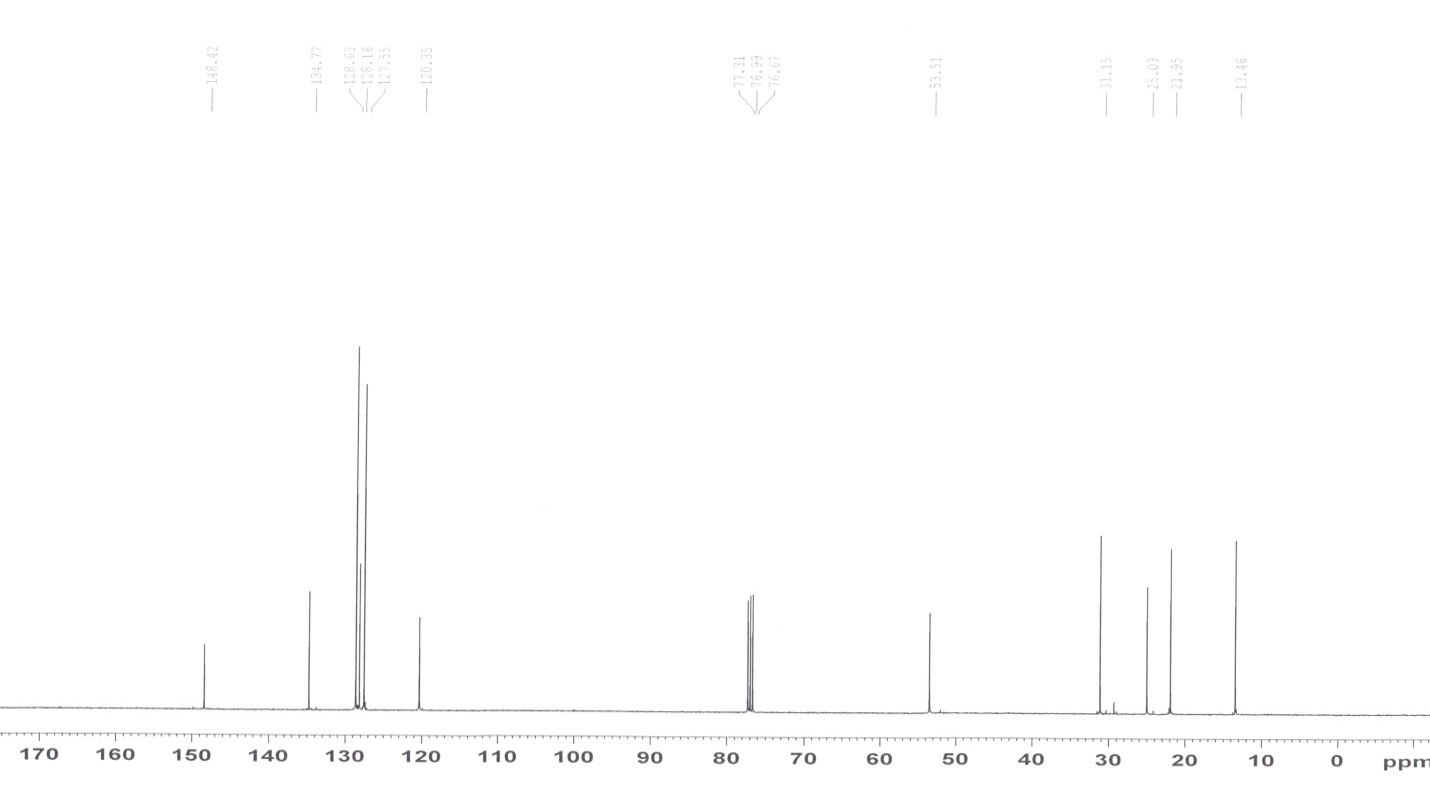


**Figure S50**. ^13^C NMR spectrum of **5am** in CDCl_3_
